# Remote allosteric control of single-atom Cu sites in Zr-MOFs enables selective CH_4_ hydroxylation

**DOI:** 10.1126/sciadv.aei9401

**Published:** 2026-10-14

**Authors:** Geqian Fang, Fenfei Wei, Wenjun Yu, Yu Kang, Maya Marinova, Lulu Chen, Lin Li, Vitaly V. Ordomsky, Anne-Marie Blanchenet, Sen Lin, Jian Lin, Xiaodong Wang, Xianzhi Fu, Andrei Y. Khodakov, Tao Zhang

**Affiliations:** ^1^ CAS Key Laboratory of Science and Technology on Applied Catalysis, Dalian Institute of Chemical Physics, Chinese Academy of Sciences, Dalian 116023, China.; ^2^ Univ. Lille, CNRS, Centrale Lille, Univ. Artois, UMR 8181 – UCCS – Unité de Catalyse et Chimie du Solide, Lille F-59000, France.; ^3^ State Key Laboratory of Chemistry for NBC Hazards Protection, College of Chemistry, Fuzhou University, Fuzhou 350116, China.; ^4^ Key Laboratory of Mesoscopic Chemistry of MOE, School of Chemistry and Chemical Engineering, Nanjing University, Nanjing, 210023, China.; ^5^ State Key Laboratory of Photocatalysis on Energy and Environment, College of Chemistry, Fuzhou University, Fuzhou 350002, China.; ^6^ University of Chinese Academy of Sciences, Beijing 100049, China.; ^7^ Suzhou Institute of Nano-Tech and Nano-Bionics, Chinese Academy of Sciences, Suzhou, 215123, China.; ^8^ Institut Michel-Eugène Chevreul, Villeneuve-d’Ascq 59655, France.

## Abstract

Highly selective oxidation of methane to liquid oxygenates under mild conditions remains challenging because of the difficulty in controlling overoxidation at highly reactive sites. Here, we demonstrate remote control of single-atom Cu reactivity through outer-shell coordination engineering in Zr-based metal-organic frameworks (MOFs). By anchoring Cu_1_ to Zr-oxo nodes in UiO-66-X (X = -NO_2_, -H, and -CH_3_), remote X-BDC ligands systematically modulate the electronic properties of Cu_1_ sites and the catalytic microenvironment. In situ characterization combined with ab initio molecular dynamics in an explicitly solvated aqueous environment reveals a dynamic outer-shell-to-inner-shell regulation that reshapes the active structure and reactive oxygen species around Cu_1_ sites, thereby facilitating CH_4_ activation. In particular, the electron-withdrawing NO_2_-BDC ligand creates electron-deficient Cu_1_-^•^OH* sites, while adsorbed water further regulates active-site conformation. Consequently, Cu_1_/U6-NO_2_ achieves a turnover frequency of 322.4 h^−1^ with nearly 100% oxygenate selectivity under mild conditions, establishing a general strategy for overcoming activity-selectivity trade-offs in methane conversion.

## INTRODUCTION

The direct selective oxidation of methane (DSOM) to liquid oxygenates represents an attractive strategy for valorizing abundant methane resources while reducing greenhouse gas emissions. (*1*) However, this transformation faces notable challenges stemming from the energy demanding requirements for activating inert C-H bonds, while preventing product overoxidation. (*2*) This intrinsic activity-selectivity trade-off effect has severely hindered practical applications. Nature addresses this challenge through methane monooxygenases (MMOs), (*3*) where atomically dispersed Cu/Fe-oxo centers enable efficient methane activation under ambient conditions with perfect selectivity. (*4*–*6*) Inspired by these biological systems, extensive efforts have focused on developing nano-metal catalysts (Fe, Cu, Rh, Au, Pd, etc.). (*7*–*10*) Nevertheless, these systems universally struggle with product overoxidation due to the heterogeneity of active sites, underscoring the imperative for structurally uniform catalysts specific for selective methane oxidation reactions.

Single-atom catalysts (SACs) have recently opened avenues for methane activation, leveraging their unique advantage of atomic-level dispersion and maximum metal utilization efficiency to enable catalytic transformations under mild conditions. (*11*) Promising results have been achieved for DSOM using SACs with H_2_O_2_, (*7*) which is a more environmentally benign oxidant compared with strong-acid or pollution-prone oxidants such as persulfate and N_2_O. (*12*) However, current strategies for SAC design have focused predominantly on engineering the inner coordination shell of the metal center. Conventional strategies involving heteroatom doping or lattice embedding often cause undesirable structural perturbations or active-site blocking. (*13*) In contrast, the substantial potential of outer-shell or second-shell environments remains largely unexplored, despite emerging evidence that peripheral modifications of single atoms can profoundly determine both alkane activation and product distribution. (*14*, *15*) This insight makes outer-shell engineering a powerful tool for achieving precise, long-range control of single-atom reactivity in methane hydroxylation.

Metal-organic frameworks (MOFs) provide an ideal platform for stabilizing single-atom sites, featuring a well-defined porous structure and precisely tunable physicochemical microenvironment. (*16*–*18*) Their periodic arrangement of metal-oxo nodes and organic ligands enables the control of anchored single atoms’ electronic states through both short-range coordination and long-range ligand effects. This hierarchical structural control mimics the regulatory mechanisms of enzymatic systems, as observed in MMO, (*19*) imidazole glycerol phosphate synthase (IGPS), (*20*) and other enzymes, (*21*, *22*) where precise spatial organization governs catalytic activity. Striking resemblance to enzyme microenvironments has made hybrid organic-inorganic MOFs a natural platform for enzyme immobilization. (*23*) Translating this principle to heterogeneous catalysis could enable remote regulation of single-atom active sites and help overcome the activity–selectivity trade-off in DSOM.

Herein, we develop a series of precisely controlled Cu_1_/UiO-66-X catalysts [denoted as Cu_1_/U6-X, (X = -NO_2_, -H, and -CH_3_)] prepared by anchoring Cu single atoms to Zr-oxo nodes in U6-X MOFs for DSOM, leveraging the material’s hydrothermal stability and tunable microenvironment. (*24*–*27*) Remarkably, the remote electron-withdrawing effect of the 2-Nitro–1, 4-benzenedicarboxylic (NO_2_-BDC) ligand dramatically boosts oxygenate yield, achieving an exceptional turnover frequency (TOF) of 322.4 h^−1^, 2.3 times higher than that on the electron-donating CH_3_-BDC counterpart, while maintaining near 100% selectivity, surpassing most reported SACs. Combined experimental characterizations and theoretical modeling reveal that outer-shell X-BDC ligands fine-tune the DSOM performance via framework-mediated electronic effects. A combination of techniques confirms the selective generation of ^•^OH and ^•^OOH reactive species. Peripherally adsorbed H_2_O can further dynamically modulate the electron-deficient Cu_1_-^•^OH* active sites in Cu_1_/U6-NO_2_, enabling efficient methane activation and selective conversion to oxygenates through continuous structural adjustment.

## RESULTS

### Synthesis and Characterization of Cu_1_/U6-X Catalysts

The Cu_1_/U6-X (X = -NO_2_, -H, and -CH_3_) catalysts feature tunable functional groups at the outer shells of the Zr-oxo nodes (Fig. 1A). The U6-X MOFs were synthesized via a solvothermal reaction using X-BDC and ZrCl_4_, with HCl as a modulator. Subsequently, the Cu_1_/U6-X catalysts with different Cu loadings (table S1) were prepared via an impregnation-reaction method, (*28*) where CuCl_2_·2H_2_O precursor was dissolved in *N*, *N*-dimethylformamide (DMF) at 85°C for 24 h. Nuclear magnetic resonance (NMR, fig. S1) and thermogravimetric analysis (TGA, fig. S2) show that the Zr-oxo node of U6-X contains ligand defects, which are predominantly capped by -OH/OH_2_ groups. (*29*, *30*)

**Fig. 1.**
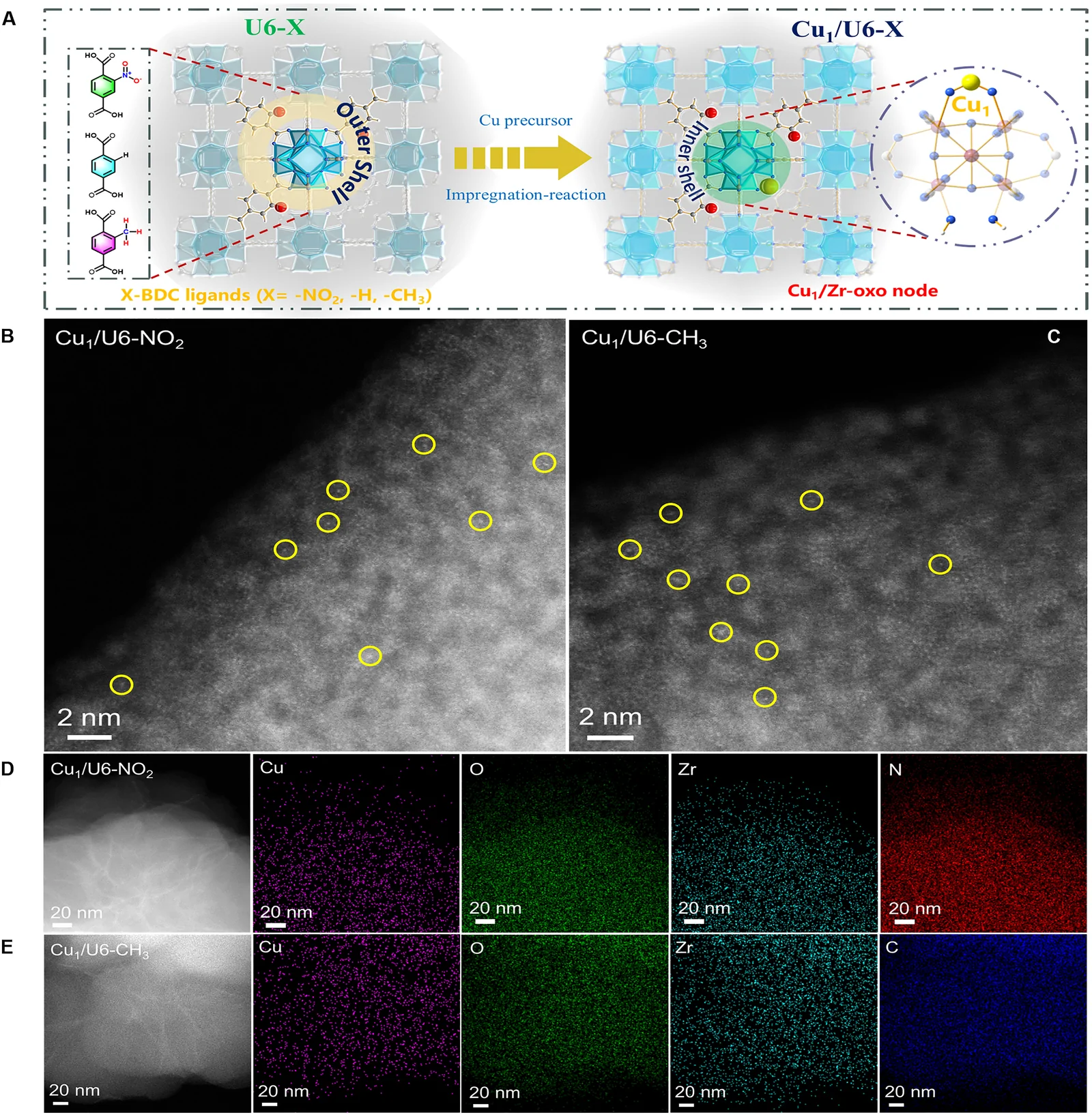
The design and morphology of various Cu_1_/U6-X catalysts. (**A**) Schematic illustration of U6-X anchoring Cu_1_ sites via impregnation-reaction method. (**B**), (**C**) Representative AC-HAADF-STEM images with (**D**), (**E**) corresponding EDS elemental mappings for 0.56Cu_1_/U6-NO_2_, and 0.48Cu_1_/U6-CH_3_ catalysts. Possible Cu_1_ species are indicated by yellow circles.

To obtain a better signal-to-noise ratio for structural characterization, Cu_1_/U6-X catalysts with a higher Cu loading of 0.48 – 0.67 wt.% were selected for analysis. Scanning electron microscopy (SEM) images (fig. S3) display an intergrown octahedral morphology with crystal sizes ranging from 150 to 500 nm. Powder X-ray diffraction (XRD) patterns (fig. S4) confirm that the crystallinity of U6-X is maintained after Cu incorporation. Attenuated total reflectance infrared (ATR-IR) spectroscopy (fig. S5) clearly shows characteristic vibrational peaks of outer-shell ligands at 1540 cm^−1^ (NO_2_ asymmetric stretch) and 1406 cm^−1^ (CH_3_ asymmetric bending), confirming the successful synthesis of Cu_1_/U6-X. Aberration corrected high-angle annular dark-field scanning transmission electron microscopy (AC-HAADF-STEM) images (Fig. 1, B and C and fig. S6, A and B) reveal numerous atomically dispersed bright spots in Cu_1_/U6-X. In thinner regions, some of these features may be tentatively attributed to Cu_1_ species, although distinguishing them from Zr atoms remains challenging due to the strong Z-contrast. (*31*) No Cu nanoparticles are observed, and energy dispersive X-ray spectroscopy (EDS) mapping demonstrates homogeneous distribution of Cu species throughout Cu_1_/U6-X (Fig. 1, D and E and fig. S6C). Semi-quantitative EDS analysis shows Cu contents in the catalysts that follow a trend consistent with the ICP-OES results (table S2). Diffuse reflectance infrared Fourier transform spectra (DRIFTS) show a pronounced decrease in the intensity of -OH group stretching vibrations band in Cu_1_/U6-X compared to U6-X, suggesting that Cu single atoms preferentially occupy the -OH sites on Zr-oxo nodes (fig. S7).

While the catalysts maintain similar morphology and crystallinity, X-ray photoelectron spectroscopy (XPS) and X-ray absorption spectroscopy (XAS) reveal pronounced ligand-induced modifications of the electronic structure of Cu active centers. The Cu *2p* XPS spectra demonstrate distinct binding energy shifts: +0.4 eV for Cu_1_/U6-NO_2_ and − 0.2 eV for Cu_1_/U6-CH_3_ relative to Cu_1_/U6-H (Fig. 2A). A similar shift of energy positions is observed in their normalized Cu *k*-edge X-ray absorption near-edge structure (XANES) spectra (Fig. 2B). Notably, the Cu atoms mainly exist as Cu^δ+^ species according to their XPS binding energies while their Cu *k*-edge XANES absorption edge is shifted to higher energy than that of CuO. These results unambiguously demonstrate that outer-shell ligands can effectively modulate the electronic properties of Cu_1_ centers, with NO_2_-BDC decreasing and CH_3_-BDC increasing the electron density of Cu_1_ sites.

**Fig. 2.**
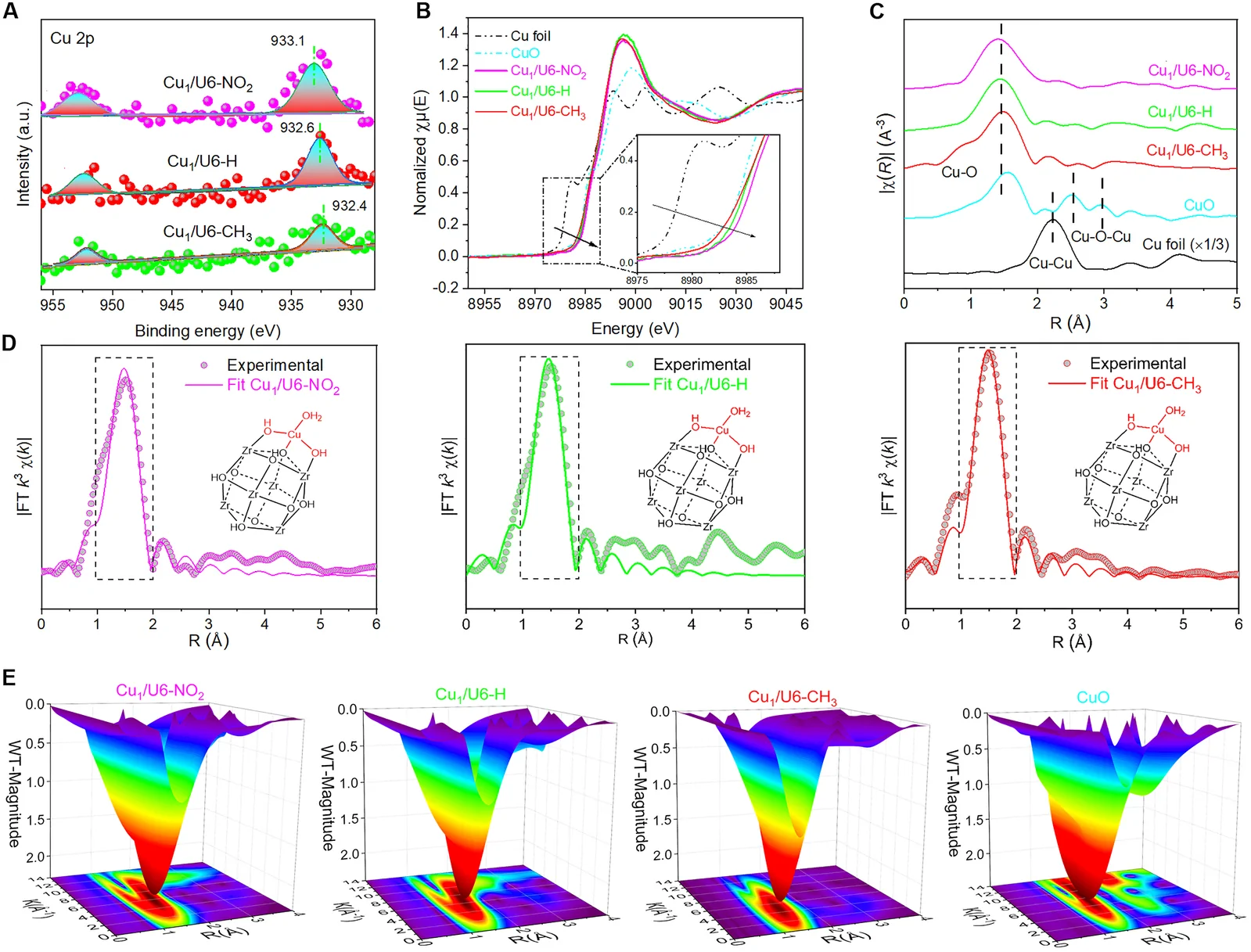
The electronic and coordination structure of Cu_1_/U6-X. (**A**) Cu *2*p** XPS spectra over 0.56Cu_1_/U6-NO_2_, 0.67Cu_1_/U6-H, and 0.48Cu_1_/U6-CH_3_; (**B**) Cu *k*-edge XANES spectra with Cu foil and CuO as references, (**C**) *k^2^*-weighted Fourier transform spectra derived from EXAFS results of the Cu *k*-edge with Cu and CuO as references, (**D**) *R*-space EXAFS fitting results (inset: proposed structural model of Cu_1_ sites anchored onto Zr-oxo nodes) and (**E**) WT plots for 0.56Cu_1_/U6-NO_2_, 0.67Cu_1_/U6-H, and 0.48Cu_1_/U6-CH_3_ with CuO reference.

Fourier transform extended X-ray absorption fine structure (EXAFS) analysis shows that all the Cu_1_/U6-X samples exhibit a dominant peak at ∼1.5 Å (Cu-O) and lack the characteristic 2.3 Å peak (Cu-Cu) characteristic of copper clusters, thus confirming single-atom dispersion of Cu species (Fig. 2C). Quantitative fitting of EXAFS data reveals four-coordinated Cu sites with average Cu-O bond lengths of ∼1.95 Å on Cu_1_/U6-NO_2_ (Fig. 2D and fig. S8), while Cu_1_/U6-H and Cu_1_/U6-CH_3_ show similar coordination with slightly shorter Cu-O bond lengths of ∼1.93 and ∼ 1.94 Å, respectively (table S3). Wavelet transform (WT) plots of the Cu *k*-edge of Cu_1_/U6-X show only a single intensity maximum at ∼1.5 Å (Fig. 2E), in contrast to the Cu-O-Cu and Cu-Cu contributions observed in the WT plots of CuO and Cu foil. The EXAFS and WT analysis combined with AC-HAADF-STEM and EDS provide conclusive evidence for the exclusive presence of atomically dispersed Cu species in Cu_1_/U6-X. Moreover, taking Cu_1_/U6-NO_2_ as a representative example, XRD, ATR-IR, and XPS results show that the Cu_1_ species and MOF framework are well preserved across different Cu loadings (figs. S9 to S11), confirming the high structural consistency of the composites. These results clearly demonstrate that the inner-shell Cu single-atom sites adopt well-defined coordination geometries with the Zr-oxo nodes, while the outer-shell X-BDC ligands precisely modulate the electronic configuration of the Cu_1_ centers.

### DSOM Performance

The performance of Cu_1_/U6-X catalysts for DSOM was investigated at 60°C in an autoclave reactor under 3 MPa CH_4_ using a dilute H_2_O_2_ solution. The pristine U6-X supports exhibit limited methane hydroxylation activity (Fig. 3A). To systematically evaluate the influence of outer-shell X-BDC ligands on the catalytic behavior of inner-shell Cu_1_ sites, Cu_1_/U6-X catalyst series with low, medium, and high Cu loadings were tested. The oxygenate yield generally increases with increasing Cu loading, indicating that the number of accessible Cu_1_ sites plays an important role in the overall product formation rate. For instance, the addition of 0.35 wt.% Cu_1_ species to U6-NO_2_ dramatically enhances catalytic performance, with an oxygenate yield of 4191.5 μmol g_cat_^−1^ h^−1^ and nearly 100% selectivity, representing a ten-fold improvement over the U6-NO_2_ supports. Comparative analysis reveals lower yield and selectivity of oxygenates for 0.35Cu_1_/U6-CH_3_ (2573.9 μmol g_cat_^−1^ h^−1^, 90.0%) and 0.33Cu_1_/U6-H (3656.7 μmol g_cat_^−1^ h^−1^, 94.4%), demonstrating pronounced ligand-dependent activity variations. Notably, CH_3_OH and CH_3_OOH account for >91% selectivity on the Cu_1_/U6-NO_2_ catalysts, regardless of Cu loading.

**Fig. 3.**
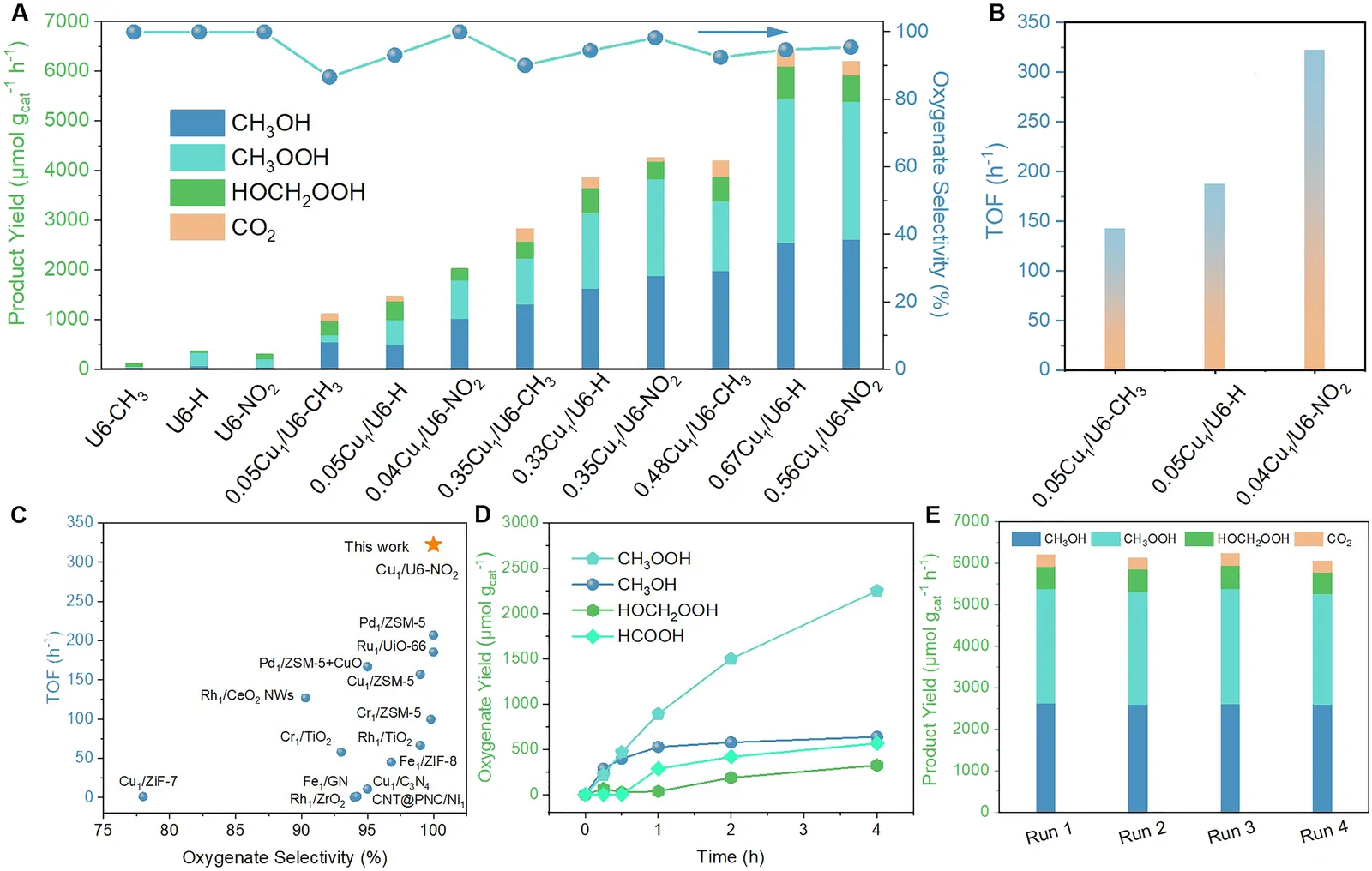
The DSOM performance over Cu_1_/U6-X catalysts. (**A**) Yield and selectivity of oxygenates for the DSOM reaction over Cu_1_/U6-X catalysts. (**B**) TOF of oxygenate formation over Cu_1_/U6-X catalysts. (**C**) Comparison of the catalytic performance for the DSOM reaction between Cu_1_/U6-NO_2_ and other SACs. (**D**) Time-on-line profile of oxygenates during the DSOM reaction over 0.04Cu_1_/U6-NO_2_. (**E**) Reusability of 0.56Cu_1_/U6-NO_2_ in the DSOM reaction over multiple cycles.

The TOF values calculated on the basis of the number of Cu_1_ sites at different Cu loadings exhibit a consistent trend (fig. S12). Notably, 0.04Cu_1_/U6-NO_2_ achieves a TOF as high as 322.4 h^−1^ while maintaining nearly 100% oxygenate selectivity (Fig. 3B). This shows ∼1.7- and ∼2.3-fold increases in the reaction rate compared to Cu_1_/U6-H and Cu_1_/U6-CH_3_, with a similar copper content, respectively. To account for the volume effect of these ligands, we normalized oxygenate yield by the surface area (table S4), Cu_1_/U6-NO_2_ remained the top performer (fig. S13). These results unequivocally demonstrate the critical influence of outer-shell X-BDC ligands on enhancing oxygenate yield and selectivity of Cu_1_ single-atom sites for the DSOM reaction. Remarkably, the Cu_1_/U6-X catalysts in this work surpass the performance of state-of-the-art SACs (*8*, *9*, *25*, *32*–*49*), including Pd_1_/ZSM-5, (*38*) Fe_1_/ZIF-8, (*48*) and Cu_1_/C_3_N_4_ (*43*) (Fig. 3C and table S5), establishing benchmarks for methane hydroxylation.

Time-dependent product analysis of the optimal 0.04Cu_1_/U6-NO_2_ catalyst, which exhibits the highest TOF, reveals CH_3_OOH as the predominant oxygenate, which continuously accumulates throughout the reaction (Fig. 3D and figs. S14 to S16). To elucidate the carbon source of oxygenates, we compared the oxygenate formation in N_2_ and CH_4_ atmospheres. Oxygenates such as CH_3_OOH, CH_3_OH, HOCH_2_OOH and HCOOH were detected under CH_4_ (table S6), whereas no detectable oxygenates were observed under N_2_ over various catalyst composites or ligands. Herein, a weak oxygenate signal observed on Cu_1_/U6-CH_3_ probably stems from partial oxidation of the CH_3_-BDC ligands during the reaction. However, this residual contribution is negligible after background subtraction. To further verify the carbon origin, we carried out a controlled experiment using ^13^CH_4_ as the feed gas. As shown in fig. S17, isotopically labeled oxygenates (^13^CH_3_OOH, ^13^CH_3_OH, HO^13^CH_2_OOH and ^13^HCOOH) were detected, confirming that the oxygenated products derive from the CH_4_ oxidation rather than the catalyst itself. These results demonstrate that the presence of NO_2_-BDC in U6-NO_2_ substantially amplifies the oxygenate production over Cu_1_ sites.

Beyond catalytic activity and selectivity, stability is critical for potential applications. The 0.56Cu_1_/U6-NO_2_ catalyst with higher Cu loading exhibits stable catalytic performance (Fig. 3E) over four operating cycles, maintaining nearly constant oxygenate yield and selectivity. A combination of characterization techniques confirms the stability of catalyst structure after conducting methane oxidation. ICP-OES analysis of the spent Cu_1_/U6-NO_2_ catalyst reveals negligible Cu leaching, with the metal loading nearly identical to that of the fresh catalyst (table S1). The XRD results (fig. S18) confirm that the crystallinity of the U6-X catalysts remains intact after the reaction. ATR-IR characterization (fig. S19) further demonstrates the structural stability, particularly through the persistence of the characteristic -NO_2_ stretching vibration at 1540 cm^−1^ in Cu_1_/U6-NO_2_. SEM results confirm that the morphology of Cu_1_/U6-NO_2_ remains unchanged after the stability test (fig. S3). Correspondingly, the BET surface areas and the crystalline structure are also preserved (table S4 and fig. S20). AC-HAADF-STEM and EDS mapping images (fig. S21, A and B) of the spent Cu_1_/U6-NO_2_ catalyst further confirm the maintenance of Cu single atoms. Additionally, XAFS (fig. S22) and Raman spectroscopy (fig. S23) confirm that the chemical state of the Cu_1_ sites and the electronic properties of the Zr-oxo nodes remain unaltered after the reaction. All these results highlight the robust stability of the Cu_1_/U6-NO_2_ catalyst, with its geometric, electronic structures and catalytic performance preserved during the DSOM reaction.

### Underlying DSOM Mechanism

For mechanistic investigations, representative samples with relatively higher Cu loadings, including 0.56Cu_1_/U6-NO_2_ and 0.48Cu_1_/U6-CH_3_, were selected. Quasi in situ electron paramagnetic resonance (quasi in situ EPR) spectroscopy with 5, 5′-dimethyl-1-pyrroline N-oxide (DMPO) trapping identifies critical radical intermediates. (*50*) The ^•^OOH signal intensity on Cu_1_/U6-NO_2_, which exceeds that on Cu_1_/U6-CH_3_ by approximately 50% (Fig. 4A and table S7), indicates the NO_2_-BDC ligand’s ability to promote ^•^OOH generation. This enhanced ^•^OOH formation directly correlates with the observed CH_3_OOH predominance. Concurrently, Cu_1_/U6-NO_2_ exhibits the reduced ^•^OH concentration relative to Cu_1_/U6-CH_3_ (Fig. 4B and tables S7 and S8), which reduces the overall methane activation, but is helpful to suppress the undesired over-oxidation pathways. (*51*) In addition, the ^•^CH_3_ radicals were not detected by EPR, probably because of their high reactivity. (*37*) These findings demonstrate that the outer-shell NO_2_-BDC ligand preferentially promotes the formation of mild ^•^OOH intermediates while reducing the concentration of overly reactive ^•^OH species, (*51*) whose higher levels lead to methane overoxidation.

**Fig. 4.**
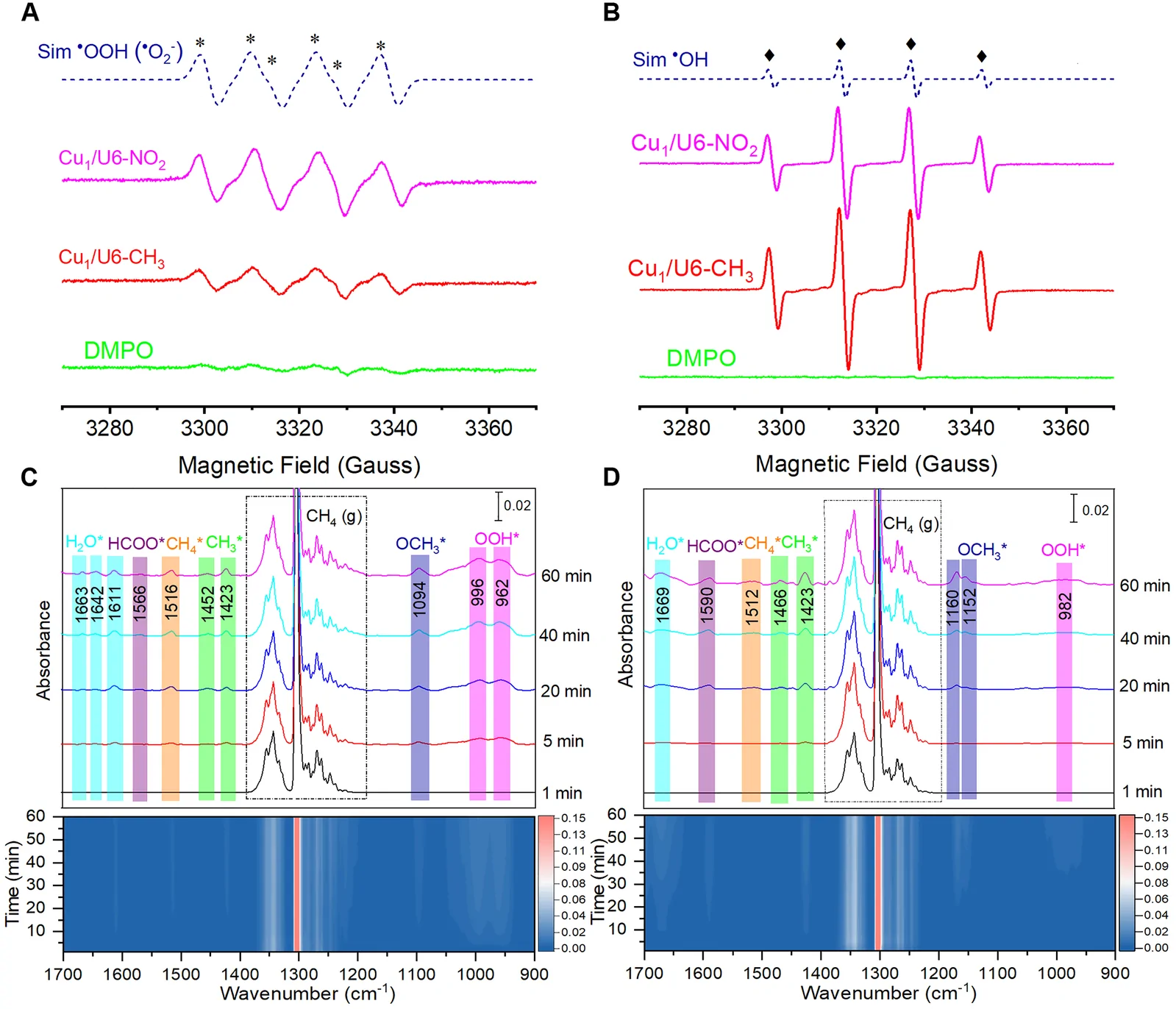
Identification of reactive intermediate species. Quasi in situ EPR spectra of (**A**) •OOH (•O_2_^−^), and (**B**) •OH trapped by DMPO over 0.56Cu_1_/U6-NO_2_ and 0.48Cu_1_/U6-CH_3_ catalysts; In situ DRIFTS spectra of (**C**) 0.56Cu_1_/U6-NO_2_ and (**D**) 0.48Cu_1_/U6-CH_3_ catalysts at 60°C during simultaneous introduction of CH_4_ and H_2_O_2_ reactants. (bottom: time evolution of adsorbed species contour figure; top: DRIFTS spectra of adsorbed species at 1 h).

To further gain insight into the influence of outer-shell structure on DSOM performance, we conducted in situ DRIFTS measurements. Upon methane introduction, Cu_1_/U6-X exhibits a characteristic ν_4_ bending band of gaseous CH_4_ at 1304 cm^−1^ (fig. S24). Upon the co-introduction of H_2_O_2_ and CH_4_, Cu_1_/U6-NO_2_ shows stronger bands associated with hydroperoxyl species (OOH*) at 962 and 996 cm^−1^ (Fig. 4C). (*25*, *40*, *52*) In contrast, Cu_1_/U6-CH_3_ only shows a weak OOH* band at 982 cm^−1^ (Fig. 4D). (*53*–*55*) The adsorbed H_2_O vibration is observed at 1669 cm^−1^ on Cu_1_/U6-CH_3_ and red-shifts to 1663–1611 cm^−1^ on Cu_1_/U6-NO_2_, consistent with stronger hydrogen-bonding/adsorption of water on Cu_1_/U6-NO_2_. (*56*)

Interestingly, we find that the outer-shell–dominated water coordination at the Cu_1_ site can essentially modulate methane activation, yielding characteristic peaks at 1512 or 1516 cm^−1^ on Cu_1_/U6-X, assigned to activated CH_4_* (*57*). The bands at 1423, 1452, and 1466 cm^−1^ on Cu_1_/U6-X are assigned to CH_3_* rocking/scissoring vibrations. (*58*–*61*) In addition, the C-O stretching of OCH_3_* red-shifts to 1094 cm^−1^ on Cu_1_/U6-NO_2_, (*62*) compared to 1160 and 1152 cm^−1^ on Cu_1_/U6-CH_3_. (*63*, *64*) This shift results from the more electron-deficient nature of Cu single atoms in U6-NO_2_, which strengthens electron withdrawal from the methoxy oxygen, polarizing and elongating the C-O bond. (*65*) So, the downshift to 1094 cm^−1^ is consistent with a less electron-rich OCH_3_ species. Consequently, the reduced bond force constant lowers the C-O stretching vibration frequency. These results suggest that the produced oxygenic species enhance CH_4_ activation on Cu_1_/U6-X. The OOH* intensity positively correlates with the electron deficiency of Cu_1_ in Cu_1_/U6-X. Compared to Cu_1_/U6-NO_2_, upon simultaneous introduction of H_2_O_2_ and CH_4_, Cu_1_/U6-CH_3_ exhibits a greater tendency to decompose H_2_O_2_ into ^•^OH, leading to excessive oxidation of the oxygenate into a higher intensity of adsorbed formate (1590 cm^−1^) and ultimately to CO_2_.

In addition to oxygenic radical, adsorbed species on single-atom sites can modulate their electronic states to influence methane hydroxylation performance. (*49*) Further in situ DRIFTS temperature programmed desorption (in situ DRIFTS-TPD) measurements of co-adsorbed CH_4_ and H_2_O_2_ on Cu_1_/U6-X demonstrate distinct behaviors (fig. S25). The OCH_3_* signal at 1094 cm^−1^ on Cu_1_/U6-NO_2_ almost disappears at 120°C, while the signal at 1160 cm^−1^ persists on Cu_1_/U6-CH_3_, confirming that NO_2_-BDC functionalized catalyst facilitates oxygenate desorption reducing their residence time on Cu_1_ sites and over-oxidation. Moreover, this Cu_1_ valence modulation gains direct experimental verification through near ambient pressure XPS (NAP-XPS) (fig. S26), where water vapor exposure increases Cu 2*p* binding energy by 0.24 eV to 933.27 eV, with complete recovery to 933.0 eV after desorption. The quasi in situ EPR spectra, in situ DRIFTS and NAP-XPS results highlight the role of outer-shell ligands in fine-tuning the coordination environment between Cu_1_ sites and solvent H_2_O molecules, thereby steering the selective evolution of reactive hydroxyl species and hydroperoxyl intermediates in methane hydroxylation.

### Theoretical insights into remote allosteric effects

Our theoretical investigation elucidates how outer-shell ligands orchestrate catalytic behavior through framework-mediated electronic effects. As suggested by the XAFS and DRIFTS (Fig. 2D and fig. S7), the catalytic cycle originates from a Cu_1_-OH/H_2_O initial state. In this configuration, H_2_O_2_ abstracts a hydrogen atom to generate Cu_1_-OH/^•^OH* site in Cu_1_/U6-NO_2_ with a low energy barrier of 0.25 eV, releasing H_2_O and ^•^OH (fig. S27). This process involves dynamic H_2_O adsorption at the periphery of the Cu_1_-OH/^•^OH* site (fig. S28), underscoring the active participation of solvent molecules in the reaction mechanism.

Explicit-solvent ab initio molecular dynamics (AIMD) simulations reveal distinct water coordination environments induced by ligand electronic properties. The fact that the temperature and energy of the system fluctuate within a very narrow range during equilibration indicates that the solid–liquid interface has reached a stable equilibrium state for Cu_1_/U6-NO_2_ and Cu_1_/U6-CH_3_ (fig. S29). Within 10 ps, Cu_1_/U6-NO_2_ stabilizes a dynamically maintained bifacial hydration sphere comprising one chemisorbed and one physisorbed water molecule (Fig. 5A; movie S1). Analysis of the trajectory reveals that the two Cu_1_–O(H_2_O) distances fluctuate within a range of 2.25 to 3.75 Å. Specifically, the chemisorbed water maintains an average distance of 2.75 Å, while the physisorbed water exhibits a larger average distance of 3.16 Å. Despite these fluctuations, these two water molecules never fully dissociate from the first coordination layer (distances consistently remaining <3.5 Å for the majority of the trajectory), thereby ensuring a persistent bifacial configuration rather than a static one. This explains the red-shifted H_2_O vibration (1606 cm^−1^) unique to Cu_1_/U6-NO_2_ in DRIFTS spectra (Fig. 4C). In contrast, Cu_1_/U6-CH_3_ develops only a weaker monofacial hydration shell (3.07/3.35 Å, Fig. 5B; movie S2). This shows that peripheral ligands enable H_2_O adsorption and regulate the Cu_1_-^•^OH* intermediate (Fig. 5C). Analysis of Cu_1_-O(H_2_O*) distance evolution (Fig. 5D) confirms dynamic coordination while demonstrating substantially shorter time-averaged distances in Cu_1_/U6-NO_2_, indicating enhanced water binding capability conferred by NO_2_-BDC ligands. This strengthened coordination is visually apparent in the representative AIMD snapshots (fig. S30).

**Fig. 5.**
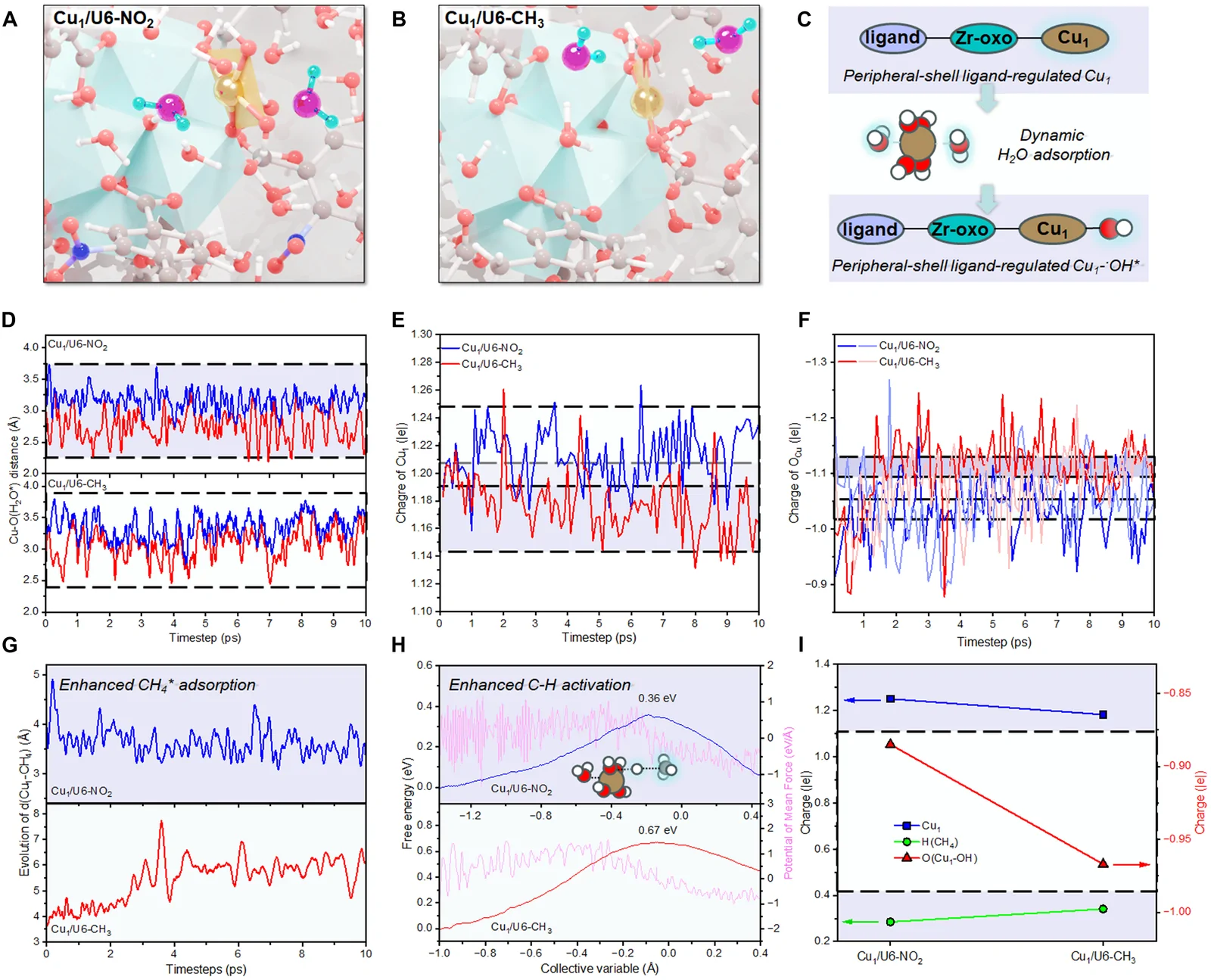
Theoretical insights into ligand-regulated catalytic behavior under aqueous conditions. (**A** and **B**) Equilibrium configurations of Cu_1_/U6-X (X = -NO_2_ and -CH_3_) from ab initio molecular dynamics (AIMD) simulations (10 ps, 333.15 K). (**C**) Peripheral ligands enable H_2_O adsorption and regulate the Cu_1_-^•^OH* intermediate. (**D**) Evolution of the two shortest Cu_1_-O(H_2_O) distances (excluding Cu_1_-^•^OH* bonds). Dynamic charge evolution of (**E**) Cu_1_ centers and (**F**) O atoms in Cu_1_-^•^OH* groups. (**G**) Cu_1_-CH_4_ distance variations during methane approach. (**H**) Free energy profiles for C-H activation. (**I**) Bader charges of key atoms in the transition state. Colors: H (white, cyan), C (gray), O (red, pink), N (blue), Cu (orange), Zr (cyan).

Electronic structure analysis uncovers the fundamental origin of these solvation differences. Bader charge analysis shows that the time-averaged Cu valence in Cu_1_/U6-NO_2_ remains consistently elevated compared to Cu_1_/U6-CH_3_ (Fig. 5E). (*66*) Crucially, we observe synchronized charge fluctuations across the entire catalytic assembly. This indicates that the electronic perturbation induced by the outer-shell NO_2_-BDC ligand is effectively transmitted to the remote Cu_1_ active site via the Zr-oxo node, thereby modulating its electrophilicity (fig. S31 and table S9). This electronic perturbation systematically enhances the electrophilicity of oxygen atoms in Cu_1_-^•^OH* along the series Cu_1_/U6-CH_3_ to Cu_1_/U6-NO_2_ (Fig. 5F), with NO_2_-BDC imparting maximal electron deficiency. Charge density difference analysis (fig. S32) confirms substantial electron transfer from Cu_1_ to water molecules, depleting Cu_1_ *d*-orbital occupancy and enhancing molecular adsorption capacity, consistent with the NAP-XPS results (fig. S26). The optimized electronic landscape intensifies substrate interactions, shortening Cu_1_-CH_4_ distance to 3.5 Å in Cu_1_/U6-NO_2_ versus 6.0 Å in Cu_1_/U6-CH_3_ (Fig. 5G, fig. S33, and movies S3 and S4).

The modification of the electronic structure manifests directly in CH_4_ activation kinetics. Slow-growth calculations quantitatively establish the ligand-dependent kinetics of CH_4_ activation via a radical-like transition state (TS), (*67*, *68*) revealing a 0.36 eV barrier for ^•^CH_3_ formation in Cu_1_/U6-NO_2_, 0.31 eV lower than in Cu_1_/U6-CH_3_ (0.67 eV) (Fig. 5H and fig. S34). This enhancement derives from synergistic optimization of transition state (TS) properties: structurally through shortened Cu_1_-O(H_2_O*) distances (2.71 Å versus 3.09 Å, fig. S35), and electronically via charge redistribution that renders Cu_1_ 0.07 |*e*| more positive while enhancing O atom electron deficiency by 0.08 |*e*| (Fig. 5I). These modifications reduce electron loss from the transferring H atom (∼0.29 |*e*| versus 0.34 |*e*|) and stabilize the TS configuration, evidenced by optimized C-H (1.21 Å) and H-O (1.40 Å) bond lengths compared to Cu_1_/U6-CH_3_ (1.23 Å and 1.29 Å). Additional projected density of states analysis is provided in figs. S36 and S37. Moreover, stabilization arises from the persistent presence of the water molecule, which dynamically approaches Cu_1_ from the side to form a stable Cu-O coordination bond during the C-H activation process (fig. S38). Ultimately, the overall oxidation pathway is illustrated in fig. S39. Methane is progressively oxidized to the methyl radical and subsequently branches into CH_3_OOH (ΔE_1_ = −3.72 eV) or CH_3_OH (ΔE_1_’ = −3.59 eV). Both pathways converge at HOCH_2_OOH via exothermic steps (ΔE_2_ = −3.26 eV and ΔE_2_’ = −3.39 eV), ultimately yielding HCOOH (ΔE_33_ = −2.30 eV). Notably, the thermodynamic favorability of CH_3_OOH formation over CH_3_OH, coupled with the diminishing exothermicity towards deep oxidation, well corroborates the experimentally observed species distribution (Fig. 3D).

This dual structural-electronic regulation exploits MOFs’ unique ability to propagate ligand effects across coordination spheres. The electron-withdrawing NO_2_-BDC group exerts long-range control by simultaneously reorganizing solvation environments and polarizing reaction coordinates, synergistically stabilizing TS geometry and electronic structure to effectively reduce the DSOM reaction barrier in Cu_1_/U6-NO_2_.

### Dynamic outer-shell effects steering selective methane oxidation

The target of nearly 100% oxygenate selectivity at high methane conversion has remained unachievable for supported transition-metal catalysts in DSOM, primarily stemming from insufficient control over the atomic-level catalytic microenvironments (*13*). While SACs deliver atomic precision, their performance hinges on both primary coordination and peripheral environments. (*15*, *69*) Our work circumvents these limitations through a precisely engineered Cu_1_/U6-X (X = -NO_2_, -H, and -CH_3_) system, where outer-shell ligands dynamically regulate the electronic states of inner-shell Cu_1_ sites via framework-mediated allosteric interactions (Fig. 2, A and B). The electron-withdrawing NO_2_-BDC ligand establishes optimal electronic conditions, yielding exceptional performance (322.4 h^−1^ TOF with near-complete selectivity) that surpasses most reported SACs (Fig. 3, A to C and table S5).

The U6-X platform transcends conventional supports (*e.g.*, metal oxides, zeolites) by harnessing enzyme-like precision in microenvironment engineering through framework-mediated allostery. (*24*, *26*) This synthetic regulation mirrors the exquisite active-site tuning in MMOs, realized herein via rational ligand design. Strikingly, the electron-withdrawing NO_2_-BDC ligand dynamically reshapes Cu_1_-^•^OH* sites, as unambiguously demonstrated through combined experimental and theoretical approaches. In situ DRIFTS reveals a red-shifted H_2_O vibration (1606 cm^−1^) and attenuated OCH_3_* (1094 cm^−1^) adsorption signals, demonstrating water-mediated stabilization of Cu_1_-^•^OH* sites (Fig. 4C). Complementary AIMD simulations further identify a bifacial hydration structure comprising one chemisorbed (Cu_1_-O = 2.75 Å) and one physisorbed (3.16 Å) water molecule (Fig. 5A; movie S1). This phenomenon originates from NO_2_-BDC induced increase in the Cu_1_ oxidation state, which enhances electron deficiency at Cu_1_ centers and strengthens H_2_O binding.

Combined theoretical studies (Fig. 5), in situ DRIFTS (Fig. 4, C and D), XANES (Fig. 2B), EXAFS (Fig. 2, C to E) and NAP-XPS (fig. S26) conclusively demonstrate that the NO_2_-BDC induced electron deficiency at Cu_1_-^•^OH* sites triggers two synergistic catalytic effects. First, the electron-withdrawing NO_2_-BDC ligand elevates the Cu_1_ valence state, rendering the Cu_1_ center more positive and the O center in Cu_1_-^•^OH* more electron-deficient compared to Cu_1_/U6-CH_3_. This charge redistribution substantially enhances the electrophilicity of the oxygen species for the activation of C-H bond in CH_4_ (*25*). Second, the elevated Cu_1_ valence state strengthens water coordination, optimizing the TS geometry during the CH_4_ activation (Fig. 6). These effects collectively reduce the activation barrier by 0.31 eV (0.36 eV for Cu_1_/U6-NO_2_ vs. 0.67 eV for Cu_1_/U6-CH_3_). The quasi in situ EPR result (Fig. 4, A and B) also shows Cu_1_/U6-NO_2_ can facilely prune the H_2_O_2_ to mild ^•^OOH more than excessive ^•^OH species, which then helps to suppress the over-oxidation of oxygenates. Our findings reveal how long-range electronic effects from the NO_2_-BDC ligand propagate through the Zr-oxo nodes to generate electron-deficient active sites capable of concerted methane activation and product desorption, a feat seldom attainable with conventional catalysts. (*12*, *13*, *51*) More importantly, this strategy resolves the long-standing activity-selectivity dilemma in methane hydroxylation, offering a blueprint for addressing similar challenges in other complex catalytic transformations requiring atomic-level control over multiple reaction coordinates.

**Fig. 6.**
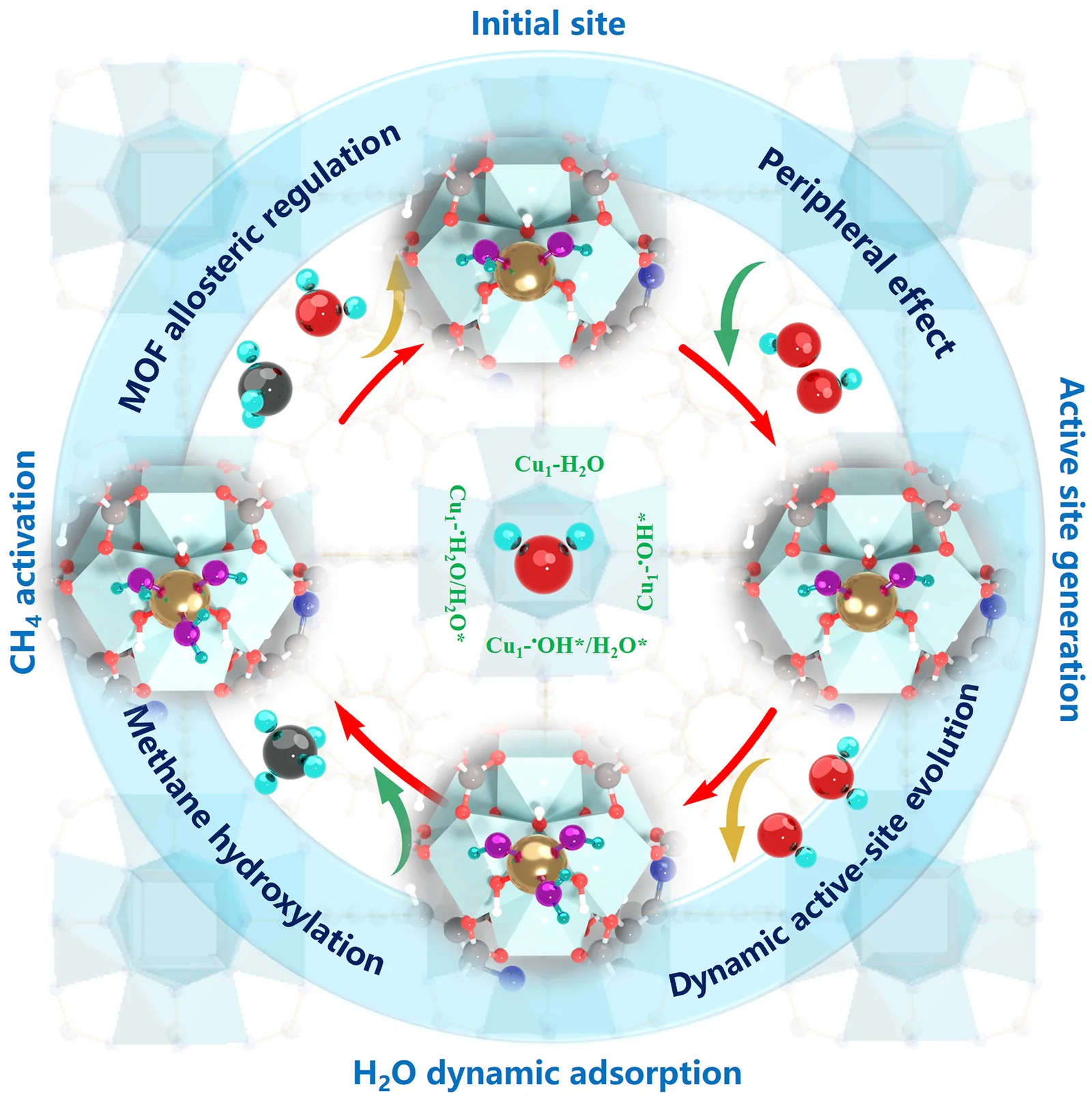
Schematic illustration of the reaction stages and dynamic evolution of the Cu_1_ active site on Cu_1_/U6–X catalysts during methane hydroxylation. The interplay of the MOFs environment and outer coordination dynamically tunes the Cu_1_ site reactivity and selectivity toward oxygenates.

## DISCUSSION

In summary, this work unveils a transformative paradigm for enhancing methane hydroxylation through allosteric control of SACs in MOF architectures. By systematically engineering the outer-shell environment of single Cu atoms anchored at Zr-oxo nodes in U6-X (X = -NO_2_, -H, and -CH_3_), we establish a clear relationship where X-BDC ligand-induced electronic effects can dictate the catalytic performance. Integrated experimental and computational studies reveal that the electron-withdrawing NO_2_-BDC ligand promotes the reactivity of Cu_1_-^•^OH* required for methane activation. These synergistic effects culminate in exceptional catalytic performance, with Cu_1_/U6-NO_2_ achieving a record TOF of 322.4 h^−1^ alongside nearly quantitative selectivity, surpassing most reported SACs. This framework-mediated control mirrors enzymatic allostery, where peripheral NO_2_-BDC ligand remotely optimizes the active site’s electronic and solvation effects through communication via the Zr-oxo nodes. This work establishes outer-shell engineering in MOFs as a general design principle for overcoming activity–selectivity trade-offs in challenging selective oxidation reactions, offering further opportunities for precise control of SACs.

## MATERIALS AND METHODS

### Materials

All chemicals and reagents were obtained from commercial sources and used as received without further purification. Specifically, anhydrous zirconium chloride (ZrCl_4_), copper chloride (CuCl_2_·2H_2_O), N, N-dimethylformamide (DMF), dimethyl sulfoxide (DMSO), methanol, acetone, hydrochloric acid (HCl), sodium hydroxide (NaOH), deuterium oxide (D_2_O), 30 wt.% hydrogen peroxide aqueous solution (H_2_O_2_), 2-methyl-1, 4-benzenedicarboxylic acid (CH_3_-BDC), 1, 4-benzenedicarboxylic acid (H2BDC), 2-Nitro-1, 4-benzenedicarboxylic acid (NO_2_-BDC) were purchased from Sigma. 5,5-dimethyl-1 pyrroline N-oxide (DMPO) was purchased from Dojindo.

### Synthesis of UiO-66-X (−X = -NO_2_, -H, and -CH_3_)

UiO-66-NO_2_ was prepared following a procedure previously reported with slight modifications. (*24*) In a typical synthetic process, 1.64 g of ZrCl_4_ and 1.49 g of NO_2_-BDC were first added to 80 mL DMF. Then, 3.5 mL HCl was subsequently added dropwise into the mixture, which was stirred constantly until all solid precursors completely dissolved. The obtained mixture was transferred to a 200 mL hydrothermal reaction vessel with Teflon liner, which was kept at 120°C for 24 h under static conditions. After cooling to room temperature, the solid MOFs were collected via high-speed centrifugation and thoroughly washed three times with DMF, once with 80°C ultra-pure water, and three times with acetone. Finally, the UiO-66 samples were freeze-dried for 12 h prior to experiments. UiO-66-CH_3_ and UiO-66-H were prepared via a similar method by substituting NO_2_-BDC with CH_3_-BDC and H_2_-BDC, respectively. The resulting UiO-66-X catalysts were named as U6-X.

### Synthesis of Cu_1_/UiO-66-X (−X = -NO_2_, -H, and -CH_3_)

Cu_1_/UiO-66-NO_2_ was prepared following a procedure previously reported with slight modifications. (*28*) In a typical synthetic process, 0.6 g UiO-66-NO_2_ was added to a solution containing 30 mg CuCl_2_·2H_2_O dissolved in 9 mL DMF. The mixture was sonicated for 5 minutes. The vial was sealed and heated in an 85°C isothermal oven for 24 h. The product was collected via high-speed centrifugation and thoroughly washed three times with DMF, once with 80°C ultra-pure water and three times with acetone. Finally, the Cu_1_/UiO-66-X samples were freeze-dried for 12 h prior to experiments. Cu_1_/UiO-66-CH_3_ and Cu_1_/UiO-66-H were prepared via a similar method by substituting UiO-66-NO_2_ with UiO-66-CH_3_ and UiO-66-H, respectively. The catalysts were named as nCu_1_/U6-X, with n as the loading amount of Cu identified by ICP technique.

### Catalytic performance test

The performance test in the oxidation of methane with H_2_O_2_ was carried out in a 40 mL stainless-steel autoclave containing a Teflon liner vessel with a total volume of 30 mL. The vessel was charged with 5 mg catalyst, 15 mL dilute H_2_O_2_ solution (∼1 M). After sealing, the reactor was flushed with 5 bar methane five times to remove the contaminant gases and then charged to 3 MPa methane. The autoclave was heated to 60°C with vigorous stirring at 1200 rpm. The reactions were carried out for 0.25 h, after which the vessel was cooled in an ice-water bath to minimize the volatility of products. After reactions, the gaseous products were analyzed by a gas chromatograph (Agilent 8860) equipped with a PoraBOND Q and a ShinCarbon ST 100/120 columns, as well as a thermal conductivity detector and a flame ionization detector. The liquid product was analyzed by ^1^H nuclear magnetic resonance (NMR) spectroscopy, by mixing 0.5 mL filtered liquid sample with 0.1 mL DMSO/D_2_O solution (1/2000, v/v, DMSO is the internal standard). During NMR measurements, a solvent suppression program was run in order to minimize the signal originated from H_2_O. The amount of CH_3_OOH, HOCH_2_OOH were obtained with the same equation of CH_3_OH. The amount of HCOOH was subtracted with the formic species produced on the pristine catalysts. The oxygenate selectivity (%), oxygenate yields (μmol g_cat_^−1^ h^−1^), turnover frequency (TOF) were calculated using the following equations (Eqs. 1 to 3)

$$\text{Oxygenate selectivity}=\frac{n_{\left(\text{oxygenates}\right)}}{n_{\left(\text{total products}\right)}}\times 100\%$$ (1)

$$\text{Oxygenate yield}=\frac{n_{\left(\text{Oxygenates}\right)}}{m_{\text{cat}.}\times t}$$ (2)

$$\text{TOF}=\frac{n_{\left(\text{oxygenates}\right)}}{n_{\left(Cu_{1}\right)\times t}}$$ (3)

The percentage of methanol product is calculated based on the ratio of CH_3_OOH, and CH_3_OH amongst the liquid oxygenates.

### Catalyst reusability test

In order to study the reusability of the catalyst, the following procedure was carried out. Initially, the catalytic activity of fresh catalyst (30 mg) was tested under the standard conditions as described above. (*9*) Upon completion of the reaction, the reaction solution was filtered, and the solid catalyst was dried overnight by freeze-drying method. The catalytic activity of a part of this dried sample (5 mg) was subsequently tested under standard conditions, representing the second use of the catalyst. The remaining large mass of catalyst was again treated under reaction conditions, and the process repeated until 4 catalytic cycles were obtained.

### Catalyst characterization

Metal loadings in the catalysts were measured by inductively coupled plasma atomic emission spectrometry (ICP-OES) on an IRIS Intrepid II XSP instrument (Thermo Electron Corporation).

BET surface areas of the catalysts were determined by N_2_ adsorption using a Micromeritics ASAP2460–4 apparatus with liquid N_2_ at −196°C. The samples were outgassed at 200°C for 12 h prior to analysis.

X-ray diffraction (XRD) data were collected on a X’pert Pro-1 (PANalytical) diffractometer with a copper anode (Cu *K*α, λ = 1.5432 Å), operating at 40 kV and 40 mA with a scanning angle (2θ) from 5° to 50°.

Attenuated total reflectance infrared (ATR-IR) spectra were recorded on a Thermo Fisher Scientific Nicolet iS50 FTIR Spectrometer (32 scans at a resolution of 4 cm^−1^) with a mercury cadmium telluride detector.

The morphologies of the catalysts were characterized using a Zeiss Ultra 55 Scanning electron microscope (SEM) with an accelerating voltage of 30.0 kV.

The particle size and element distribution of the catalysts were determined on a field emission transmission electron microscope (JEOL JEM-2100F), equipped with a liquid nitrogen cooled energy-dispersive X-ray spectroscopy (EDS) detector for elemental analysis under a working voltage of 200 kV. The AC-HAADF-STEM analysis was performed on a TITAN Themis 300 S/TEM microscope equipped with a probe aberration corrector and monochromator, allowing spatial resolution of 70 pm and energy resolution of 150 meV, a super-X windowless 4 quadrant SDD (silicon drift detector) detection system for STEM-EDX mapping and several annular dark field detectors. The measurements were performed with a spot size of about 500 pm, a semi-convergence angle of 20 mrad, and a probe current of approximately 100 pA. For the HAADF images, collection angles were chosen between 50 and 200 mrad. Additional AC-STEM imaging and elemental analysis were carried out using a JEM-ARM 200F microscope equipped with a cold field-emission gun, a probe spherical aberration corrector, ADF/ABF/BF STEM detectors, an energy filter system (1065), and a high-resolution EDS spectrometer (JED-2300 T) with an ultra-large solid angle of 0.97 sr. The STEM image resolutions of the JEM-ARM 200F at 200, 80, and 60 kV are 0.078, 0.111, and 0.136 nm, respectively.

The dissolution/NMR technique involves the dissolution of the U6-X catalysts in 1 M NaOH (in D_2_O). The ^1^H NMR spectra were acquired on Bruker AVANCE III 300 MHz spectrometer equipped with pulsed field gradient probes.

Thermogravimetric analysis (TGA) was conducted with a TA INSTRUMENTS SDT-Q600 thermal analyzer. The as-prepared UiO-66-X samples were annealed at 300°C to induce dehydroxylation of the nodes and to remove guest molecules in the pores, leaving a material with Zr_6_O_6_L_y_ (L = CH_3_-BDC, H2BDC and NO_2_-BDC; y = 1–6, one ligand connects two Zr-oxo nodes) as a formula (*70*). Therein, one can see that ideal, defect-free (and dehydroxylated) UiO-66-X has a composition of Zr_6_O_6_(L)_6_, where the BDC/Zr-oxo ratio is 6 (i.e., 6 × 2 = 12 carboxylate groups are coordinated to each Zr-oxo node) (*71*). According to the molecular weight difference between the ideal Zr_6_O_6_L_6_ structure and the ZrO_2_ residual, the ideal plateau should individually reach at 234%, 220% and 257%, respectively. In fact, TGA results showed that the experimental plateau was 207%, 203% and 243%, corresponding to the L/Zr-oxo ratios of 5.3, 5.3, and 5.5 in all UiO-66-X materials. The charge imbalance created by the ligand deficiencies in UiO-66-X could be compensated by OH^−^/OH_2_ pairs under atmospheric conditions.

Raman spectra were recorded on a Senterra Via Raman microscope spectrometer. A diode laser emitting 785 nm was used and the Raman spectrum was collected in the range of 2000–100 cm^−1^. The spectra were collected at 50 mW Laser power with 120 acquisitions of 60 s each.

The X-ray photoelectron spectroscopy (XPS) was performed in a Kratos Axis Ultra DLD photoelectron spectrometer using monochromatic Al Ka (1486.7 eV) X-ray irradiation. High-resolution spectra were collected using an analysis area of ≈ 300 μm × 700 μm, and a 20 eV pass energy. The binding energy of the photoemission spectra was calibrated to the C *1s* peak with a binding energy of 284.8 eV. The results were deconvoluted using XPS PEAK41 software. The % Lorentzian-Gaussian (L/G) which indicated the fitting degree of peaks was fixed to 20% during deconvolution.

The near-ambient pressure X-ray photoelectron spectroscopy (NAP-XPS) measurements were carried out using a SPECS/NAP-XPS system equipped with a monochromatic Al Kα source (*h*ν = 1486.6 eV). The sample was first pretreated under vacuum (∼10^−9^ mbar) at room temperature to remove residual species physically adsorbed in the MOF pores. After baseline stabilization, a Cu *2p* spectrum was collected under vacuum as the reference state. Subsequently, water vapor was introduced into the analysis chamber at a partial pressure of 0.156 mbar. The sample was allowed to equilibrate under this water vapor environment, and a second Cu *2p* spectrum was acquired to probe the influence of water adsorption. Finally, the system was gradually heated to around 100°C under dynamic vacuum to remove adsorbed water molecules. After thermal treatment and pressure stabilization, a third Cu *2p* spectrum was recorded. This sequence enabled the tracking of Cu chemical state evolution during exposure to and removal of water vapor, providing insight into the interaction between Cu active sites and H_2_O under near-realistic conditions.

The X-ray absorption spectroscopy (XAFS) study was performed at the BL08B2 of SPring-8 (8 GeV, 100 mA), Japan. The X-ray beam was mono-chromatized with water-cooled Si (311) double-crystal monochromator and focused with two Rh coated focusing mirrors with a beam size of 2.0 mm in the horizontal direction and 0.5 mm in the vertical direction around sample position. To obtain X-ray absorption fine structure (XAFS) spectra both in near and extended edge of Cu foil and CuO samples were used as references. The catalysts and standard samples were measured with fluorescence and transmission modes, respectively. The spectra were analyzed and fitted using an analysis program Demeter 2 (*72*).

Quasi in situ electron paramagnetic resonance (quasi in situ EPR) spectroscopic measurements were obtained at room temperature using a Bruker A200 EPR spectrometer operated at X-band frequency using DMPO as spin-trapping agent for free radical. The EPR characterization details in our manuscript are as follows: 0.1 mL radical capture agent of DMPO aqueous solution (28 mg mL^−1^) was pre-added into a 2 mL brown flask. The reaction solution (0.1 mL) was collected into the brown flask from the vessel and stirred for 3 min to fully capture the ^•^OH species. Then, the mixture was filtered and frozen by liquid N_2_. These samples were thawed subsequently for the EPR measurements. For the EPR spectra measurement of ^•^OOH (^•^O_2_^−^) species, the procedure is similar to that used for the ^•^OH trapping experiments except that water is replaced with DMPO methanol solution (5 mg mL^−1^) during the reaction. The parameters for EPR measurements were as follows: microwave frequency of 9.3 GHz, microwave power of 10 mW, and modulation frequency of 100 kHz.

The in situ diffuse reflectance infrared Fourier transform spectroscopy (in situ DRIFTS) experiments were conducted on a Bruker VERTEX 70 instrument equipped with an in-situ gas system and operated over the range of 900–1700 cm^−1^ at a resolution of 4 cm^−1^. For comparison of adsorbed species on Cu_1_/U6-X under reaction conditions, the catalyst samples were pretreated at 150°C for 1 h and then cooled to 60°C under He. A background spectrum was collected as reference prior to the introduction of CH_4_ gas or H_2_O_2_ into the chamber. The CH_4_ gas was introduced into the reaction cell simultaneously through bubbling the H_2_O_2_ solution. After the chamber was flushed with He, DRIFTS spectra over Cu_1_/U6-X catalysts were recorded. In addition, CH_4_ instead of H_2_O_2_ and CH_4_ was used in the control experiment for studying the role of H_2_O_2_ and CH_4_ in the production of carbonous species.

### Computational details

All spin-polarized density functional theory (DFT) calculations were performed in the Vienna Ab initio Simulation Package (VASP) (*73*) with the Perdew–Burke–Ernzerhof (PBE) exchange–correlation functional within the generalized gradient approximation (GGA) (*74*, *75*) The projector augmented wave (PAW) method was employed to describe the core and valence electrons and a plane-wave cut-off energy of 450 eV was used. (*76*–*78*) Dispersion corrections were included via the DFT-D3 method. (*79*) To account for strong electron correlation in Zr atoms, the DFT + U approach was applied with *U* = 4 eV and *J* = 1 eV. (*80*)

A primitive cell of defective UiO-66-X was chosen as the catalyst model, with optimized lattice parameters α = β = γ = 60° and a = b = c = 14.68 Å. The explicit solvent effect was incorporated with 38 water molecules for Cu_1_/U6-X (X = -CH_3_ and -NO_2_). Cu single atoms were anchored via two -OH groups on the Zr-oxo nodes. The Brillouin zone was sampled using Gamma-centered Monkhorst–Pack k-point grids (*81*). Geometry optimizations were considered converged when forces on all atoms were below 0.05 eV/Å and the energy change between ionic steps was less than 10^−5^ eV.

Ab initio molecular dynamics (AIMD) simulations were conducted in the NVT ensemble at 333.15 K using a Nosé–Hoover thermostat. A 10 ps equilibration run was performed to establish a stable water environment around Cu_1_/U6-X, followed by another 10 ps trajectory to study CH_4_ adsorption near the Cu_1_ site, with a timestep of 1 fs. Bader charge analysis was carried out on structures extracted every 100 fs from the AIMD trajectories to evaluate charge states of Cu_1_ and relevant O atoms. (*66*)

Kinetic free energy barriers were computed using the slow growth method. (*67*) The collective variable (CV), ξ, was linearly altered from the initial state (IS) to the final state (FS) with a specific velocity $\left(\dot{\xi }\right)$ about 8 × 10^−4^ Å per simulation step ($\partial \dot{\xi }$). The work performed during the transformation is given by

$$W_{\text{IS}\to \text{FS}}=\int_{\xi \left(\text{IS}\right)}^{\xi \left(\text{FS}\right)}\left(\frac{\partial F}{\partial \xi }\right)\cdot \dot{\xi }\mathrm{dt}$$ (4)

where *F* is the free energy as a function of the general coordinate *q*, which evolves with time *t*. In the limit of infinitesimally slow switching, $W_{\text{IS}\to \text{FS}}$ corresponds to the free energy change between IS and FS.

For active site generation, the CV was defined as the difference between the O-H bond length in Cu_1_-H_2_O* and the distance between the H atom in Cu_1_-H_2_O* and O atom in H_2_O_2_. For CH_4_ activation, the CV was defined as the difference between the breaking C-H bond length and the distance between the transferring H atom and the O atom in Cu_1_-^•^OH*.
