# Supplementary material for: Remote allosteric control of single-atom Cu sites in Zr-MOFs enables selective CH4 hydroxylation
Source: Sci Adv. 2026 Oct 14;12(42):eaei9401. doi: 10.1126/sciadv.aei9401 (PMC13665999; doi:10.1126/sciadv.aei9401)
Supplement: Supplementary file 1 [file sciadv.aei9401_sm.pdf]

Science Advances
AAAS

Supplementary Materials for

Remote allosteric control of single-atom Cu sites in Zr-MOFs enables selective CH_4 hydroxylation

Geqian Fang *et al.*

Corresponding author: Sen Lin, slin@fzu.edu.cn; Jian Lin, jianlin@dicp.ac.cn;
Andrei Y Khodakov, andrei.khodakov@univ-lille.fr

Sci. Adv. **12**, eaei9401 (2026)
DOI: 10.1126/sciadv.aei9401

The PDF file includes:

Figs. S1 to S39
Tables S1 to S9
Legends for movies S1 to S4

Other Supplementary Material for this manuscript includes the following:

Movies S1 to S4

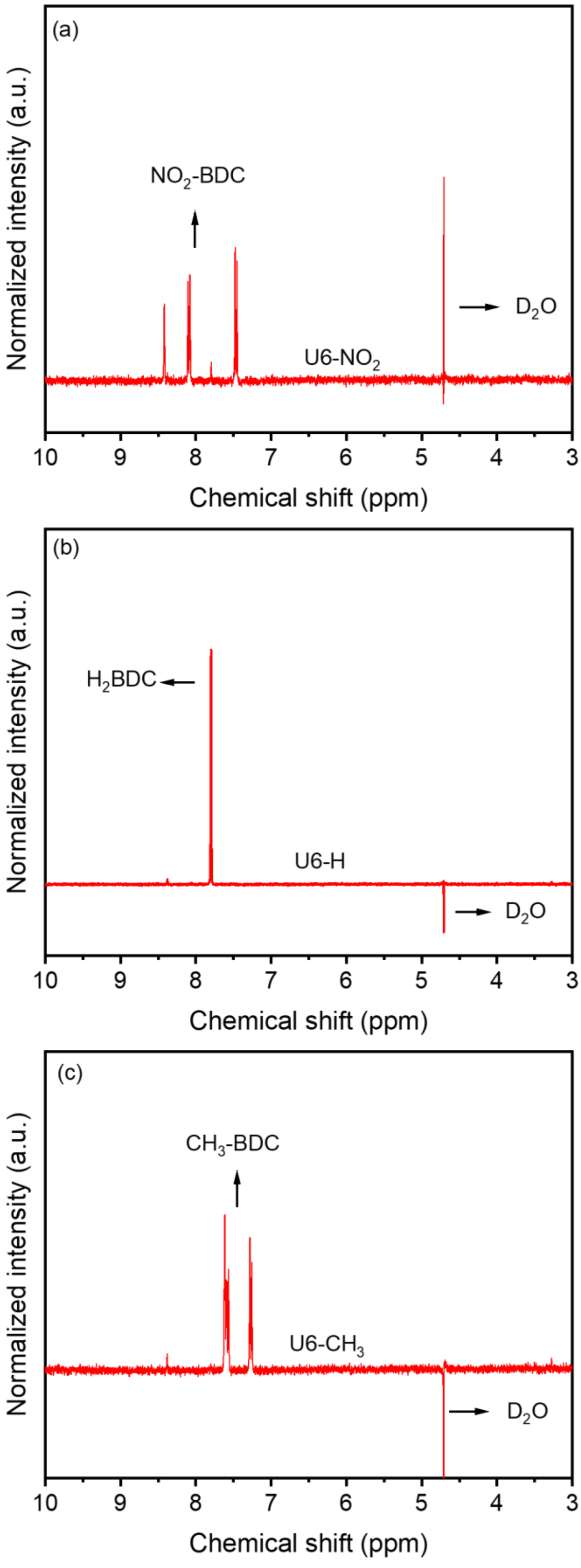

fig. S1. Solution state ^{1}H NMR of (A) U6-NO_2, (B) U6-H, and (C) U6-CH_3 after dissolution in 1 M NaOH/D_2O.

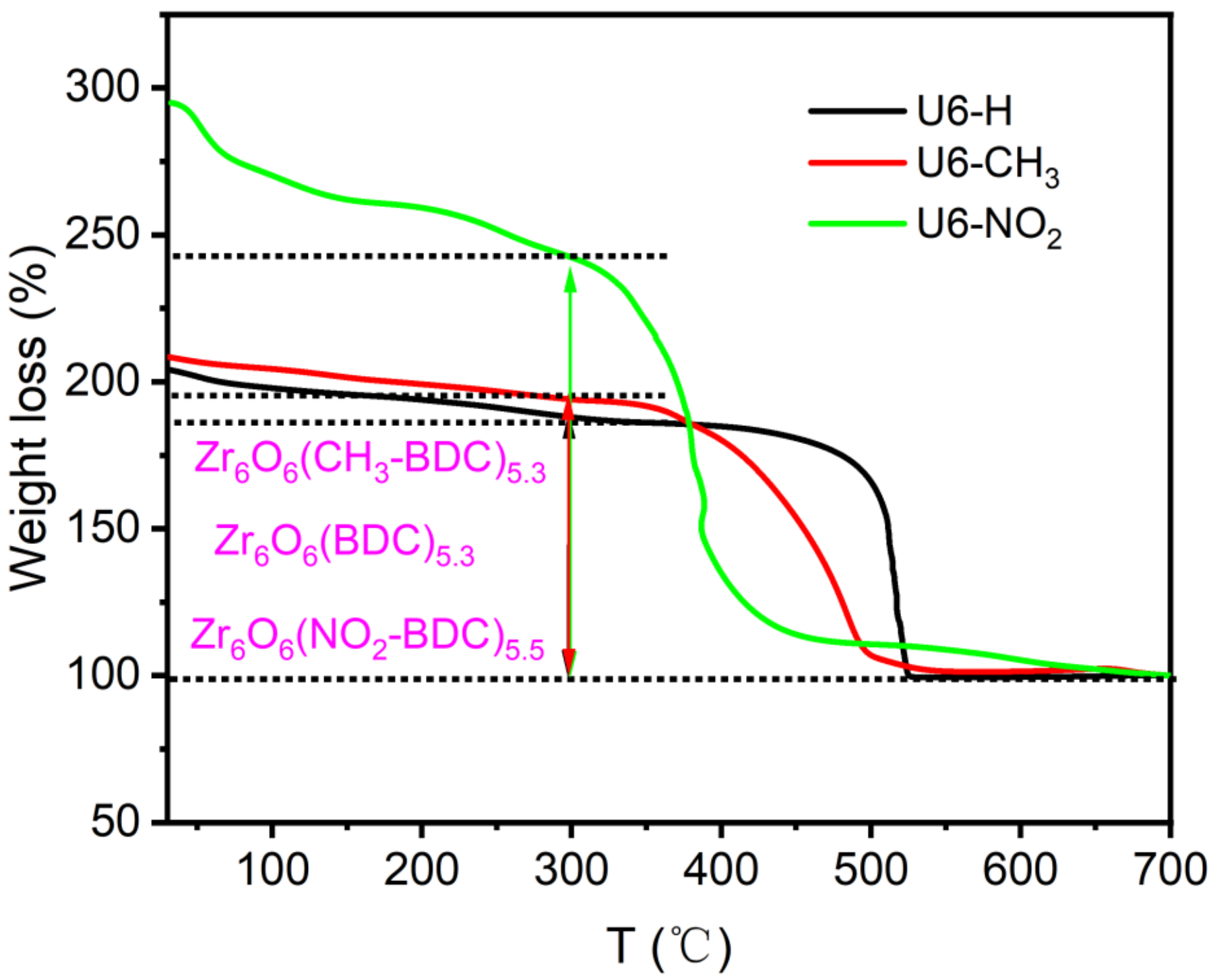

fig. S2. TGA analysis of U6-X.

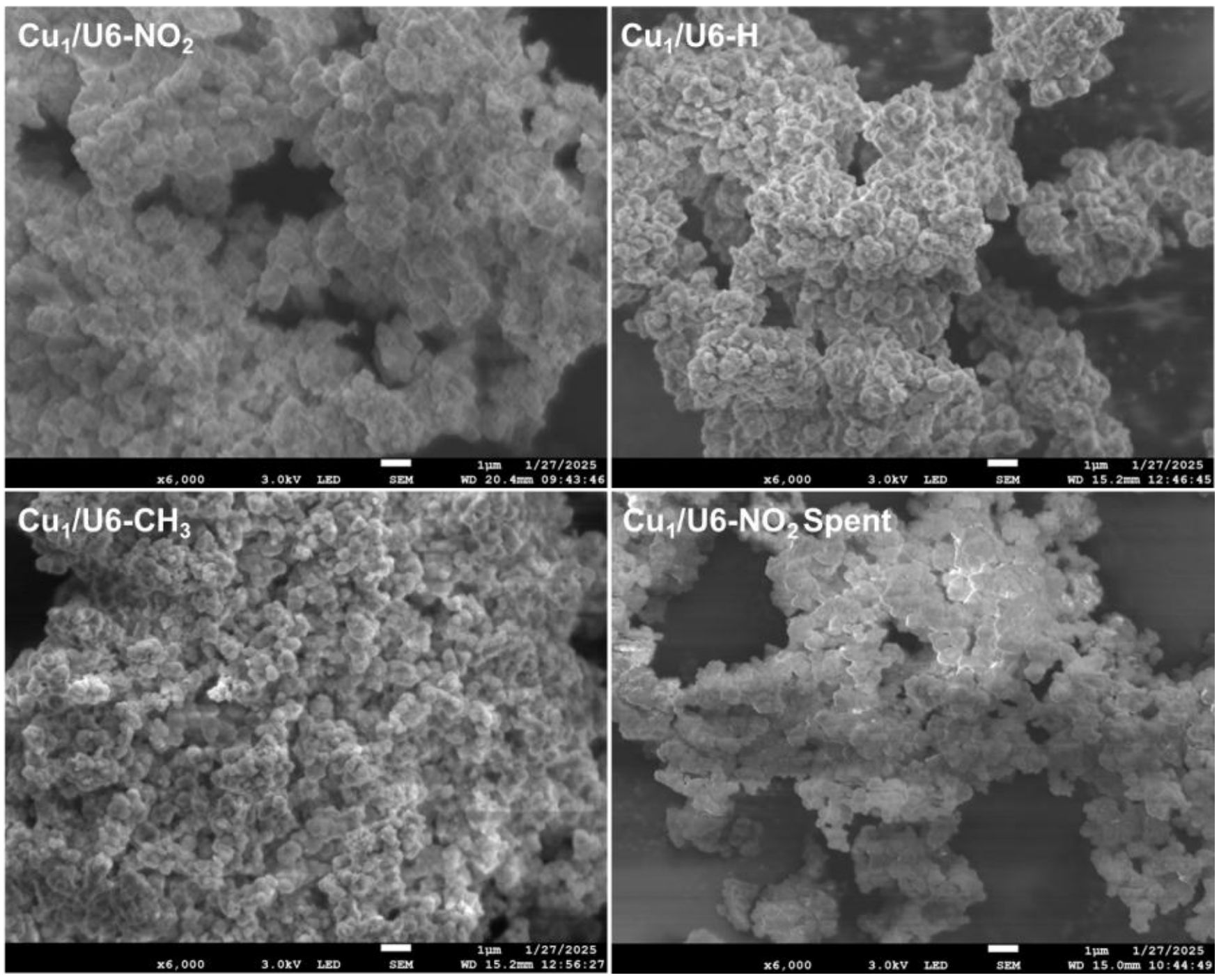

fig. S3. Representative SEM images of different Cu_1/U6-X catalysts.

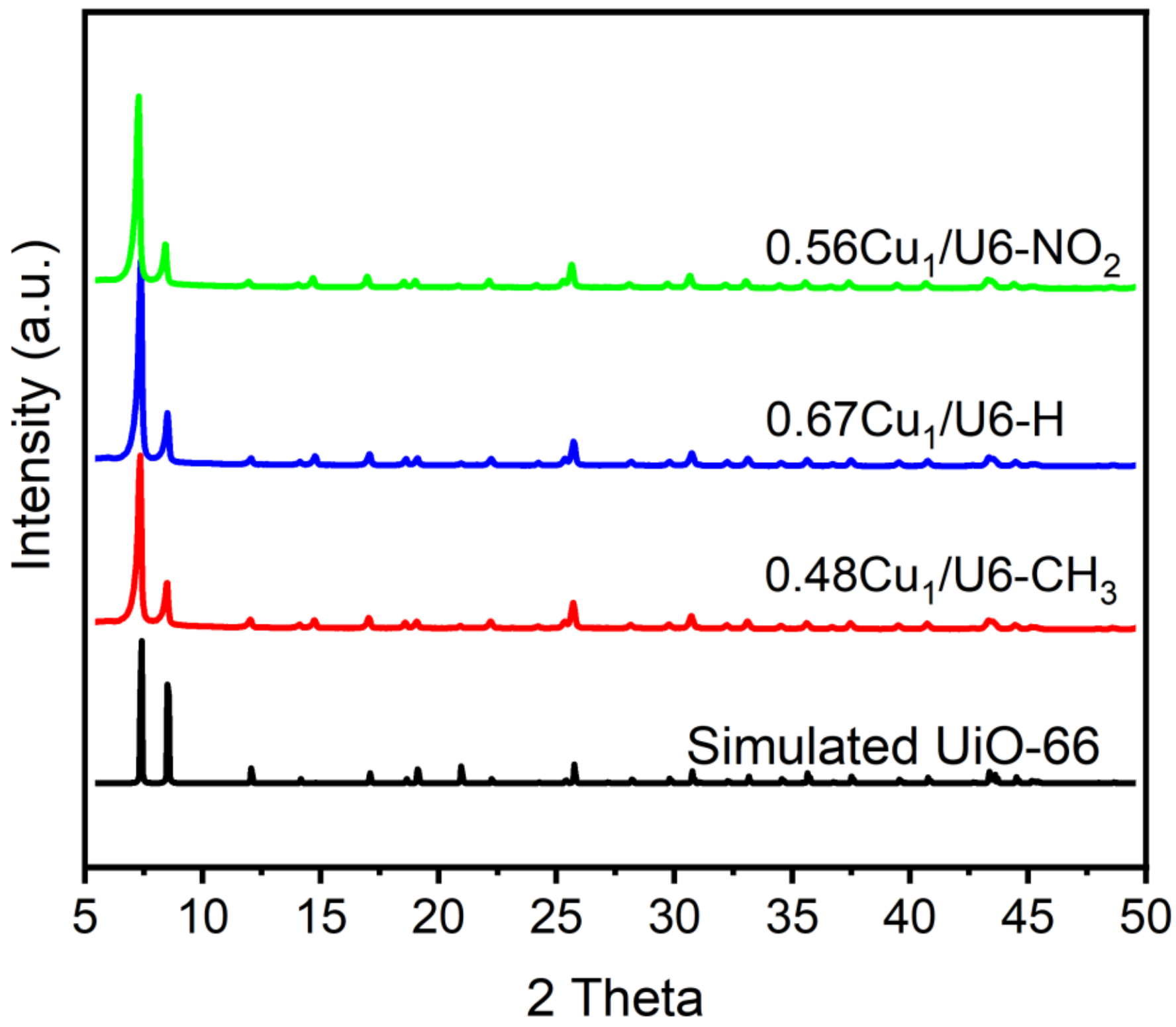

fig. S4. XRD patterns of different Cu_1/U6-X catalysts.

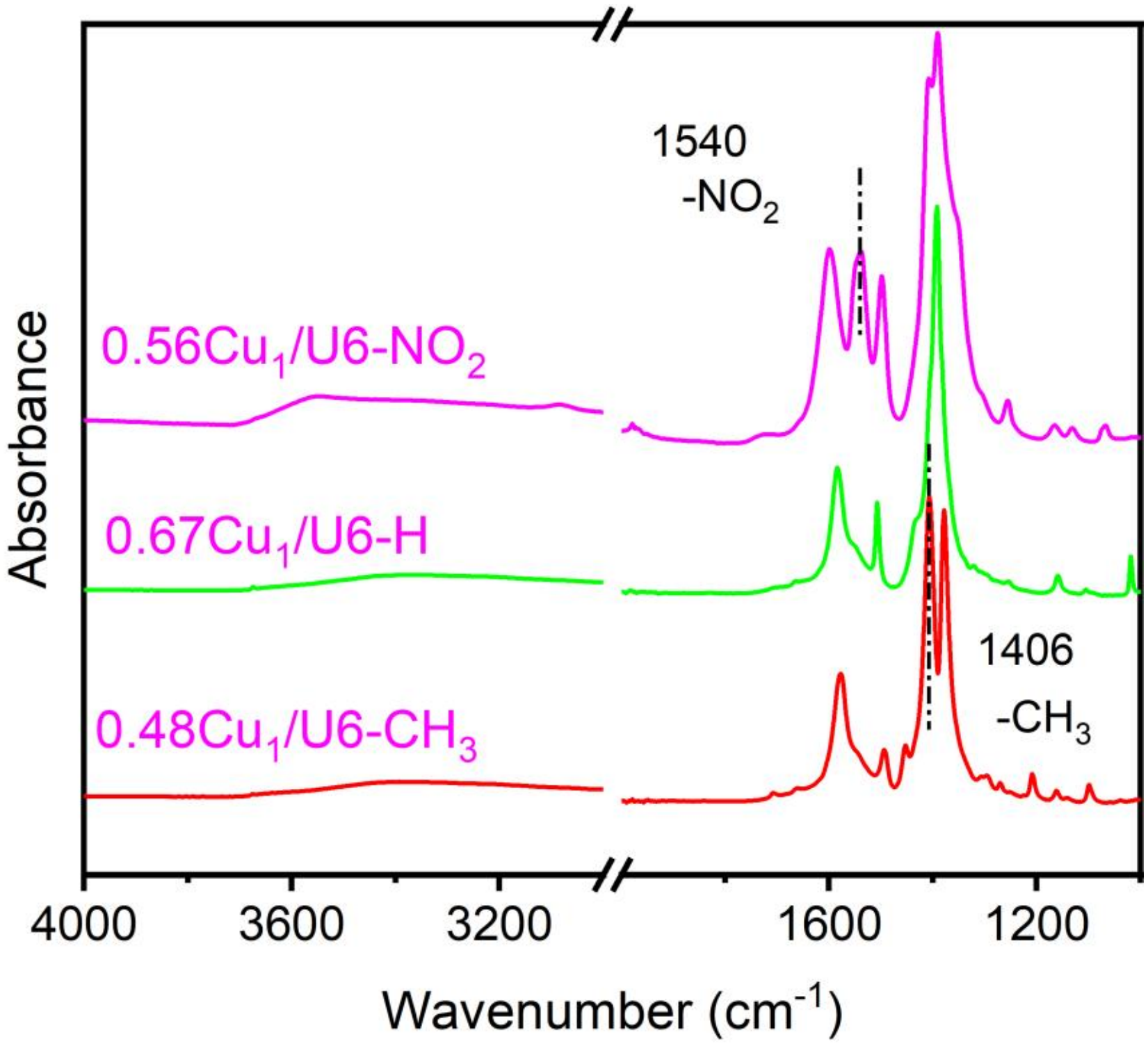

fig. S5. ATR-IR spectra of different Cu_1/U6-X catalysts.

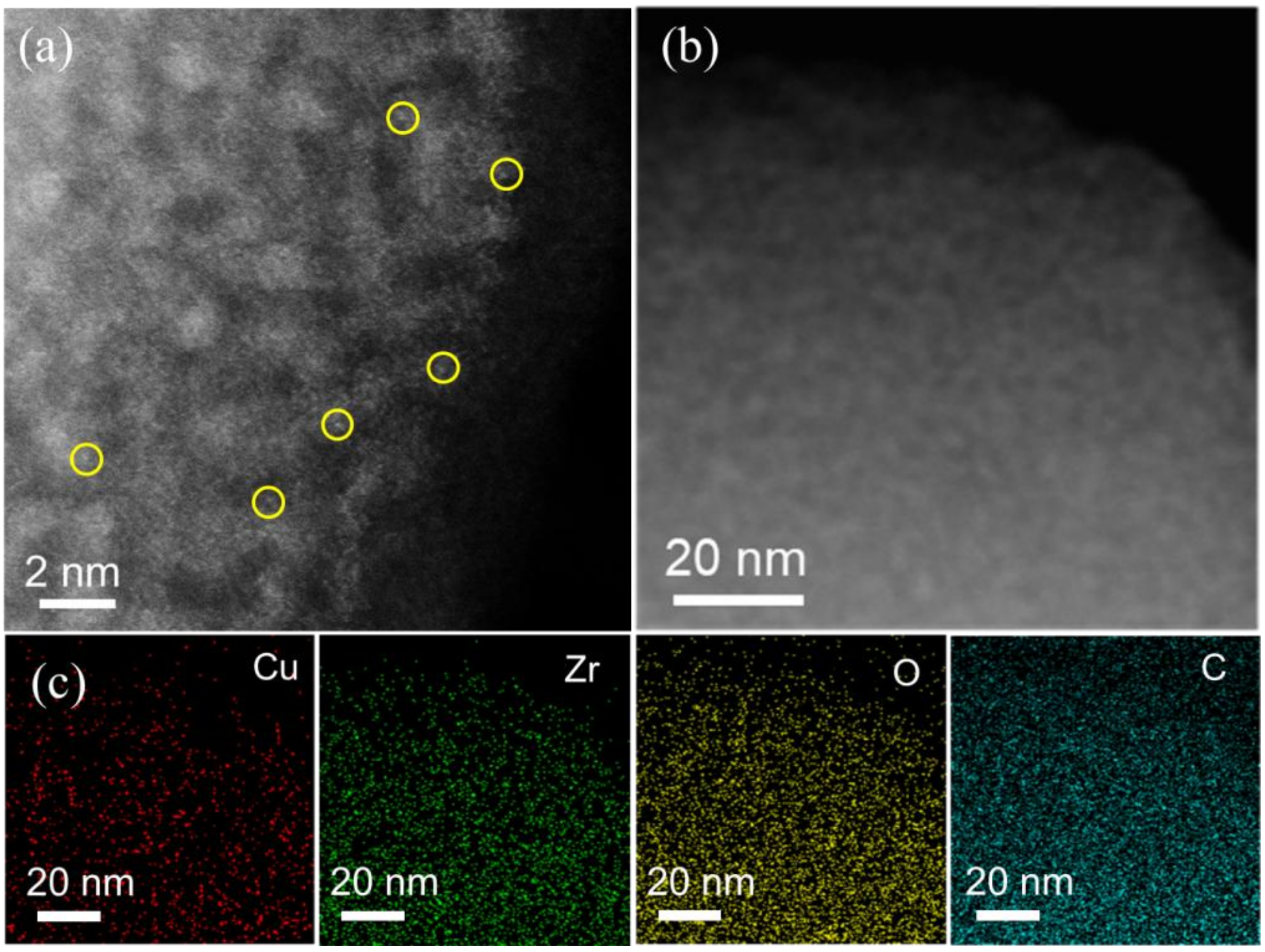

fig. S6. (A), (B) Representative AC-HAADF-STEM images with (C) corresponding EDS elemental mappings for 0.67Cu_1/U6-H, catalysts. Possible Cu_1 species are indicated by yellow circles.

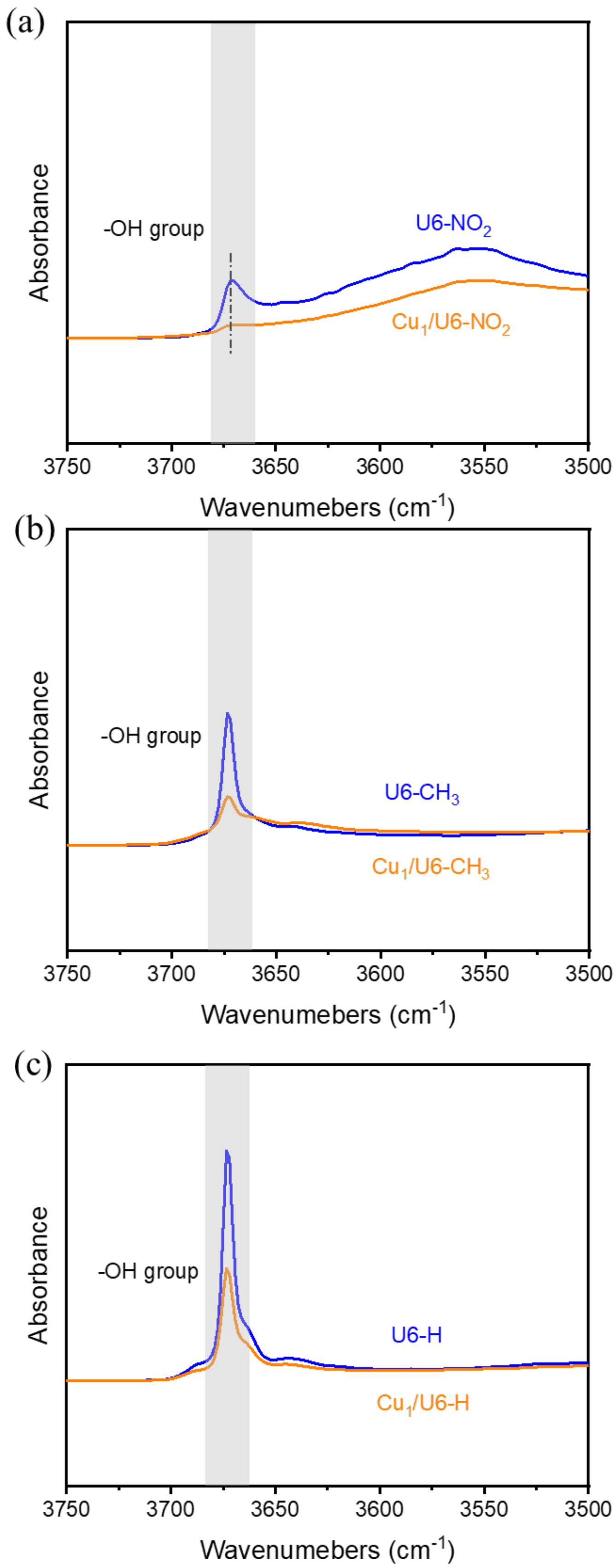

fig. S7. DRIFTS spectra of (A) $0.56Cu_1/U6\text{-}NO_2$ vs. $U6\text{-}NO_2$, (B) $0.67Cu_1/U6\text{-}H$ vs. U6-H, and (C) $0.48Cu_1/U6\text{-}CH_3$ vs. $U6\text{-}CH_3$.

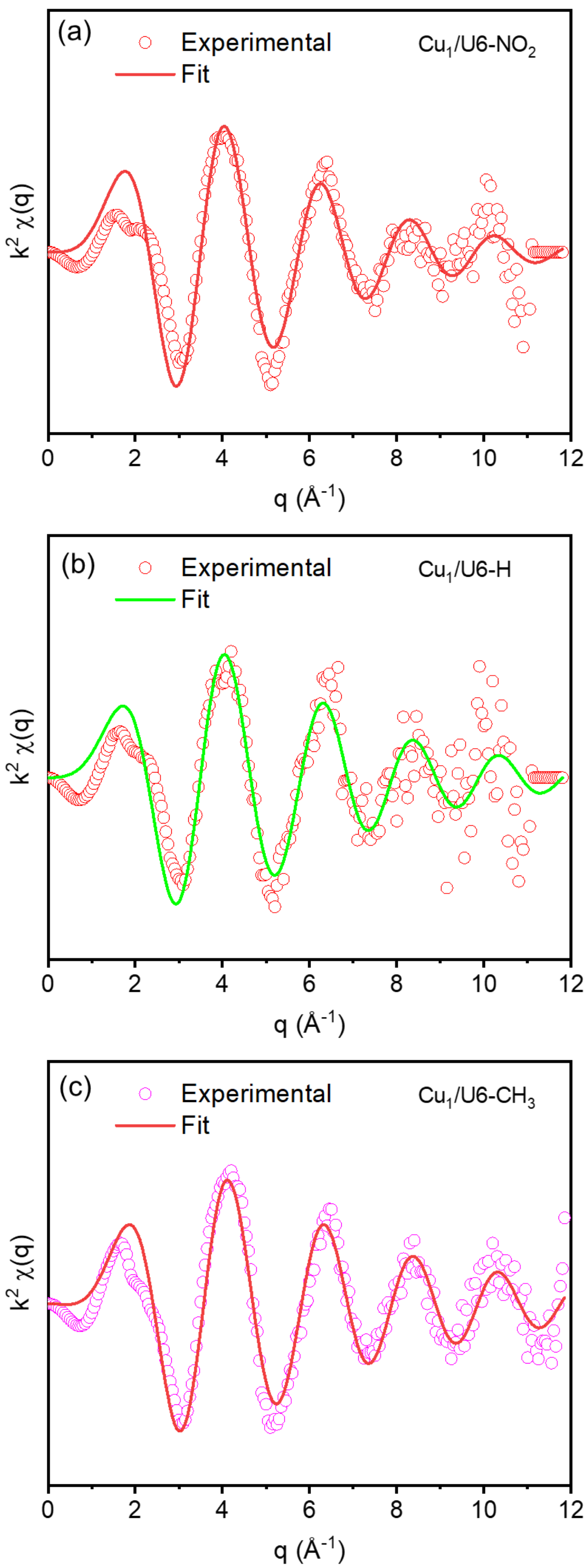

fig. S8. ***K*** **space fitting results of the EXAFS spectra for (A) 0.56Cu_1/U6-NO_2, (B) 0.67Cu_1/U6-H, and (C) 0.48Cu_1/U6-CH_3.**

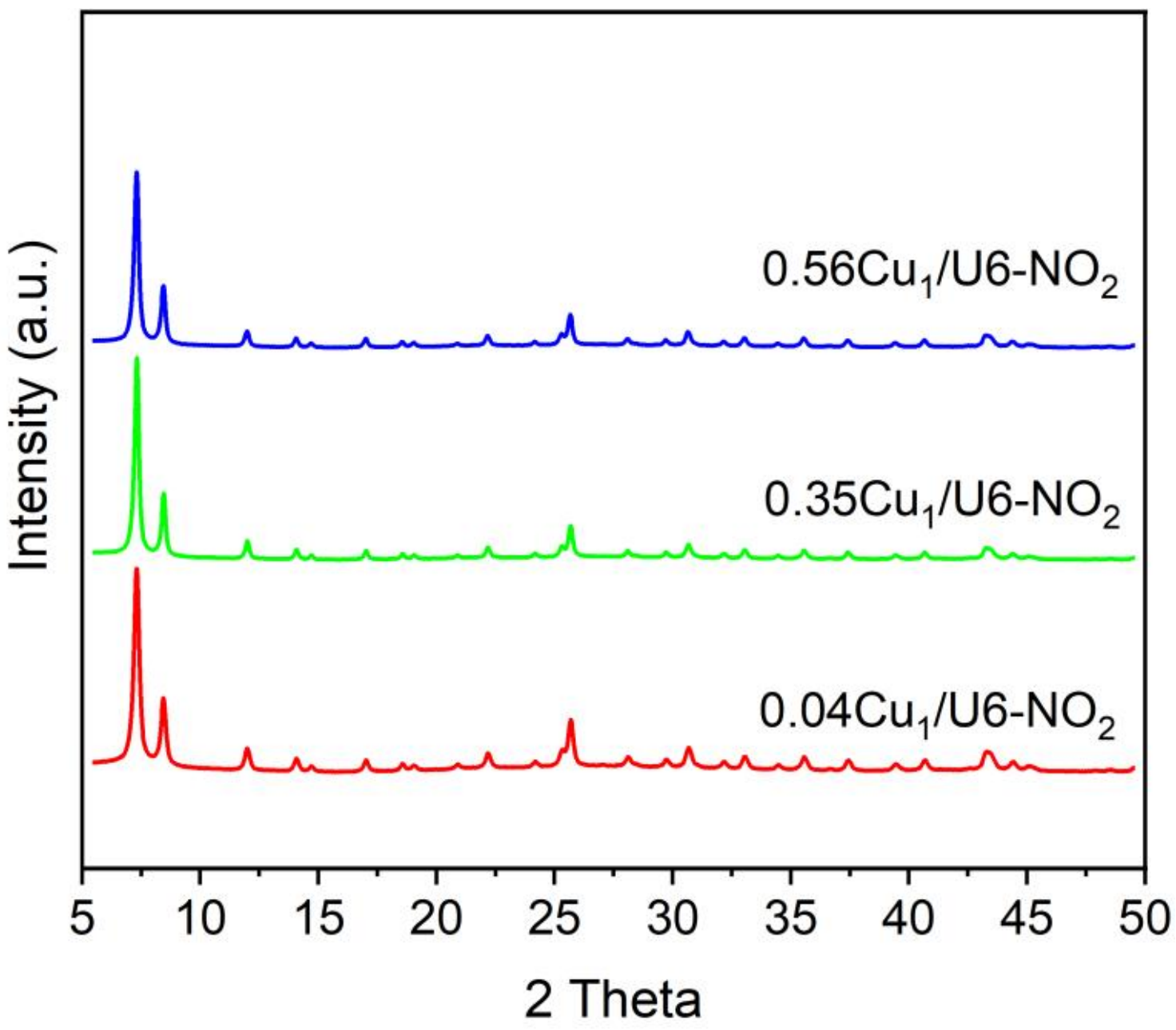

fig. S9. **XRD patterns of different Cu_1/U6-NO_2 catalysts**

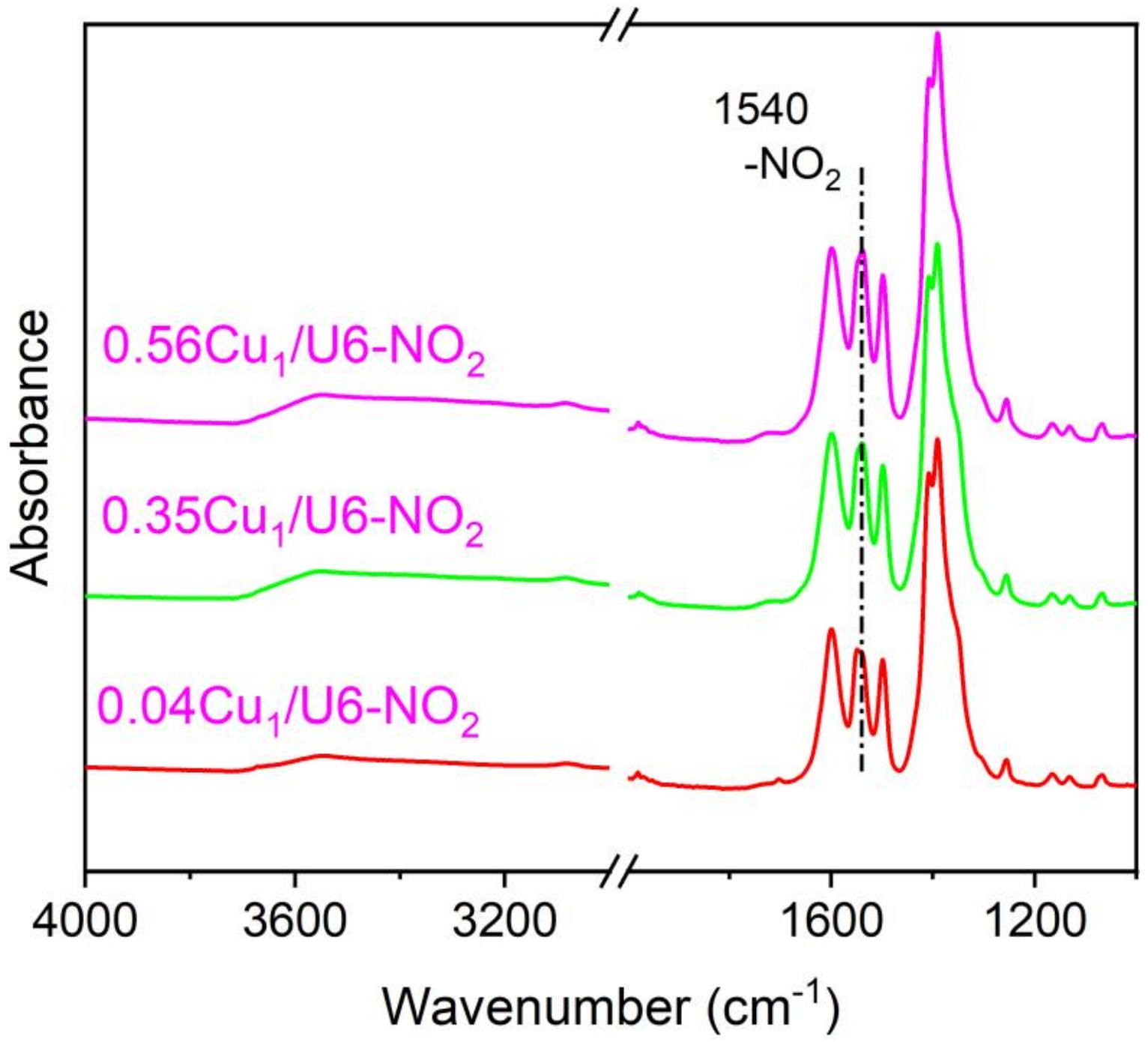

fig. S10. **ATR-IR spectra of different $Cu_1/U6\text{-}NO_2$ catalysts.**

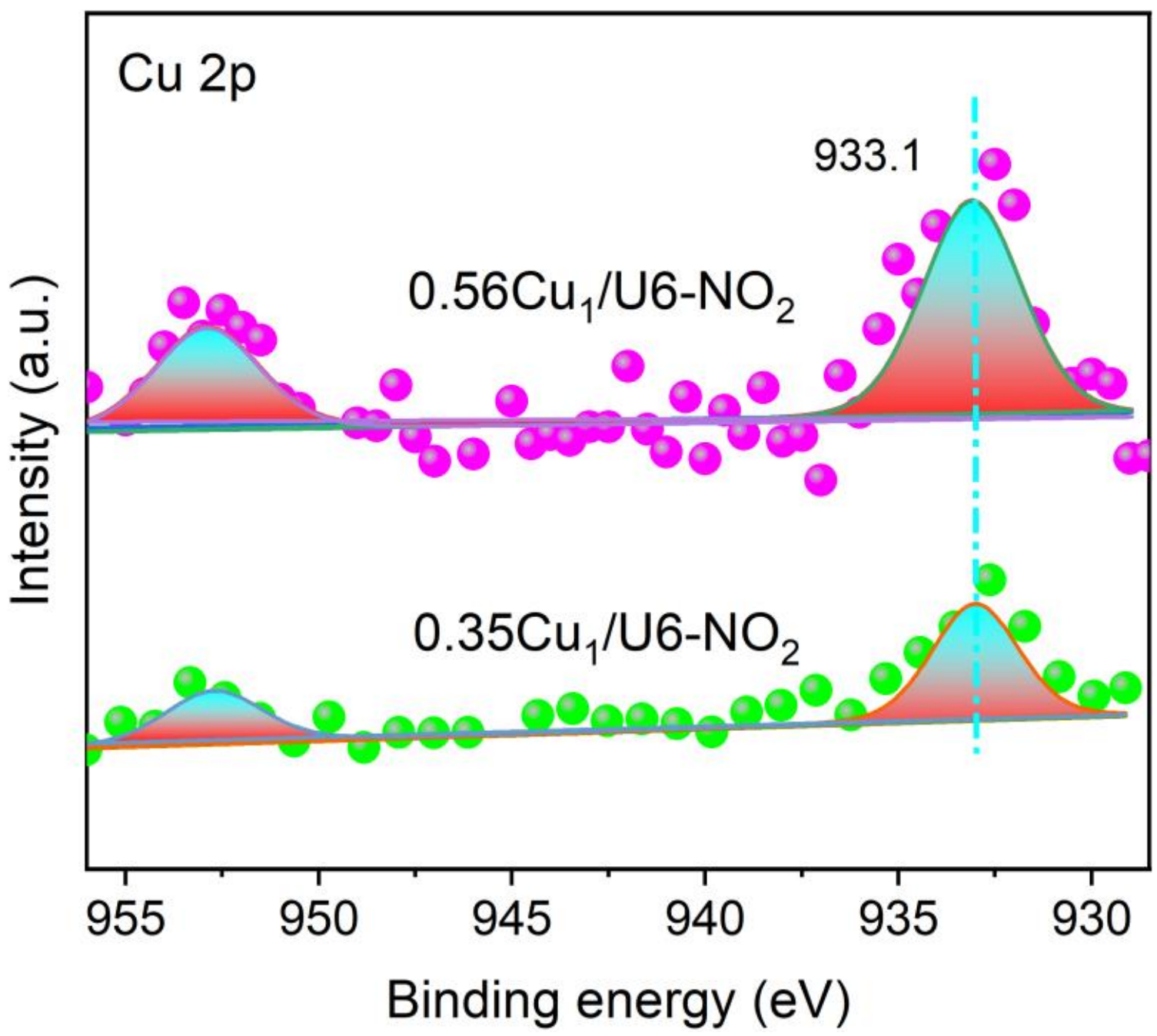

fig. S11. **XPS spectra of Cu 2p over different $Cu_1/U6\text{-}NO_2$ catalysts.**

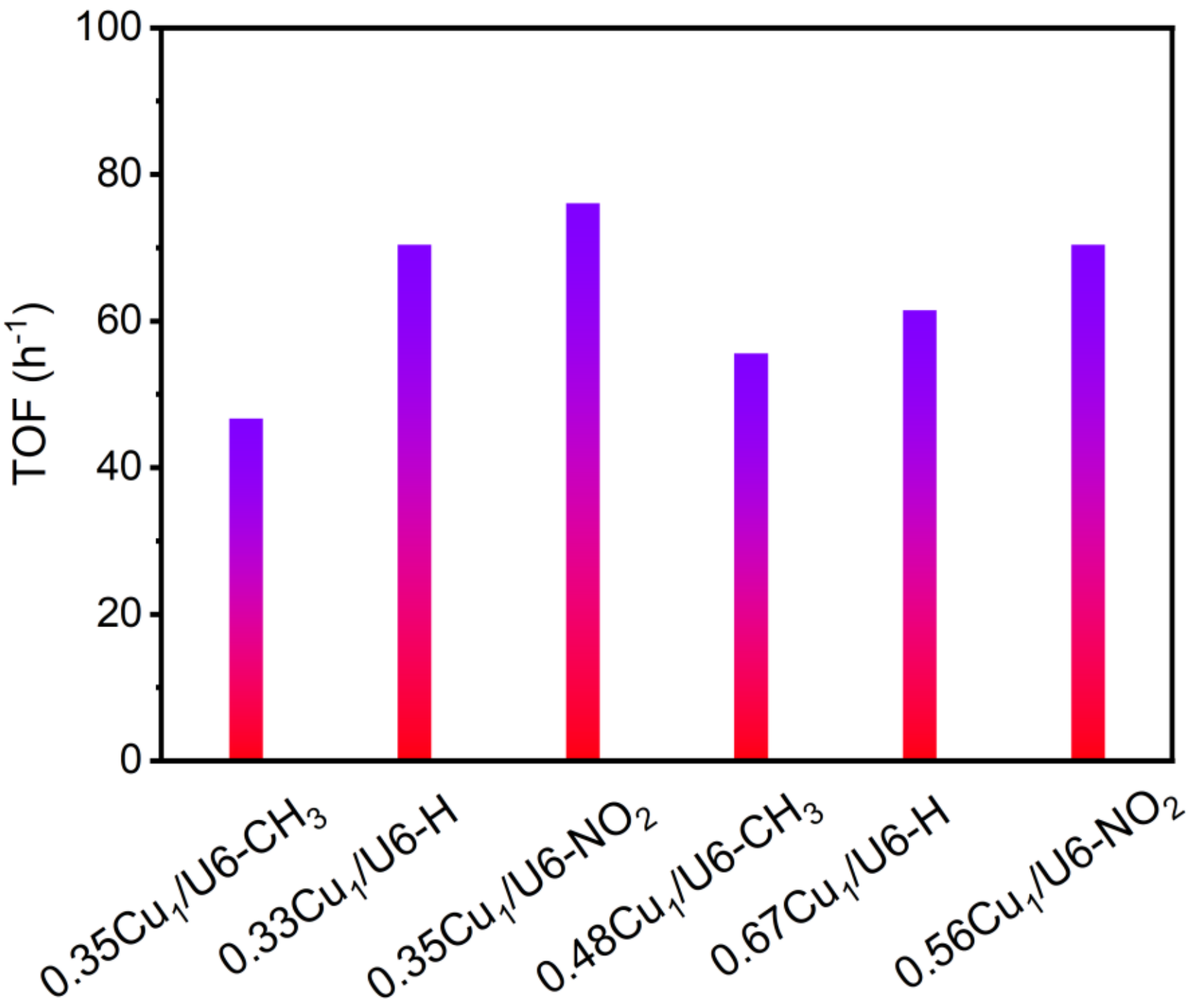

fig. S12. The TOF in DSOM reaction over Cu_1/U6-X catalysts.

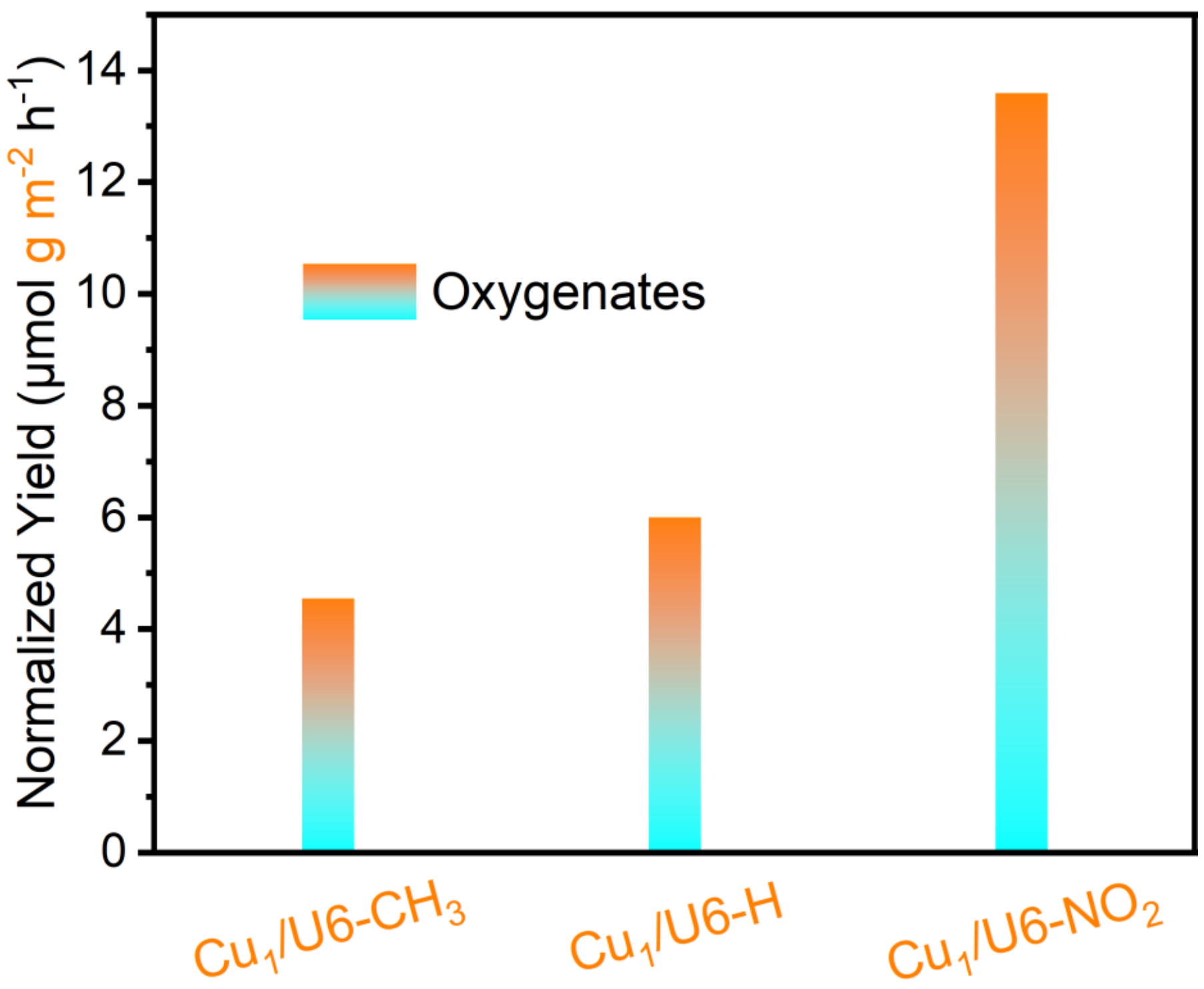

fig. S13. Catalytic activity normalized to the surface areas on 0.05Cu_1/U6-CH_3, 0.05Cu_1/U6-H and 0.04Cu_1/U6-NO_2 catalysts.

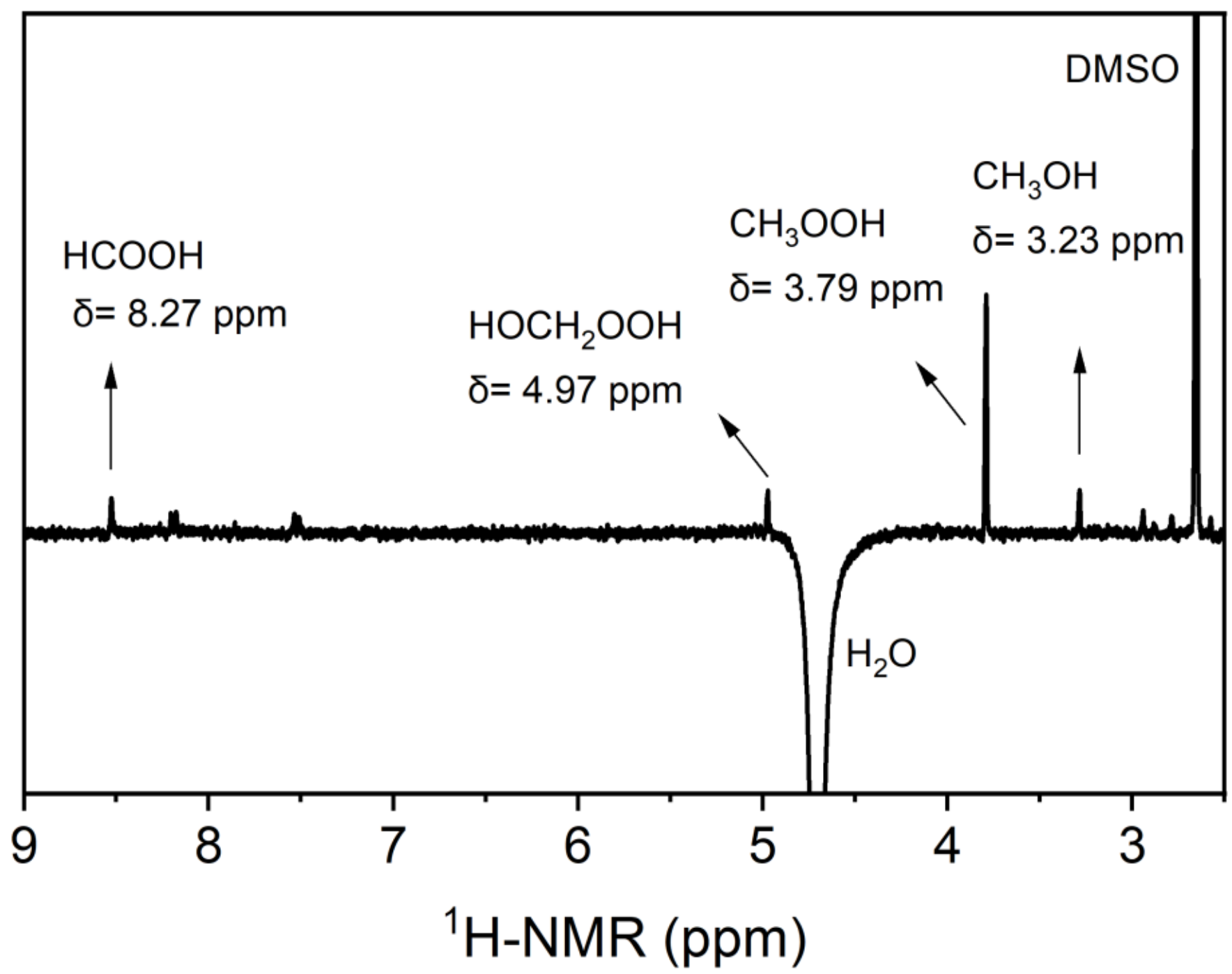

fig. S14. ^{1}H NMR spectrum of liquid products over Cu_1/U6-NO_2. Test conditions: Pressure (CH_4) = 3 MPa, Catalyst: 5 mg 0.04Cu_1/U6-NO_2, 1 M H_2O_2, 60 ºC, reaction for 4 h.

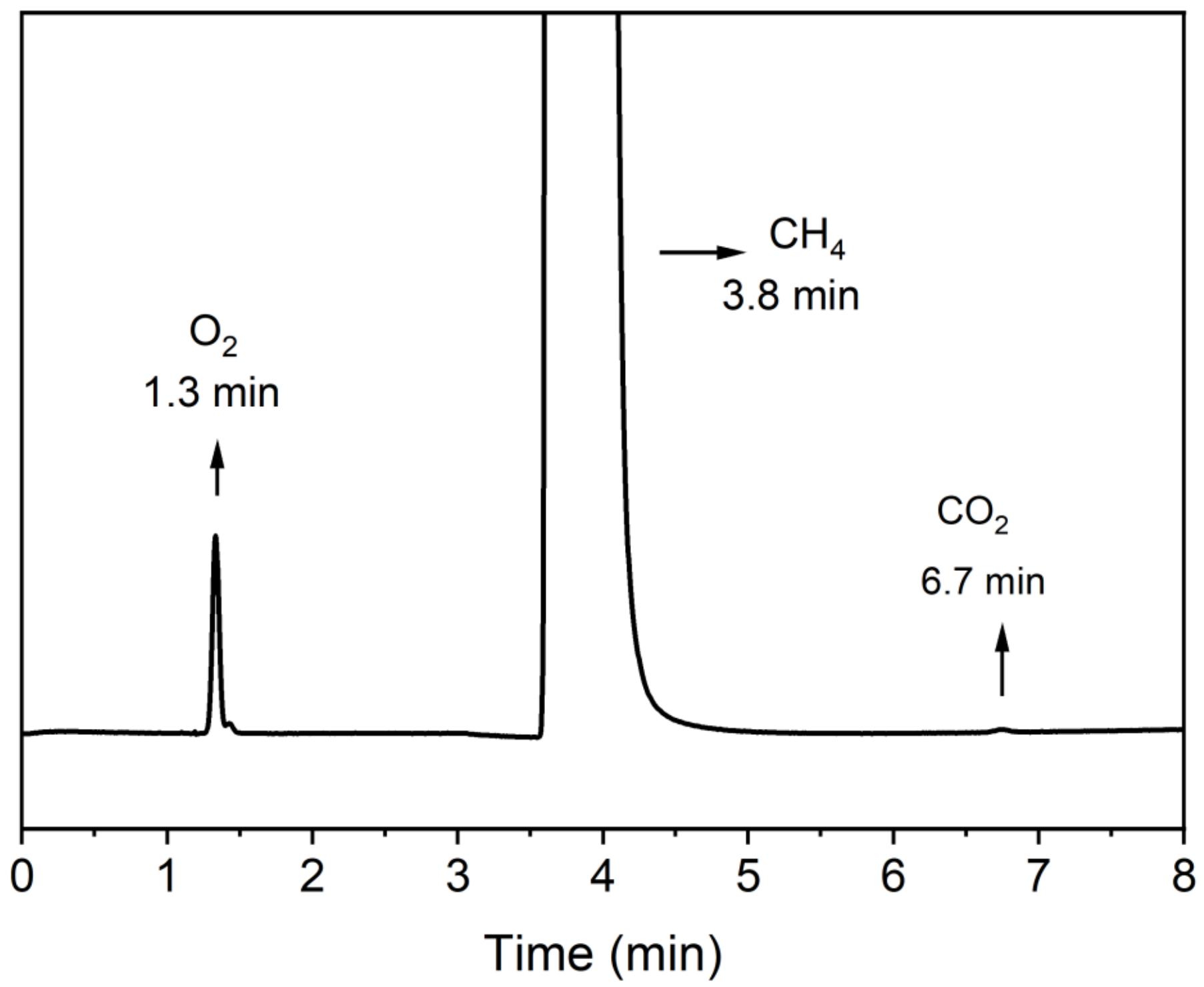

fig. S15. The TCD spectra of gaseous products over Cu_1/U6-NO_2. Test conditions: Pressure (CH_4) = 3 MPa, Catalyst: 5 mg 0.04Cu_1/U6-NO_2, 1 M H_2O_2, 60 ºC, reaction for 4 h.

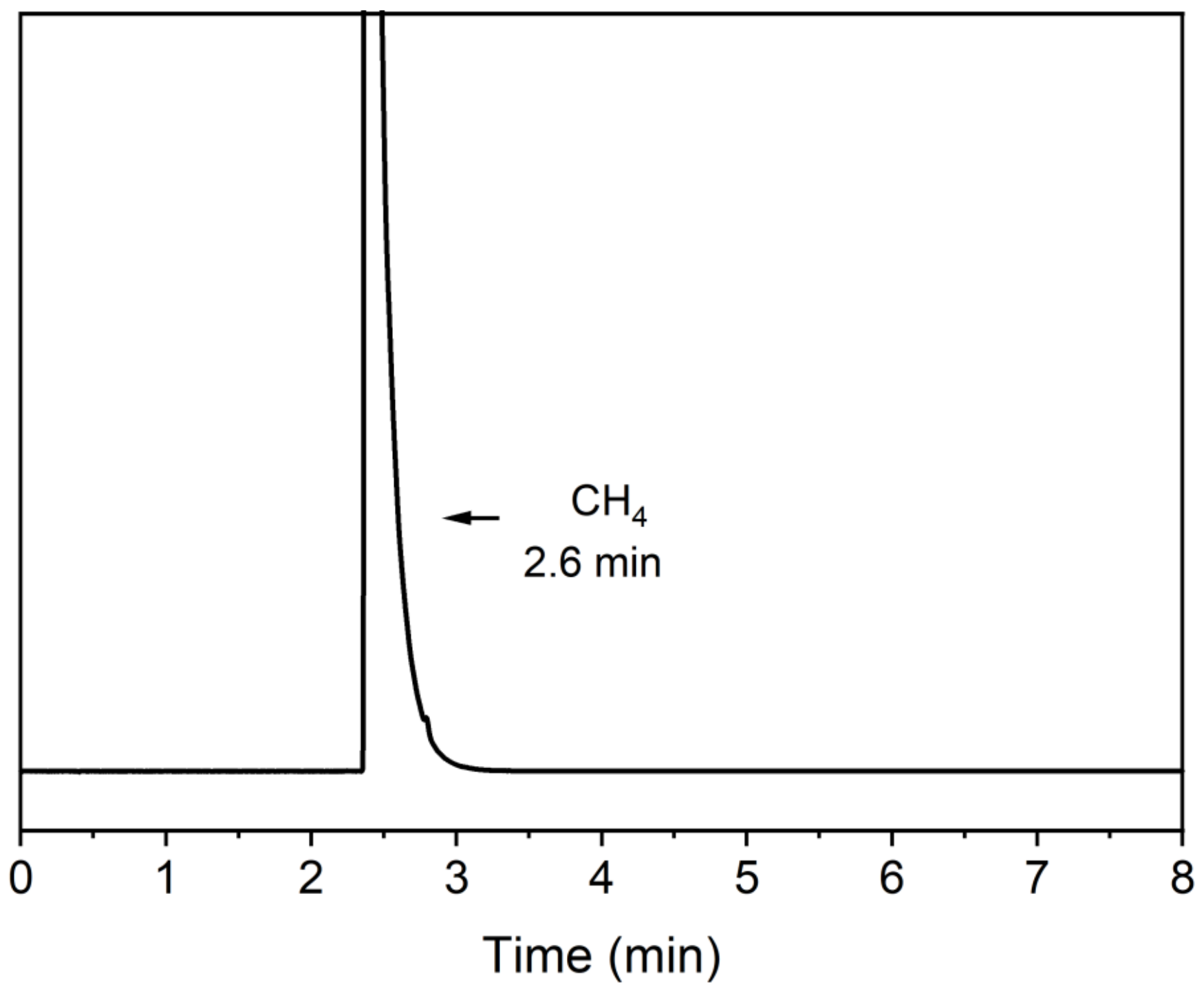

fig. S16. The FID spectra of gaseous products over Cu_1/U6-NO_2. Test conditions: Pressure (CH_4) = 3 MPa, Catalyst: 5 mg 0.04Cu_1/U6-NO_2, 1 M H_2O_2, 60 ºC, reaction for 4 h.

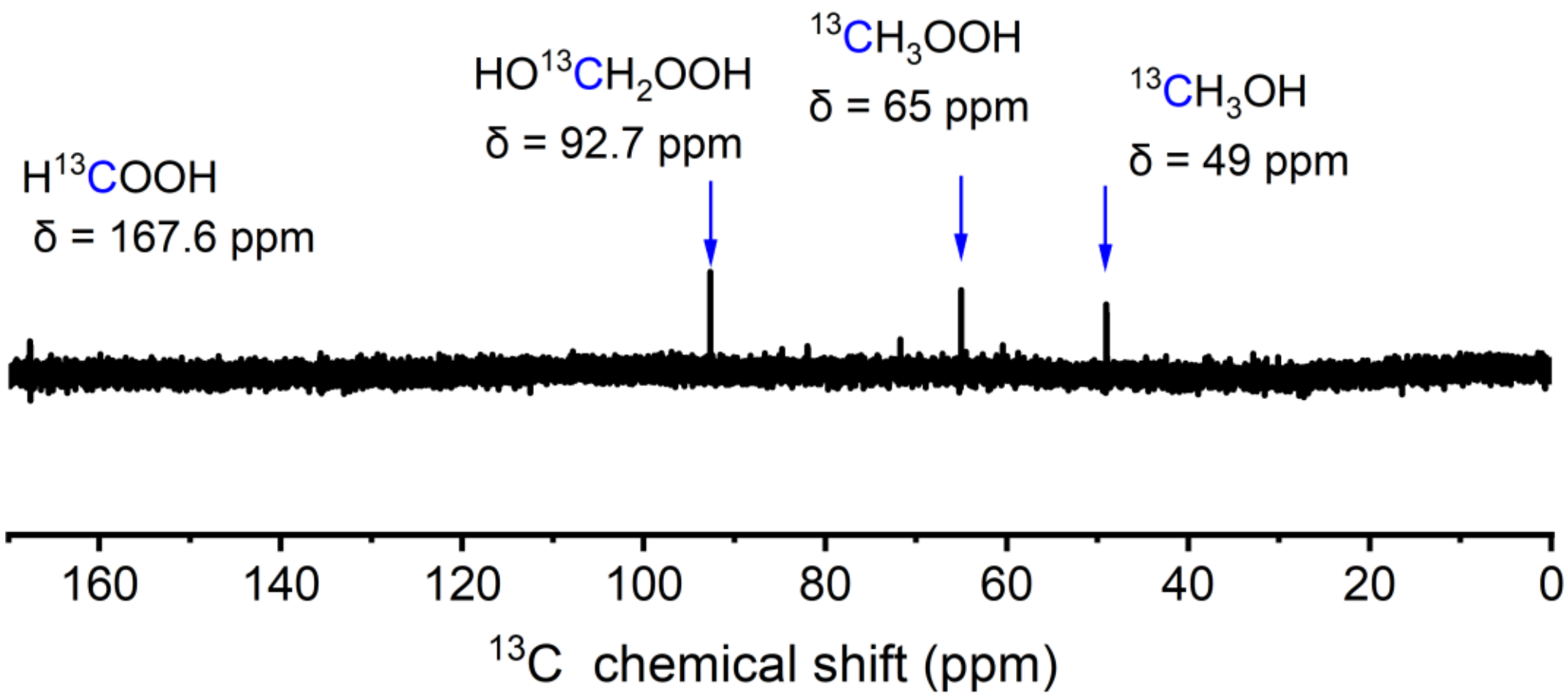

fig. S17. ^{13}C NMR spectrum of reaction products from $^{13}CH_4$ oxidation over Cu_1/U6-NO_2. Test conditions: Pressure (CH_4) = 2.5 MPa, Pressure ($^{13}CH_4$) = 0.5 MPa, Catalyst: 5 mg 0.04Cu_1/U6-NO_2, 1 M H_2O_2, 60 ºC, reaction for 4 h.

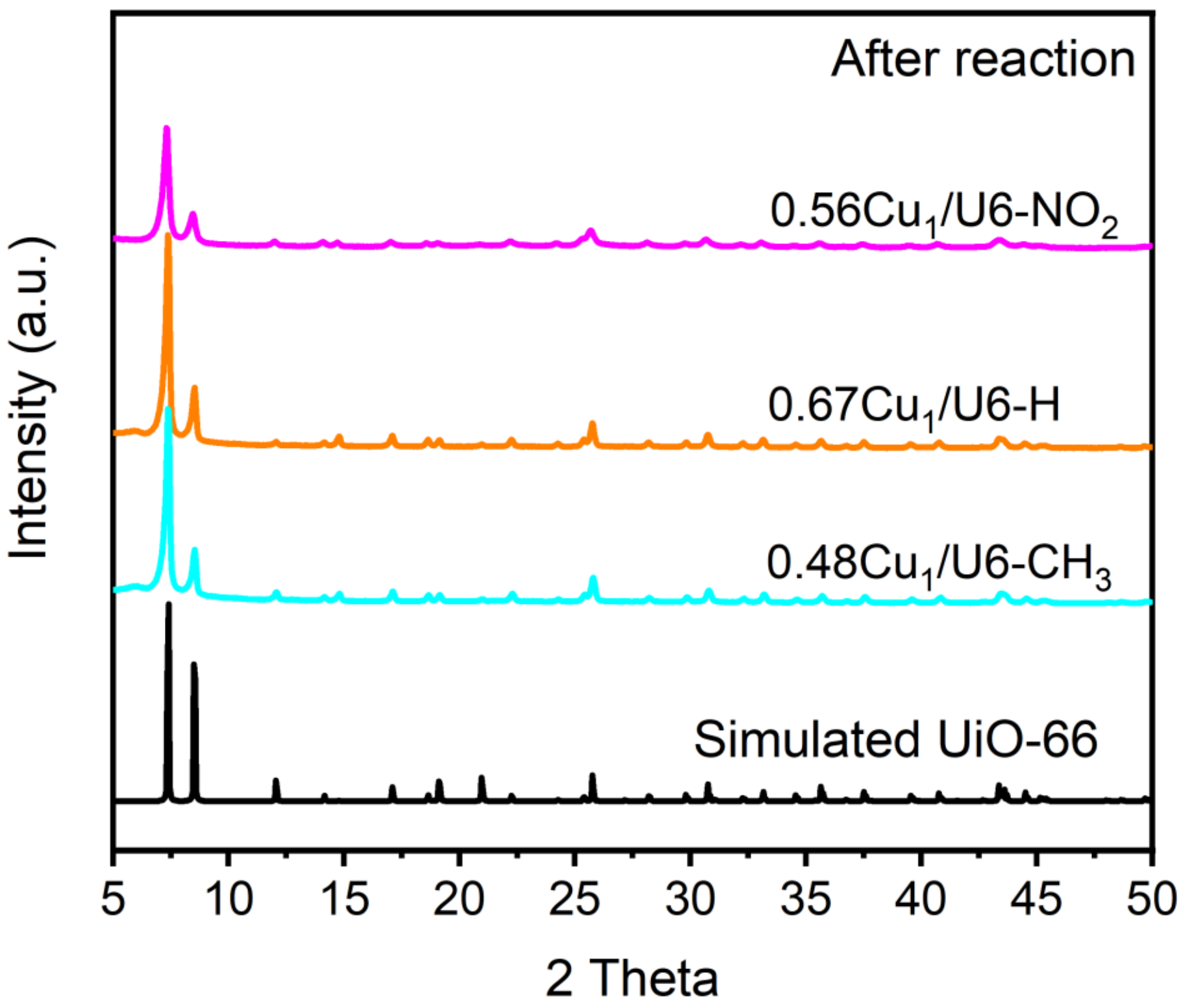

fig. S18. XRD pattern of Cu_1/U6-X catalysts after DSOM reaction.

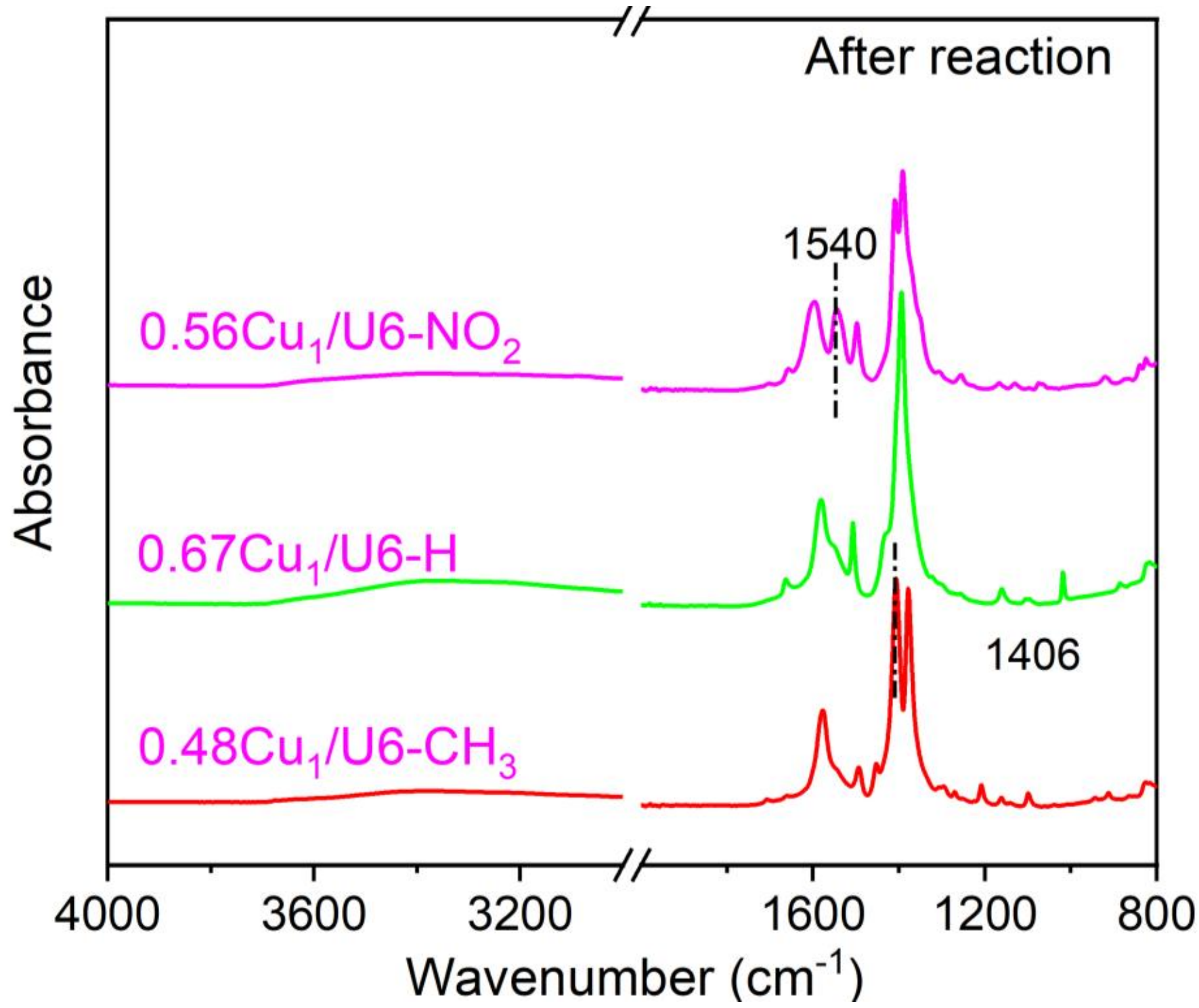

fig. S19. ATR-IR profiles of Cu_1/U6-X catalysts after DSOM reaction.

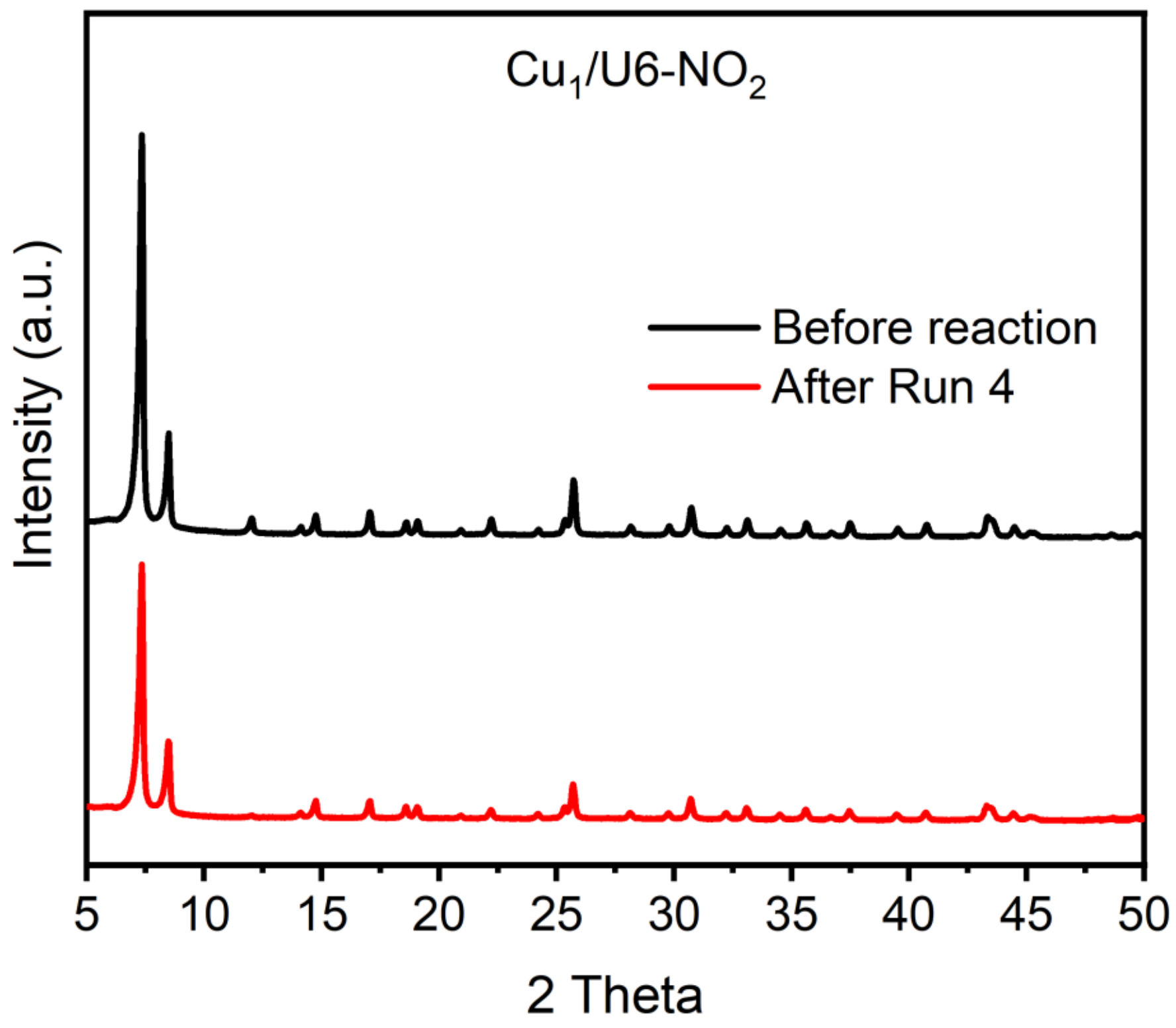

fig. S20. XRD pattern of 0.56Cu_1/U6-NO_2 catalysts before and after 4 cycle tests.

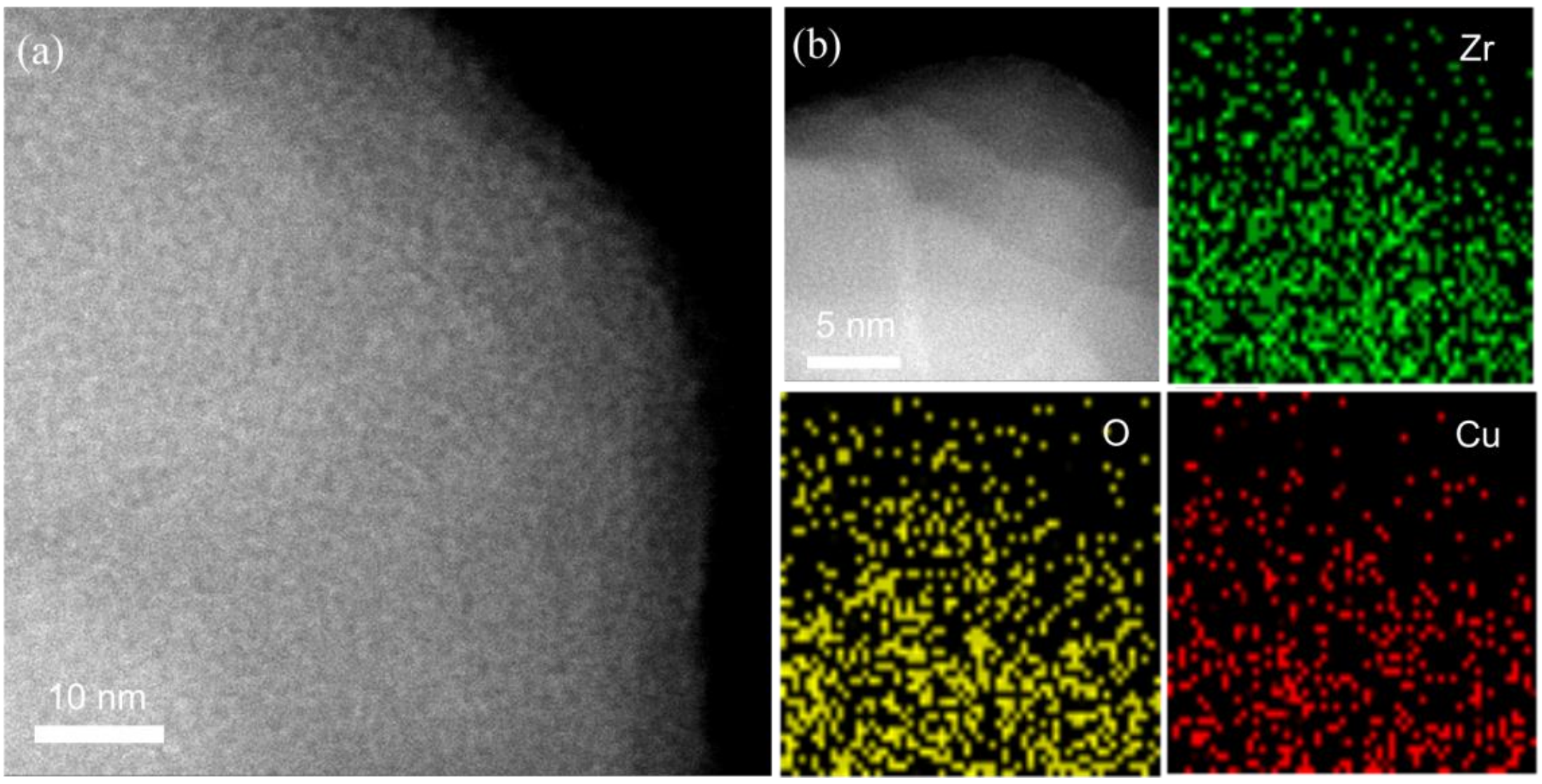

fig. S21. (a) Representative AC-HAADF-STEM image, (b) EDS element mapping for the distribution of Cu, O, and Zr elements on spent 0.56Cu_1/U6-NO_2 catalyst.

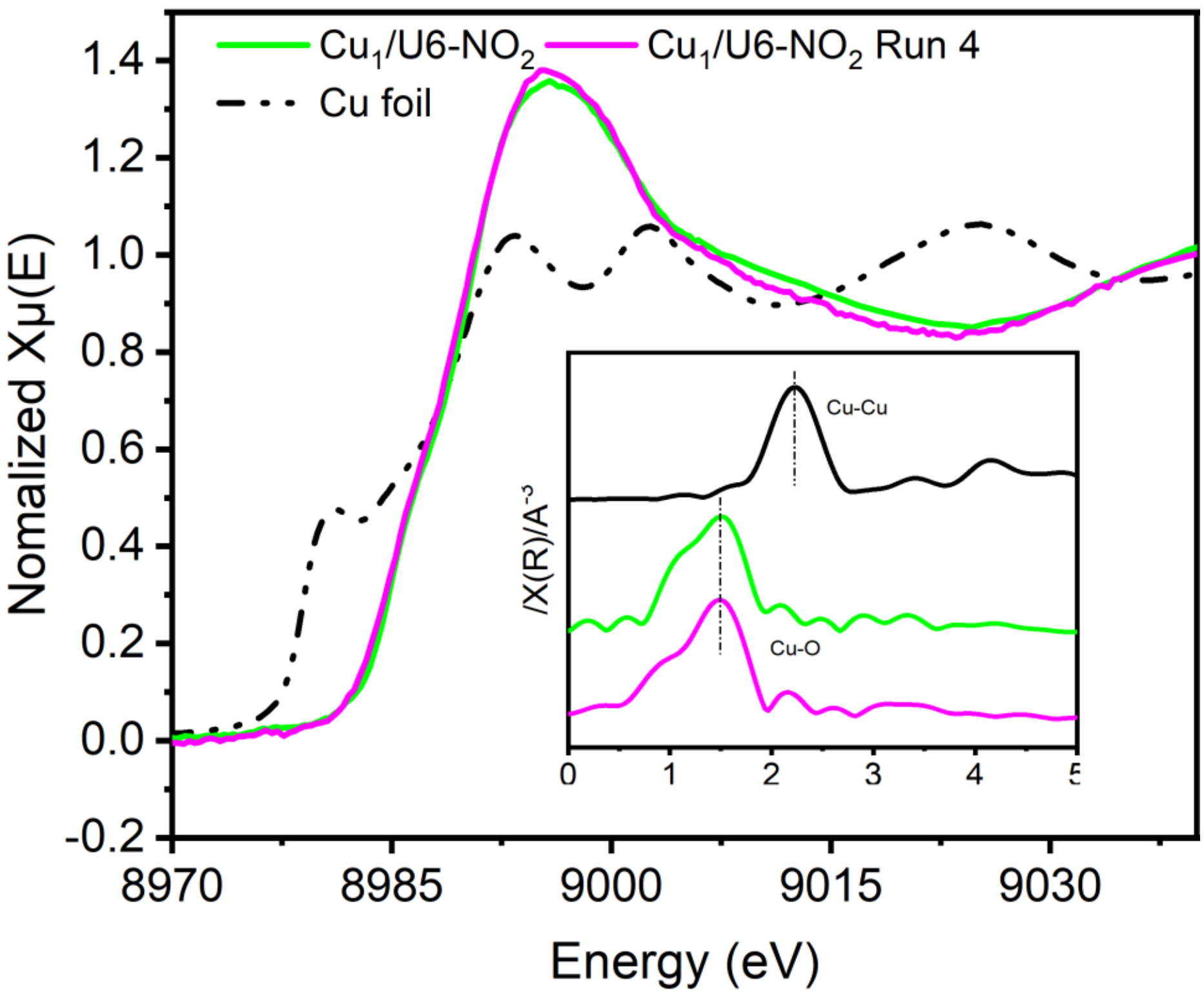

fig. S22. XANES spectra (inset: k^2-weighted Fourier transform spectra derived from EXAFS of the Cu k-edge) for Cu foil and 0.56Cu_1/U6-NO_2 catalysts before and after 4 cycle tests.

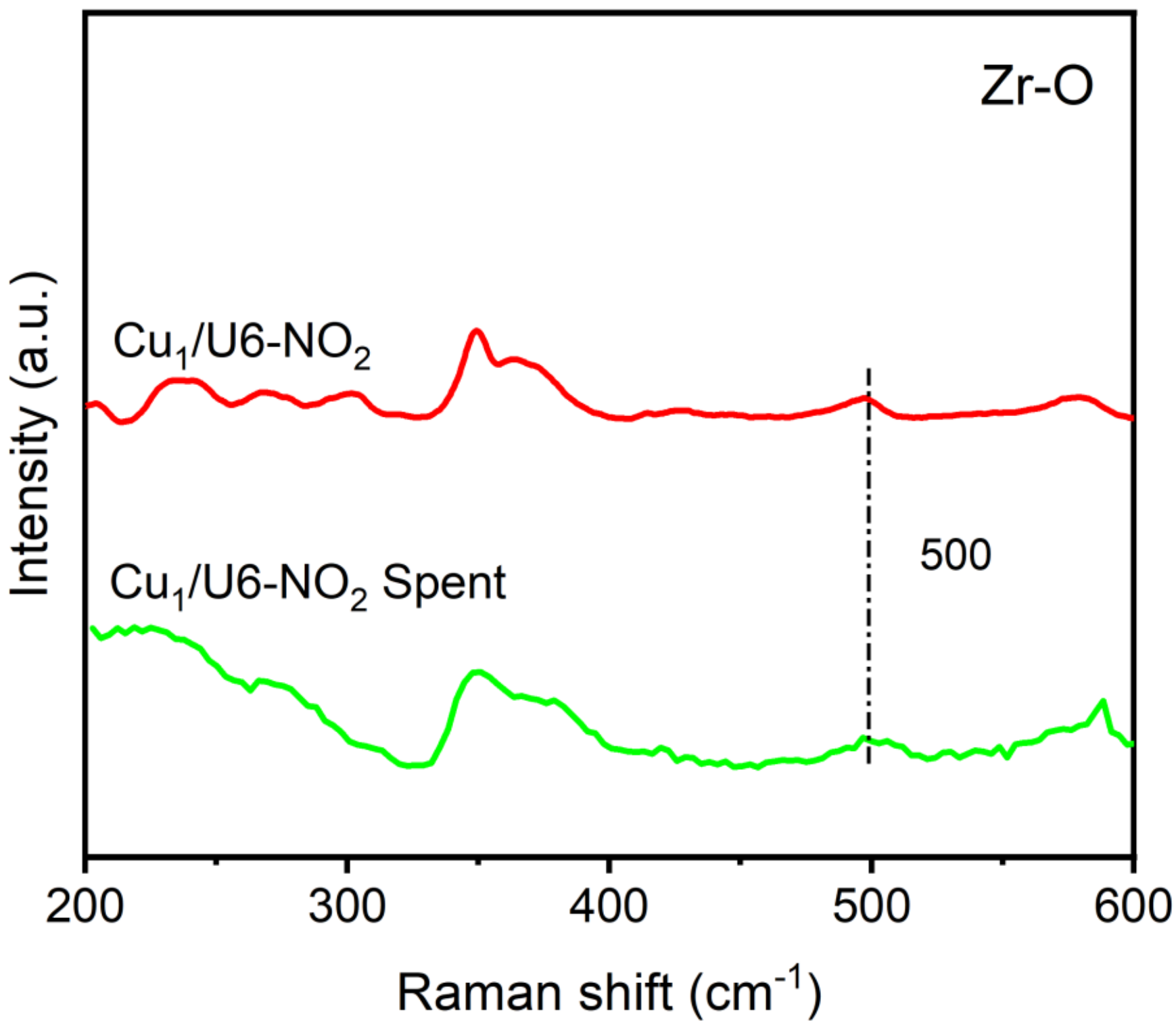

fig. S23. Raman spectra of Zr-O bond vibration for 0.56Cu$_1$/U6-NO$_2$ catalysts before and after 4 cycle tests.

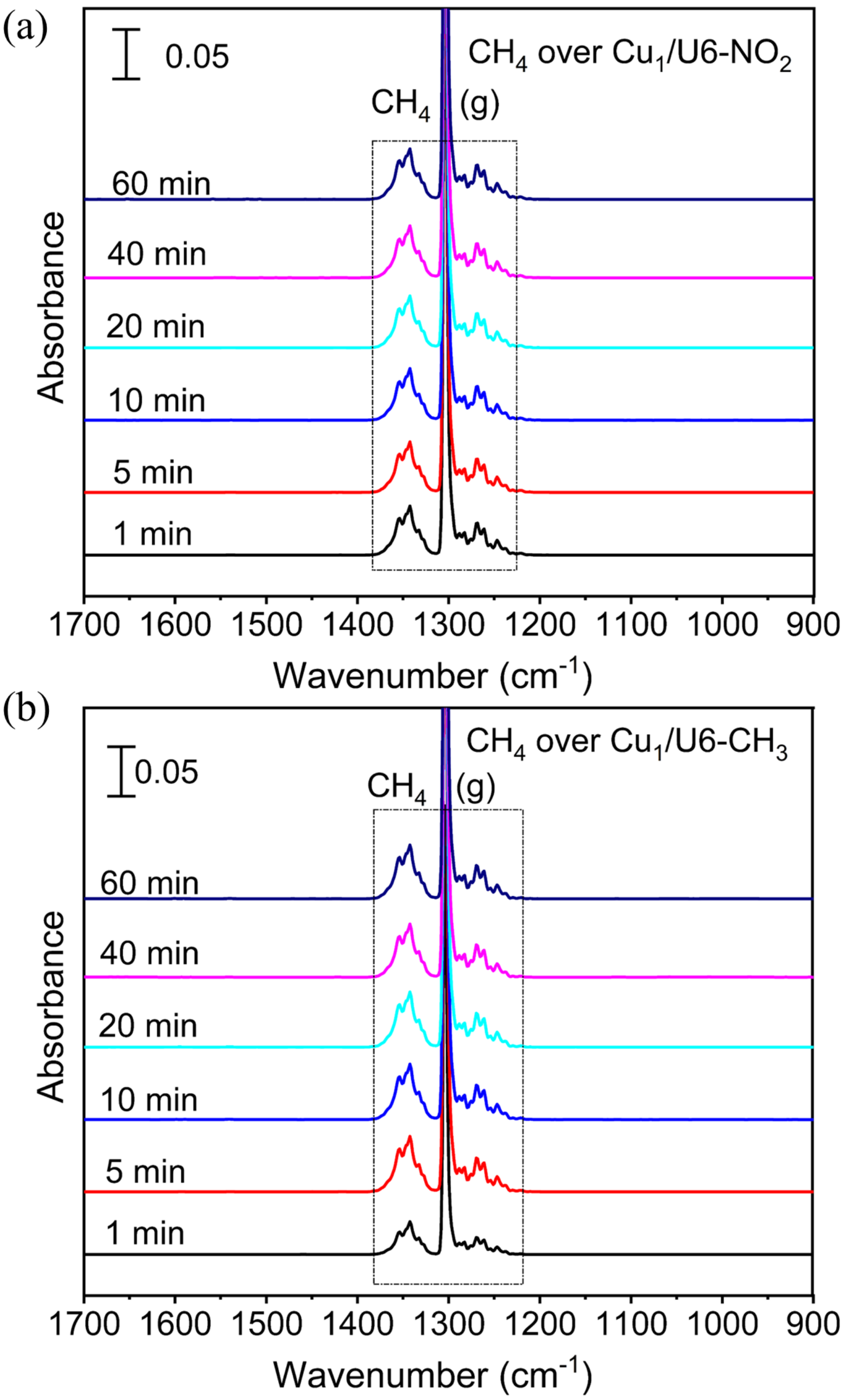

fig. S24. ***In situ*** **DRIFTS studies on the (A) 0.56Cu$_1$/U6-NO$_2$ and (B) 0.48Cu$_1$/U6-CH$_3$ catalysts at 60 °C under introducing CH$_4$.**

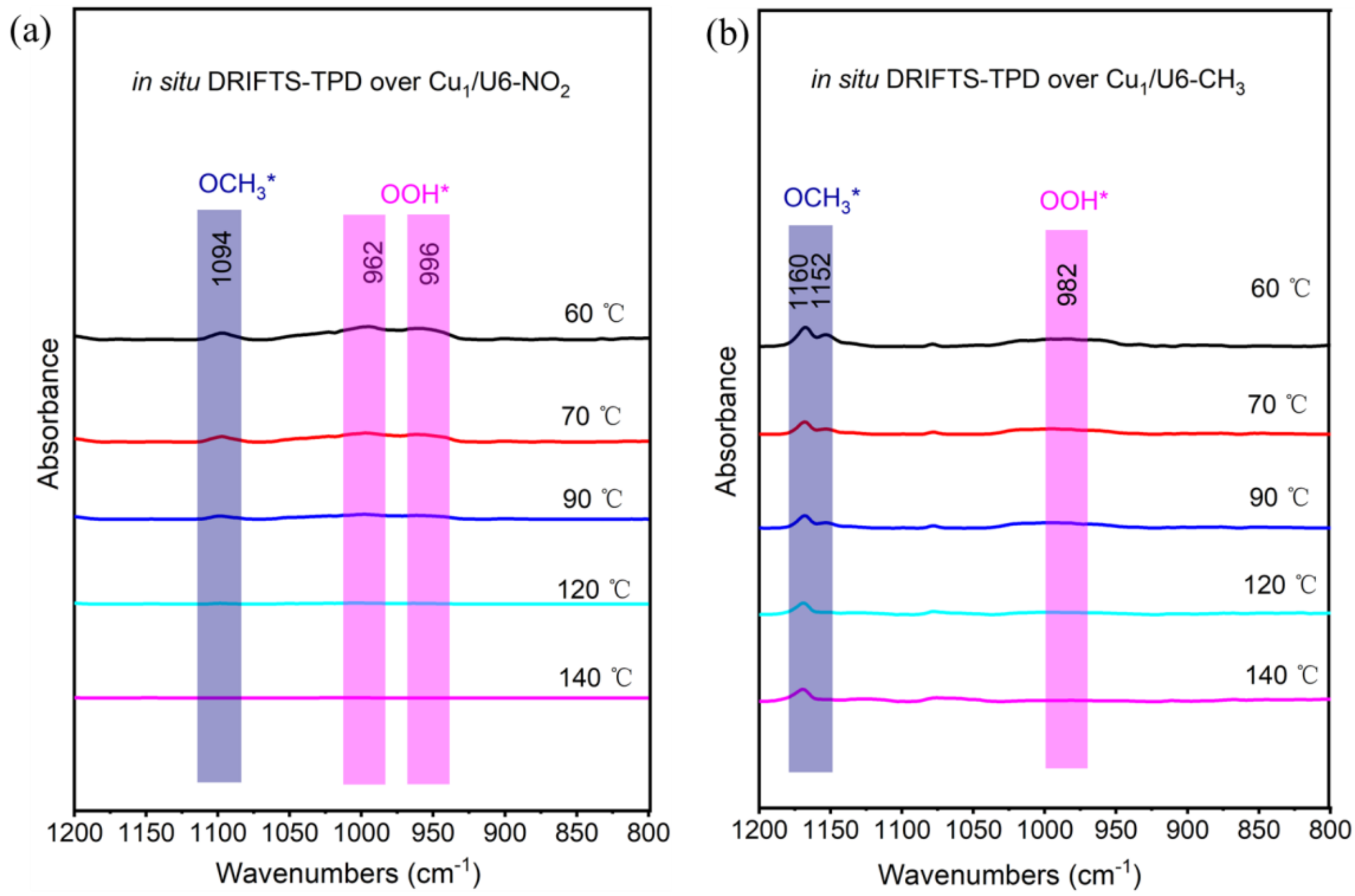

fig. S25. ***In situ*** **DRIFTS-TPD studies on the (A) 0.56Cu_1/U6-NO_2 and (B) 0.48Cu_1/U6-CH_3 catalysts after the adsrption of "CH_4 + H_2O_2".**

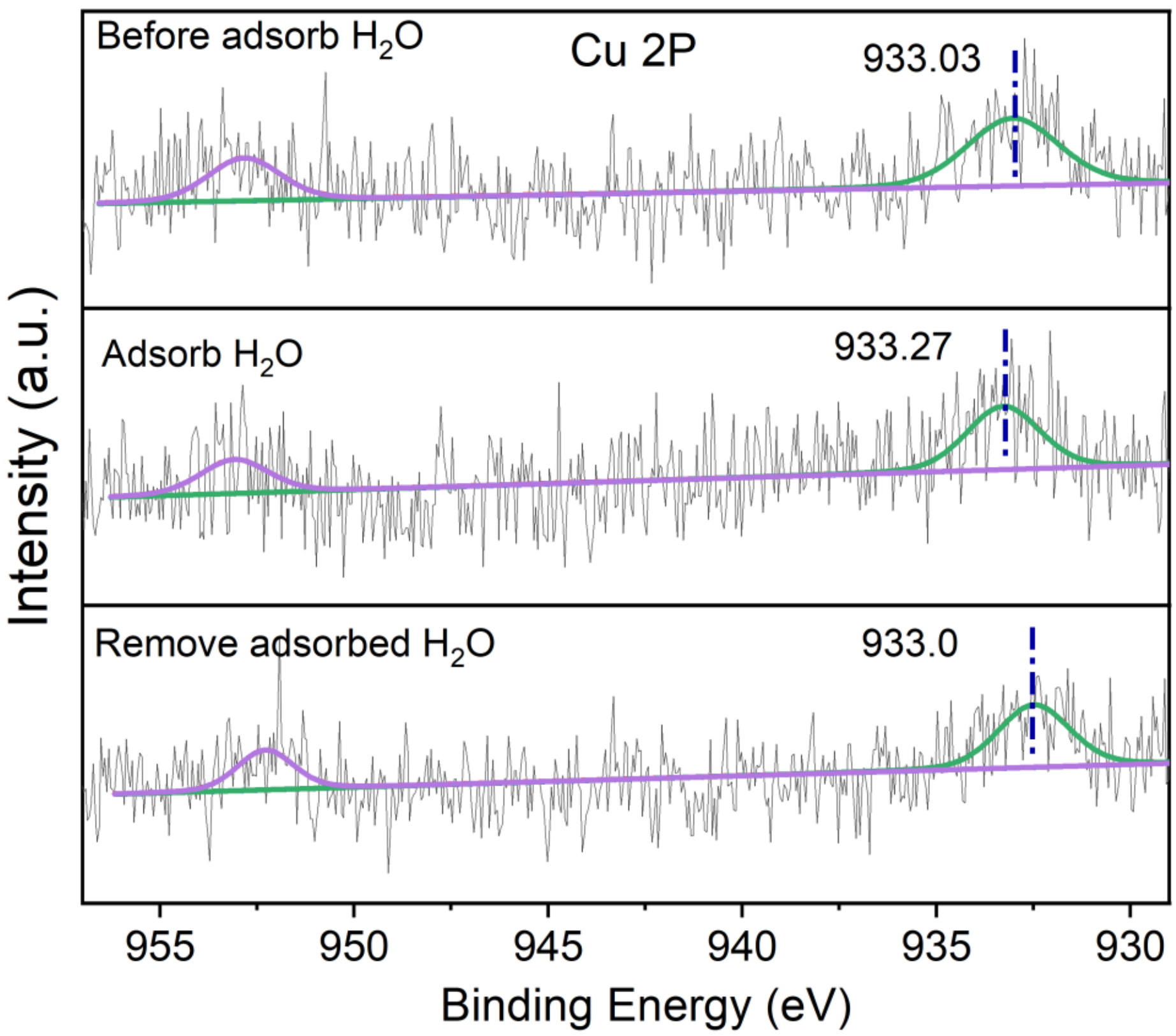

fig. S26. NAP-XPS studies on the 0.56Cu_1/U6-NO_2 catalysts under the H_2O vapor conditions.

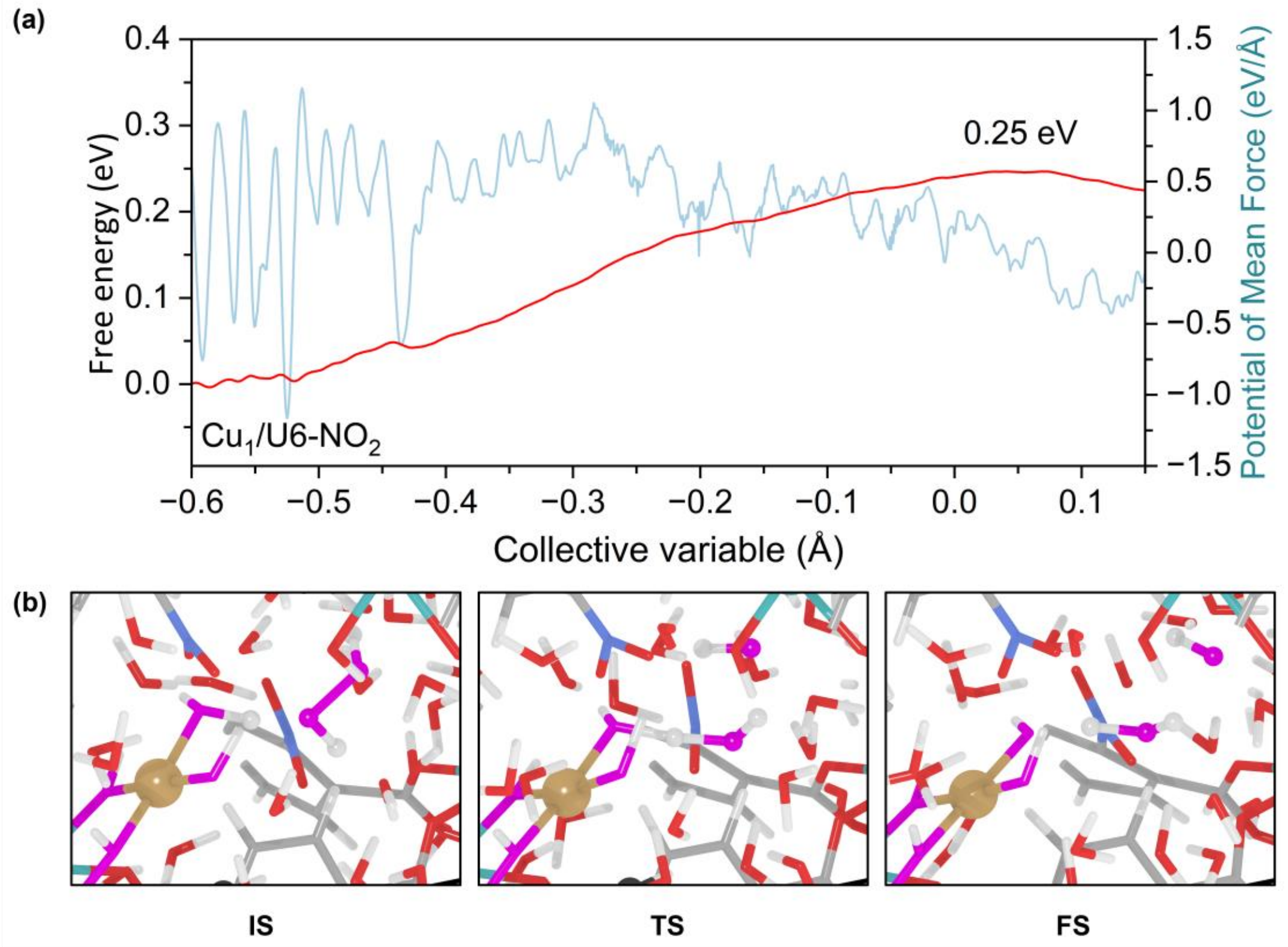

fig. S27. (A) Free energy profile along the reaction coordinate for H-abstraction from Cu_1-H_2O by H_2O_2. (B) Optimized structures of the initial state (IS), transition state (TS), and final state (FS). Color scheme: H (white), C (gray), O (red), O (coordinated to Cu, pink), N (blue), Cu (orange), Zr (cyan).

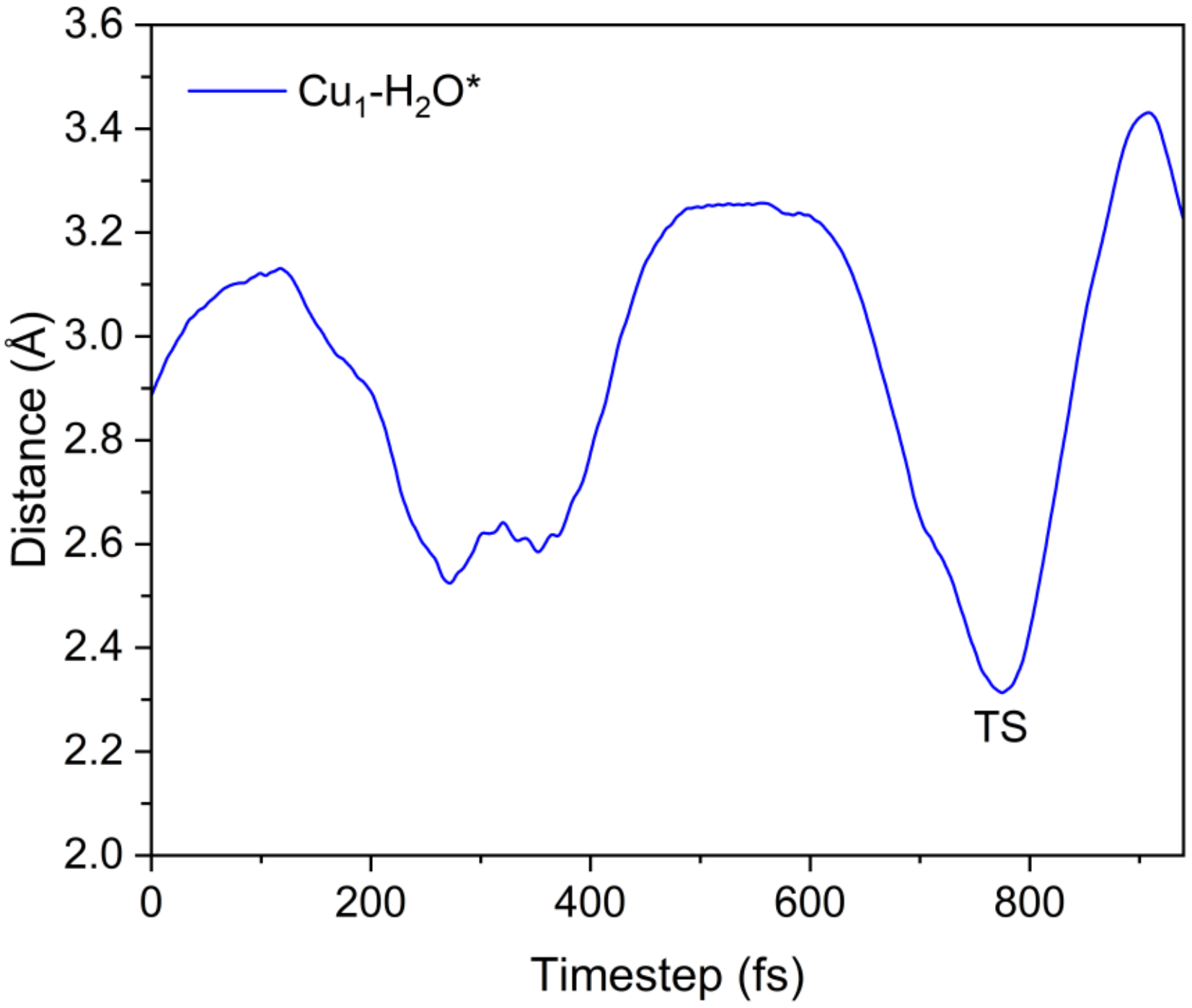

fig. S28. Evolution of Cu_1–H_2O* distances during H-abstraction from strongly chemisorbed Cu_1–H_2O* by H_2O_2 in Cu_1/U6-NO_2, showing lateral approach of weakly adsorbed water molecules to stabilize the transition state.

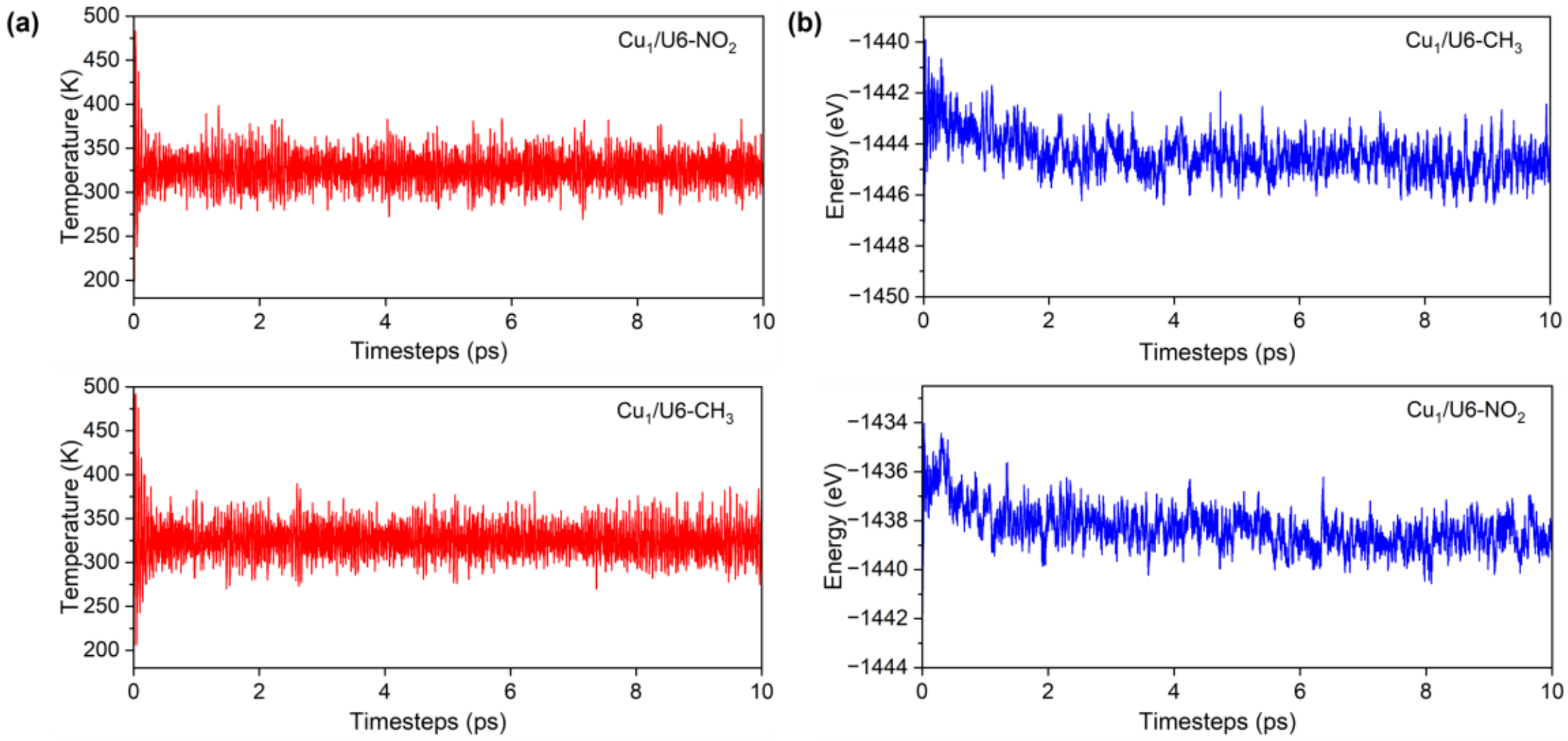

fig. S29. (A) Temperature and (B) energy evolution during the 10 ps AIMD equilibration of Cu_1/U6-NO_2 and Cu_1/U6-CH_3 in an aqueous solid–liquid interface environment.

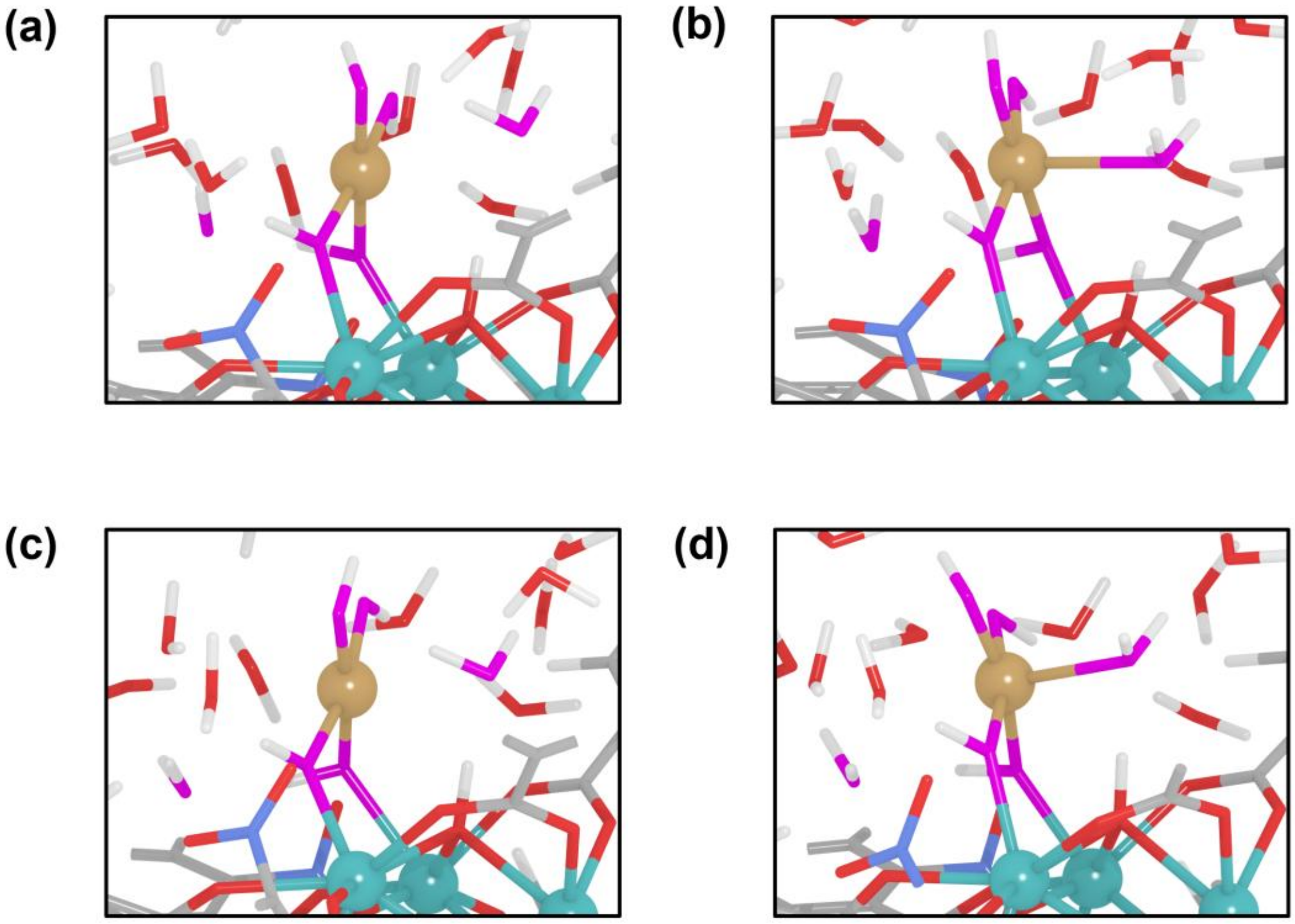

fig. S30. Four representative snapshots of H_2O* adsorption geometries around Cu_1 in Cu_1/U6-NO_2 from AIMD simulations at (A) 2.8 ps, (B) 3.6 ps, (C) 5.3 ps, and (D) 7.0 ps.

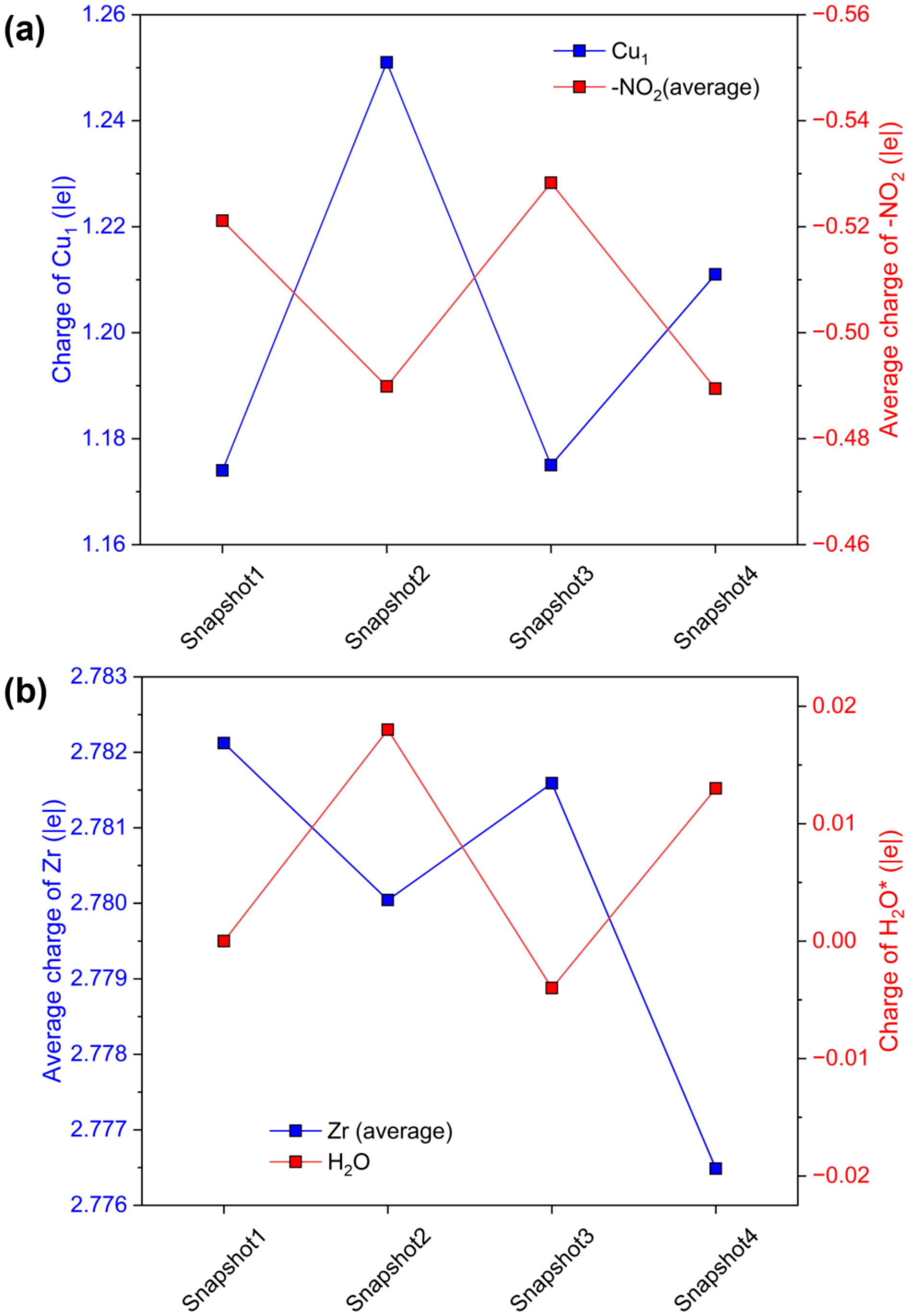

fig. S31. Dynamic charge evolution in Cu_1/U6-NO_2 from AIMD simulations. (A) Cu_1 centers and the -NO_2 group. (B) Zr atoms in the Zr-oxo node and adsorbed H_2O. Data correspond to four snapshots at 2.8, 3.6, 5.3, and 7.0 ps.

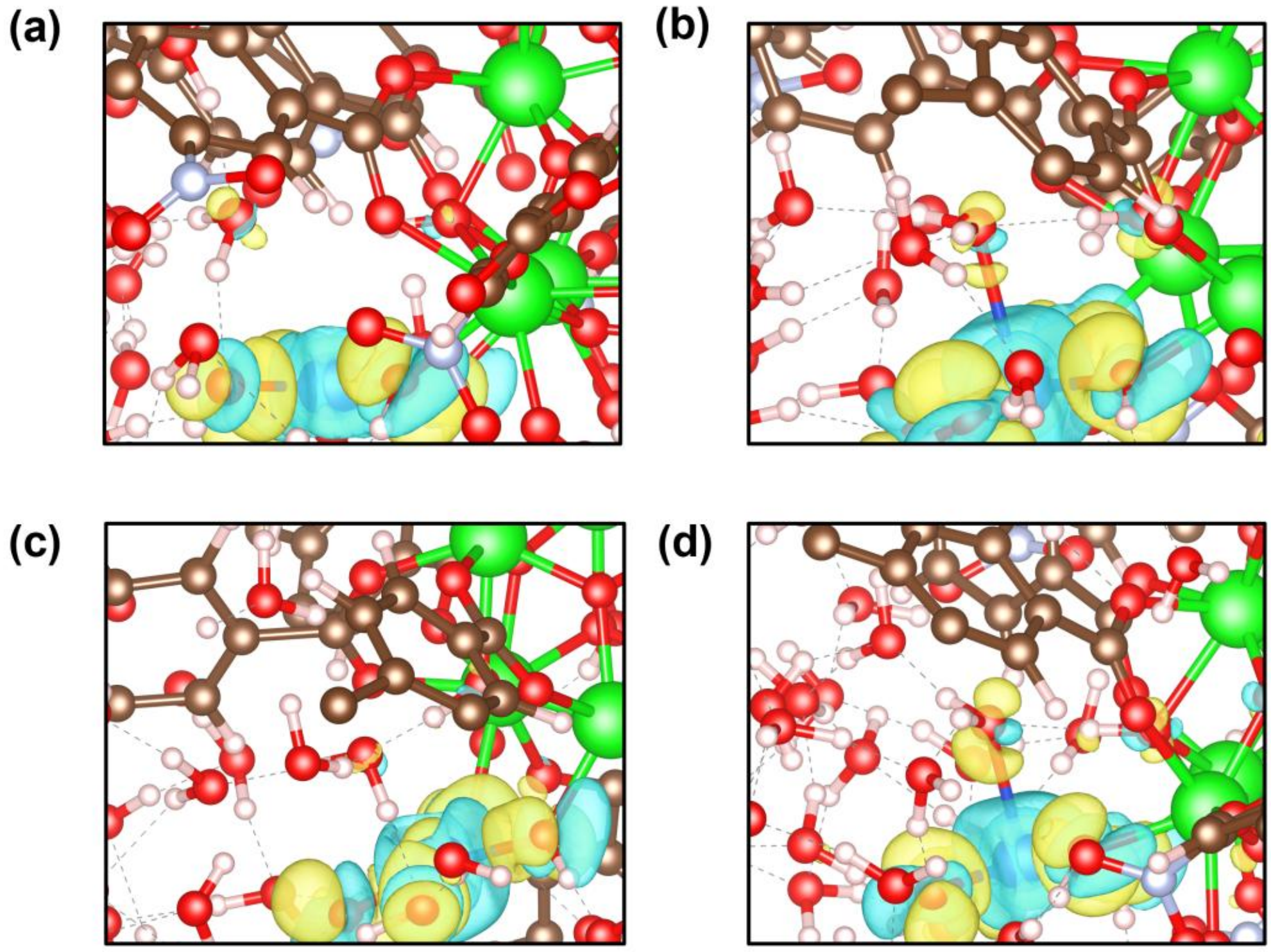

fig. S32. Charge density difference between Cu_1 and its coordination environment at (A) 2.8 ps, (B) 3.6 ps, (C) 5.3 ps, and (D) 7.0 ps snapshots from AIMD simulations of Cu_1/U6-NO_2. Yellow and cyan isosurfaces (0.003 e/$Bohr^3$) represent electron accumulation and depletion, respectively, showing dynamic electron transfer between H_2O and Cu_1. Color scheme: H (white), C (brown), O (red), N (pale blue), Cu (blue), Zr (green).

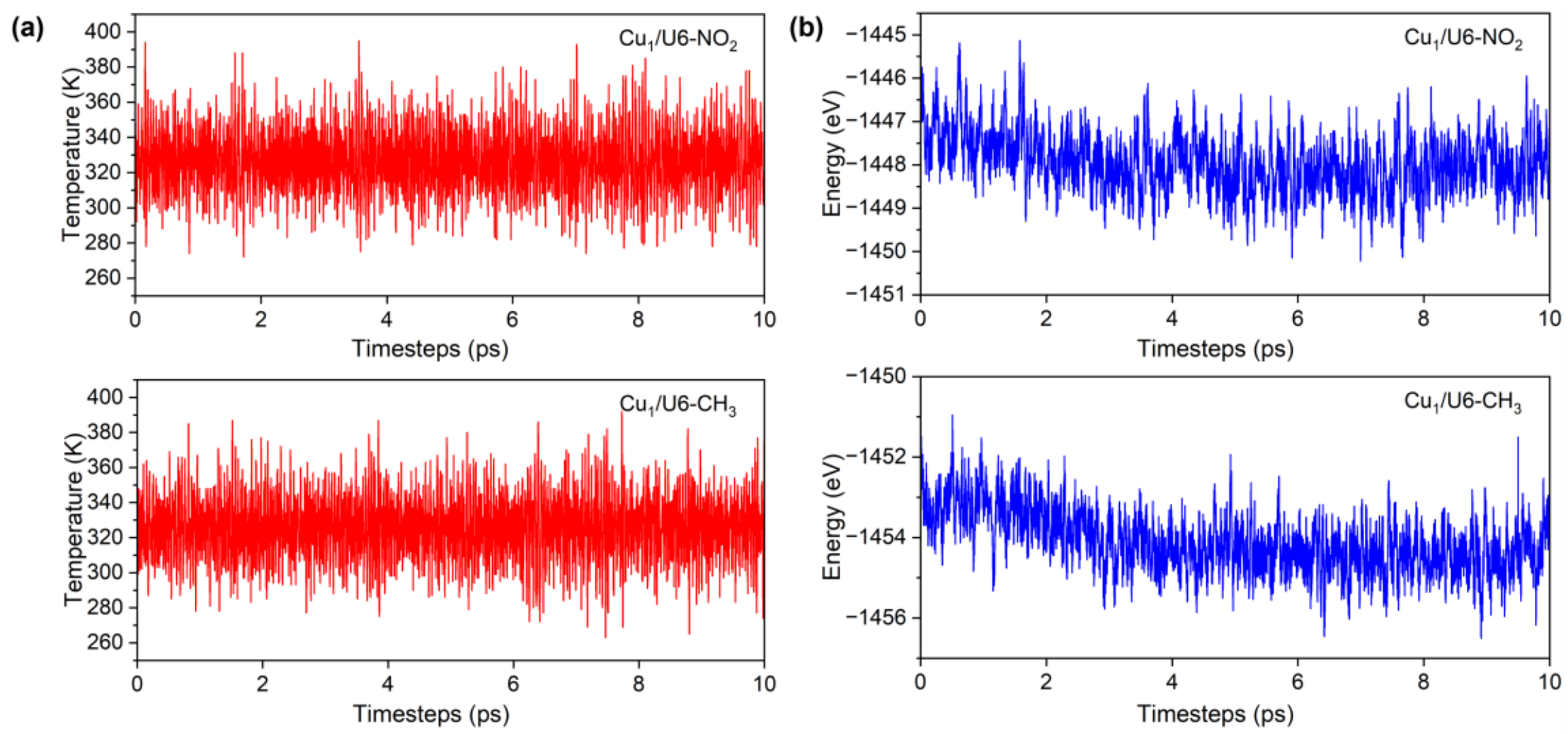

fig. S33. (A) Temperature and (B) energy evolution during the 10 ps AIMD equilibration of Cu_1/U6-NO_2 and Cu_1/U6-CH_3 in an aqueous solid–liquid interface environment upon the introduction of methane.

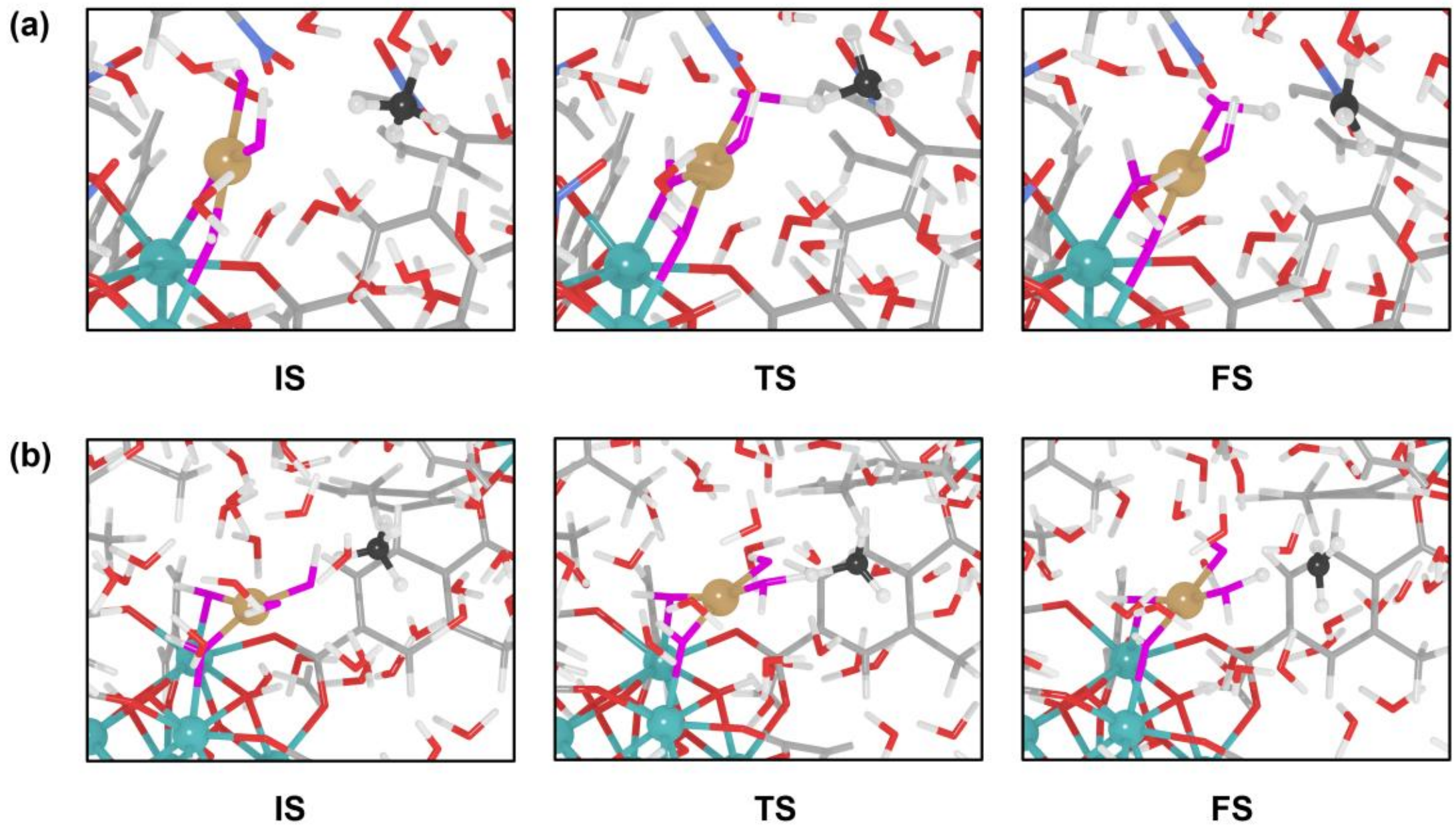

fig. S34. Structures of the initial state (IS), transition state (TS), and final state (FS) of C-H bond breaking at Cu_1-$^{\bullet}OH^*$ in (A) Cu_1/U6-NO_2 and (B) Cu_1/U6-CH_3. Color scheme: H (white), C (gray), O (red), O (coordinated to Cu, pink), N (blue), Cu (orange), Zr (cyan).

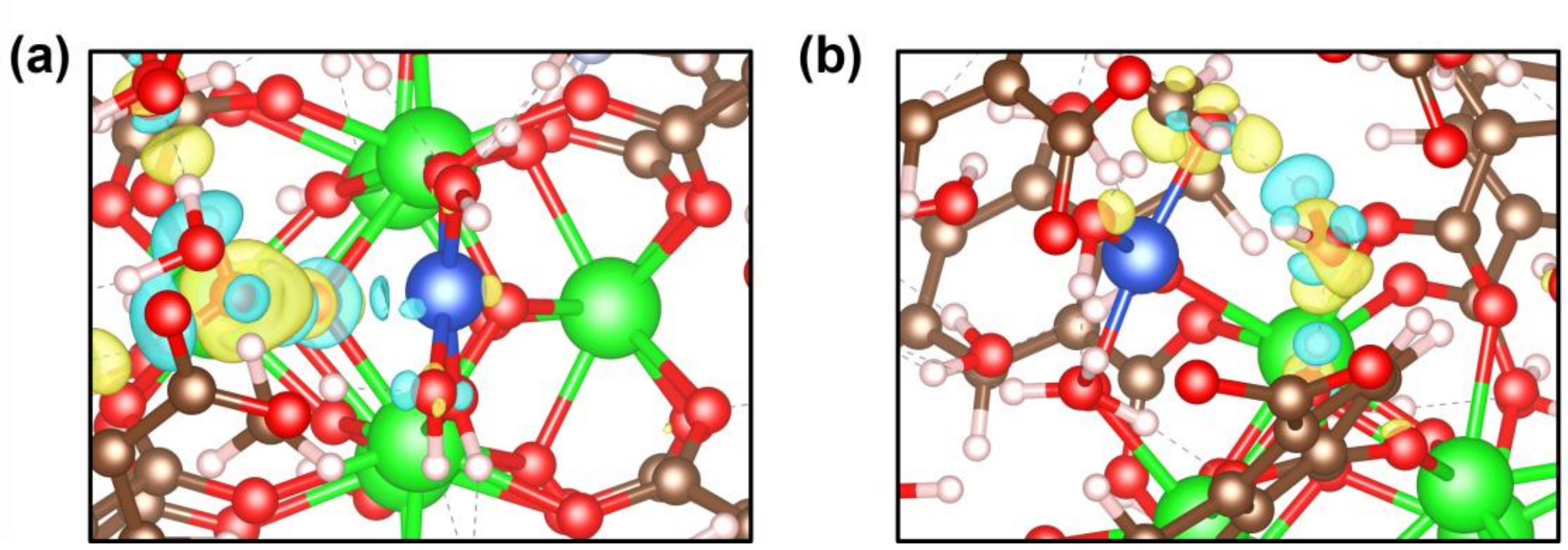

fig. S35. Charge density difference between H_2O (closest to Cu_1) and its coordination environment in the transition state of C-H bond breaking of CH_4 in (A) Cu_1/U6-NO_2 and (B) Cu_1/U6-CH_3. Yellow and cyan isosurfaces (0.003 e/Bohr^3) indicate electron accumulation and depletion, respectively. Color scheme: H (white), C (brown), O (red), N (pale blue), Cu (blue), Zr (green).

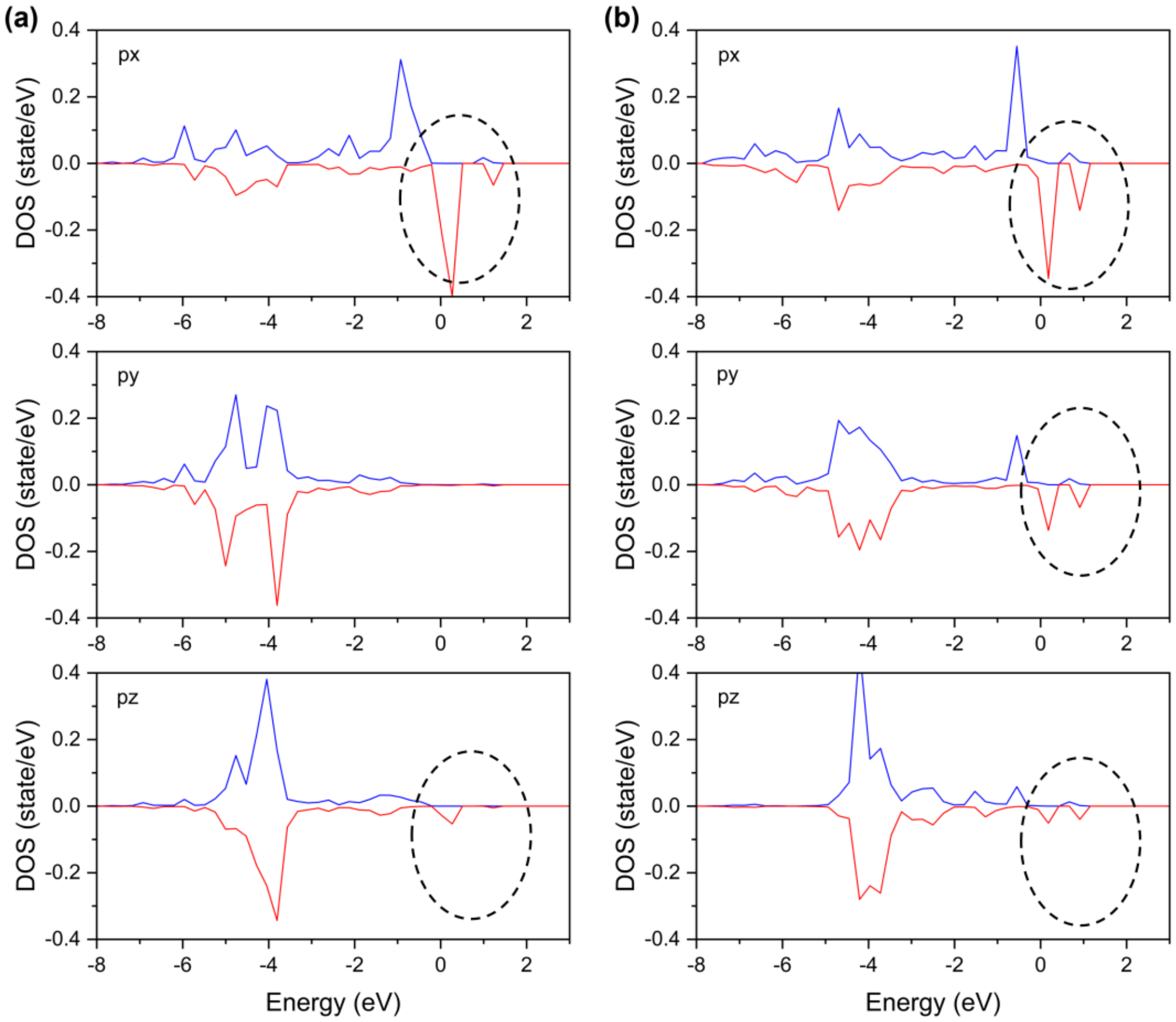

fig. S36. Projected density of state (PDOS) of the C atom in the ˙CH_3 at the transition state of C-H bond breaking at Cu_1-˙OH* in (A) Cu_1/U6-NO_2 and (B) Cu_1/U6-CH_3. Black circles mark unoccupied energy bands in the down-spin channel above the Fermi level.

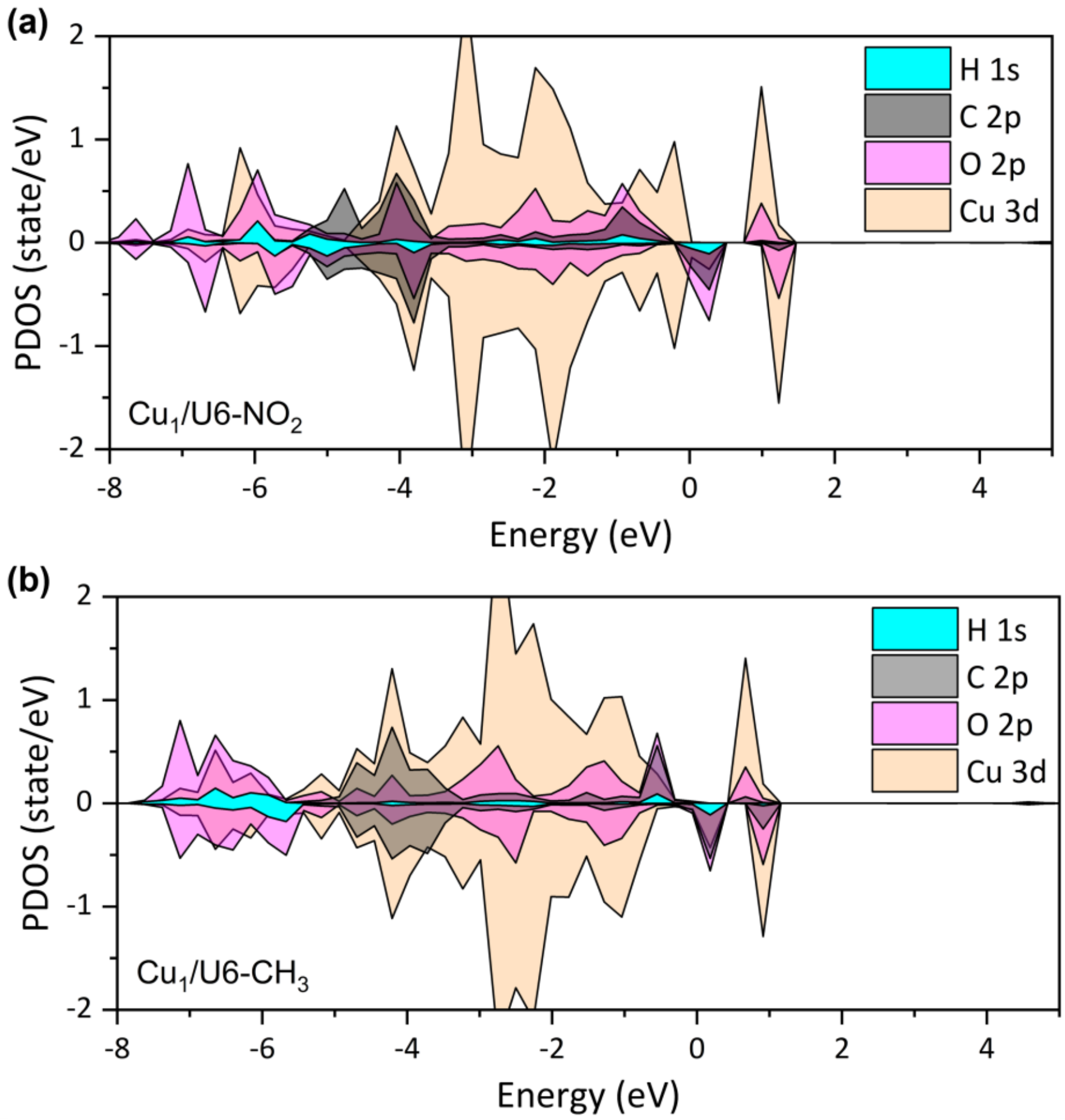

fig. S37. Projected density of states (PDOS) for key atomic orbitals in the transition state of C-H bond breaking at Cu_1-˙OH* (A) Cu_1/U6-NO_2 and (B) Cu_1/U6-CH_3, showing the 1s state of H, 2p states of C and O, and 3d state of Cu. Compared to Cu_1/U6-CH_3, Cu_1/U6-NO_2 exhibits stronger H-C orbital overlap but weaker O-H and Cu-O orbital overlaps.

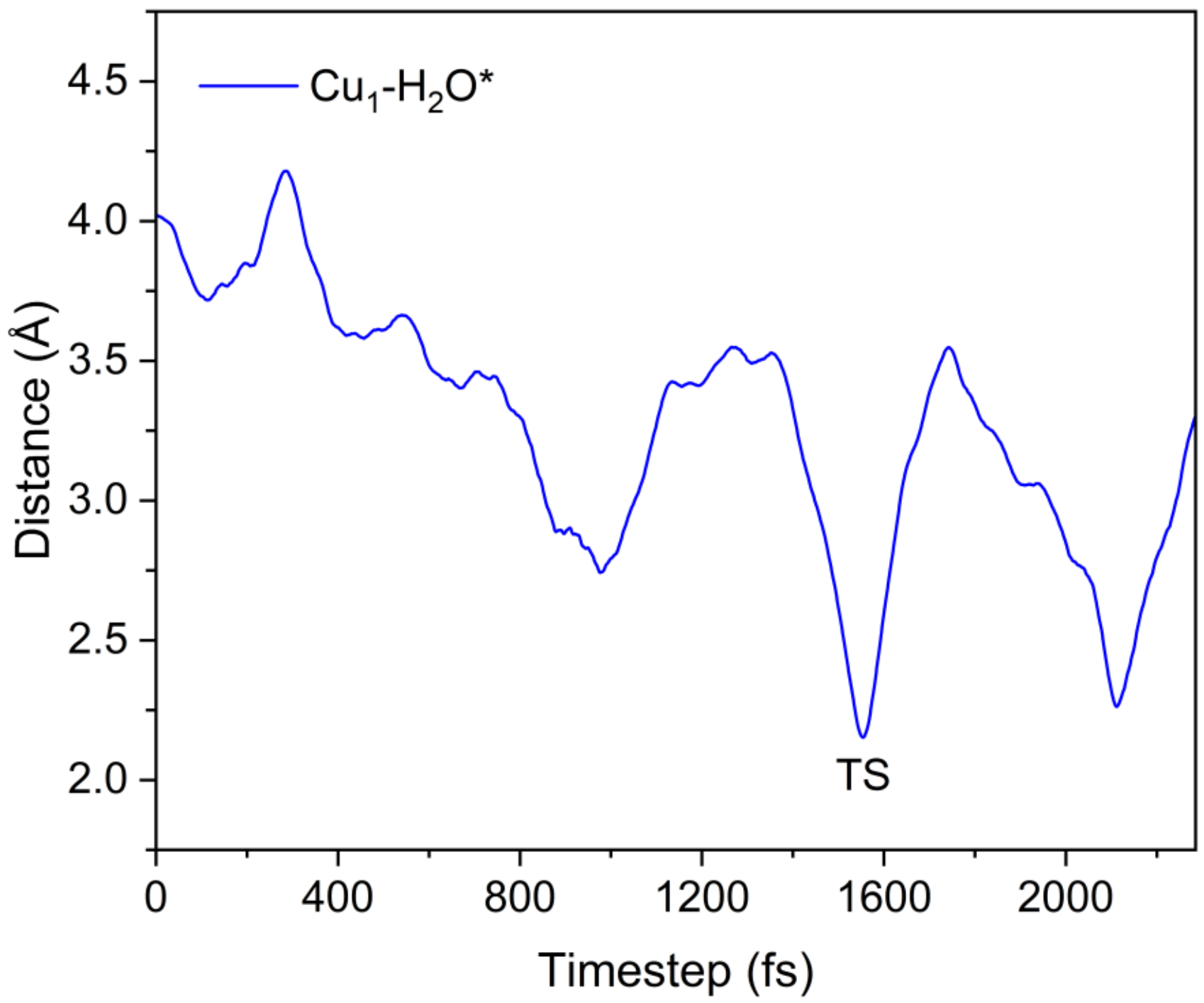

fig. S38. Evolution of the Cu_1-H_2O* distance during C–H activation in Cu_1/U6-NO_2, showing lateral approach of weakly adsorbed water molecules to stabilize the transition state.

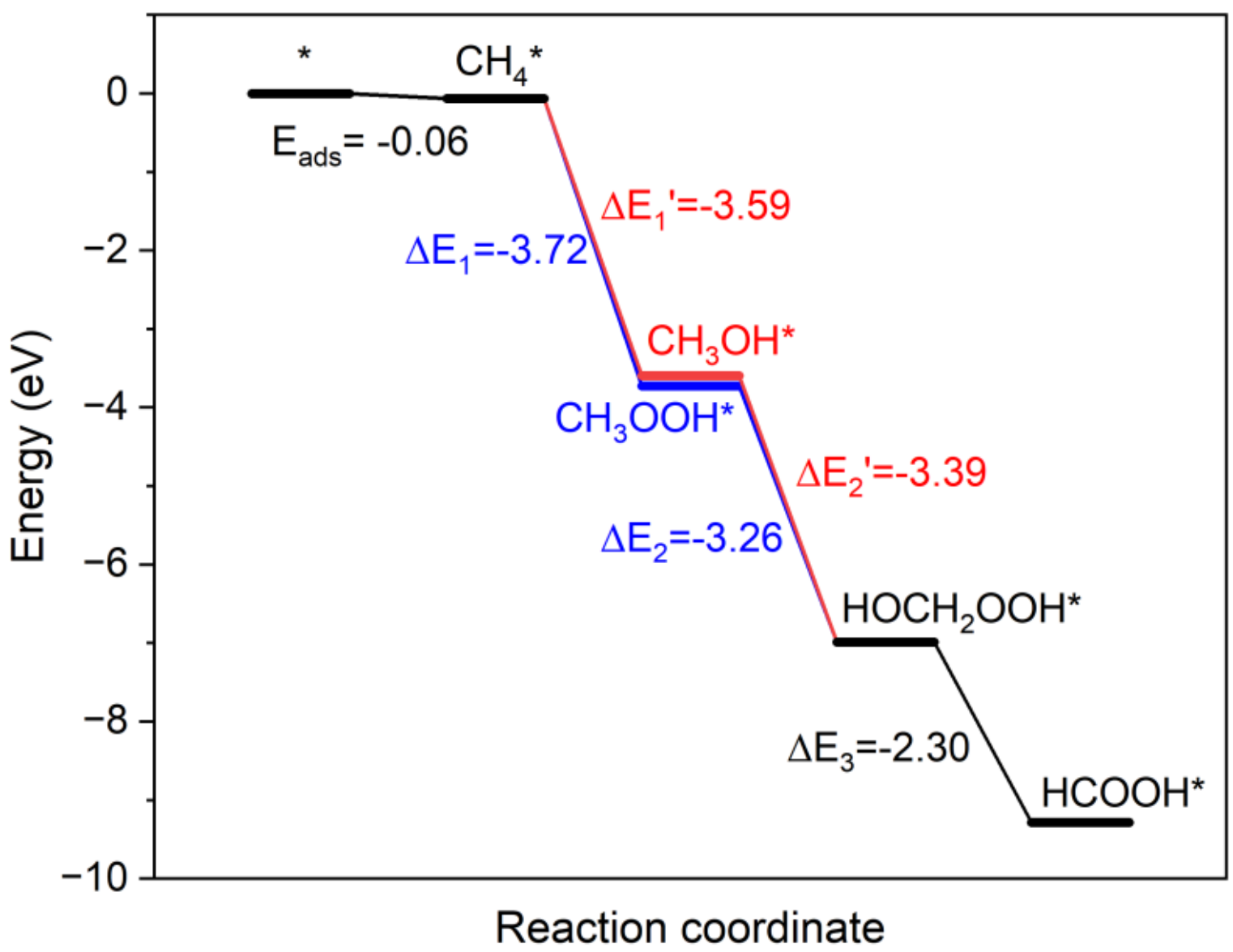

fig. S39. Calculated energy profile for the CH_4 oxidation pathway via ˙OOH and ˙OH species over Cu_1/U6-NO_2.

table S1. The ICP-OES results of different catalysts.

Catalyst	Metal loading (wt.%)
$0.56Cu_1/U6\text{-}NO_2$	0.56
$0.35Cu_1/U6\text{-}NO_2$	0.35
$0.04Cu_1/U6\text{-}NO_2$	0.04
$Cu_1/U6\text{-}NO_2$ Spent	0.54
$0.67Cu_1/U6\text{-}H$	0.67
$0.33Cu_1/U6\text{-}H$	0.33
$0.05Cu_1/U6\text{-}H$	0.05
$0.48Cu_1/U6\text{-}CH_3$	0.48
$0.35Cu_1/U6\text{-}CH_3$	0.35
$0.05Cu_1/U6\text{-}CH_3$	0.05

table S2. ICP-OES and EDS analysis of Cu loading of representative Cu_1/U6-X.

Catalyst	Cu Loading (wt.%)	
	ICP-OES	EDS[a]
0.56Cu_1/U6-NO_2	0.56	0.59
0.67Cu_1/U6-H	0.67	0.76
0.48Cu_1/U6-CH_3	0.48	0.50

[a]Values are derived from semi-quantitative EDS analysis of the selected mapping area. Cu K, Energy (8.04 keV)

table S3. EXAFS fitting parameters of the Cu-based samples.

Sample	Path	CN	R(Å)	σ^2(10^{-3}Å^2)	ΔE_0 (eV)	R factor
Cu_1/U6-CH_3[b]	Cu-O	3.94 ± 1.38	1.94 ± 0.03	4.8 ± 4.8	-0.39 ± 4.54	0.010
Cu_1/U6-H[c]	Cu-O	3.81 ± 1.44	1.93 ± 0.04	5.6 ± 5.3	-4.17± 4.85	0.013
Cu_1/U6-NO_2[d]	Cu-O	4.18 ± 1.68	1.95 ± 0.04	7.6 ± 6.6	-2.52 ± 5.32	0.018
Cu foil[e]	Cu-Cu	12*	2.54 ± 0.004	8.9 ± 0.4	4.19 ± 0.56	0.003
CuO[f]	Cu-O	2*	1.97 ± 0.01	0.8 ± 0.7	9.37 ± 1.27	0.010

CN, coordination number; R, bonding distance; σ^2, the Debye−Waller factor; ΔE_0, inner potential shift, S_0^2 was fixed to 0.90 as determined from Cu foil fitting. [a]Number with an asterisk indicates fixed coordination number (*N*) according to the crystal structure. [b]Fitting range: 3 ≤ k (Å) ≤ 10 and 1.0 ≤ R (Å) ≤ 2.0; [c]Fitting range: 2.0 ≤ k (/Å) ≤ 10 and 1.0 ≤ R (Å) ≤ 2.0; [d]Fitting range: 2.0 ≤ k (/Å) ≤ 10 and 1.0 ≤ R (Å) ≤ 2.0; [e]Fitting range: 2.5 ≤ k (/Å) ≤ 14 and 1 ≤ R (Å) ≤ 3; [f]Fitting range: 3.0 ≤ R (Å) ≤ 10.5 and 1.2 ≤ R (Å) ≤ 2.0

table S4. Textural properties of various $Cu_1/U6\text{-}X$ catalysts.

Catalyst	S_{BET}[a] ($m^2\ g^{-1}$)	V_t[b] ($cm^3\ g^{-1}$)	D_{pore}[c] (nm)
$Cu_1/U6\text{-}NO_2$	456.6	0.25	2.2
$Cu_1/U6\text{-}H$	1075	0.55	2.1
$Cu_1/U6\text{-}CH_3$	759.0	0.38	2.1
$Cu_1/U6\text{-}NO_2$ Spent	437.8	0.24	2.1

[a] Brunauer-Emmett-Teller surface area; [b] Single point pore volume at P/P_0=0.99; [c] The average pore diameter (BET method).

table S5. Performance comparison of Cu_1/U6-NO_2 with other catalysts in this work and previously reported representative catalysts for the DSOM reaction using or synthesizing H_2O_2 as the oxidant.

Catalyst	T (°C)	CH_4 (MPa)	H_2O_2 (M)	Oxygenates Sel. (%)	Oxygenates TOF (h^{-1})	Ref.
1AuPd/TiO_2	90	3.05	0.5	88.4	25.7	(*32*)
AuPd colloid	70	3	0.005	92	8.6	(8)
5AuPd@ZSM-5	70	0.05	H_2/He	88	87.6	(*33*)
2.5CuFe/ZSM-5	50	3.05	0.5	93	70	(*9*)
MIL-53(Fe, Al)	60	3.05	0.5	80	90	(*34*)
0.5Fe/MOR	50	3	0.5	91	89.6	(*35*)
Fe…Fe@FeS_2	r.t.	0.1	O_2	87	27.4	(*36*)
2.7Fe_1N_4/GN	r.t.	2	5	94	0.5	(*37*)
0.01Pd_1/ZSM-5+2CuO	95	3	0.5	95	166.8	(*38*)
0.3Rh_1/ZrO_2	70	3	0.5	78	1.3	(*39*)
0.29Rh_1/CeO_2 NWs	50	3.05	0.5	90.3	127	(*40*)
0.2Rh_1/TiO_2+$CuCl_2$	150	2.3	O_2/CO	99	66.5	(*41*)
0.68CNT@PNC/Ni_1	50	2	5	94.2	1.4	(*42*)
1Cu_1/C_3N_4	r.t.	3	4	95	11	(*43*)
0.34Cu_1/ZSM-5	50	3	0.5	99	157	(44)
0.68Cu_1/ZiF-7	50	3	0.5	-	1.6	(*45*)
1Cr_1/TiO_2	50	3	0.5	93	57.9	(*46*)
1.1Cr_1/ZSM-5	50	3	0.5	99.8	99.8	(*47*)
0.28Fe_1/ZIF-8	50	3	0.5	96.8	45	(*48*)
0.13Ru_1/UiO-66	60	3	1	~100	185.4	(*25*)
0.1Pd_1/ZSM-5	r.t.	3	0.6	~100	207	(*49*)
**0.04Cu_1/U6-NO_2**	**60**	**3**	**1**	**~100**	**322.4**	**This work**

table S6. The production of oxygenates for the DSOM reaction on Cu_1/U6-X catalysts under different feed gases.

Catalyst	Oxygenates (μmol g_{cat}^{-1} h^{-1})	
	CH_4	N_2
U6-NO_2	306.6	0
U6-H	369.0	0
U6-CH_3	115.2	0
0.04Cu_1/U6-NO_2	2029.2	0
0.05Cu_1/U6-H	1373.5	0
0.05Cu_1/U6-CH_3	970.4	0
0.35Cu_1/U6-NO_2	4191.5	0
0.33Cu_1/U6-H	3656.7	0
0.35Cu_1/U6- CH_3	2573.9	0
0.56Cu_1/U6-NO_2	5920.9	0
0.67Cu_1/U6-H	6102.4	0
0.48Cu_1/U6-CH_3	3881.7	33
NO_2-BDC	0	0
Blank	0	0

table S7. The intensity of the signal for $^{\bullet}OOH$ ($^{\bullet}O_2^-$) in EPR spectra over Cu_1/U6-X catalysts.

Catalyst	Intensity of $^{\bullet}OOH$ ($^{\bullet}O_2^-$) radical signal (a.u.)[a]						Total Intensity (a.u.)
	I	II	III	IV	V	VI	
Cu_1/U6-NO_2	238324.6	296348.6	5387.6	263927.6	7928.6	175070.6	986987.6
Cu_1/U6-CH_3	164038.2	199509.2	5766.2	177314.2	13840.2	100918.2	661386.2

[a]The intensity of $^{\bullet}OOH$ ($^{\bullet}O_2^-$) sextuple peak (I-VI) signal in EPR spectra.(*44*)

table S8. The intensity of the signal for ˙OH in EPR spectra over Cu_1/U6-X catalysts.

Catalyst	Intensity of hydroxyl radical signal (a.u.)[a]				Total Intensity (a.u.)
	I	II	III	IV	
Cu_1/U6-NO_2	769113.7	1505697.8	1485716.8	732600.7	4493129
Cu_1/U6-CH_3	1122033.5	2178292.5	2130263.5	1026785.5	6457375

[a]The intensity of ˙OH quadruple peak (I-IV) signal in EPR spectra.(*44*)

table S9. Cu_1-H_2O distances and Bader charges of Cu_1, two O(OH), -NO_2 ligand, Zr atoms, and adsorbed H_2O for four snapshots from AIMD simulations of Cu_1/U6-NO_2.

Structure	Snapshot 1	Snapshot 2	Snapshot 3	Snapshot 4
d(Cu-H_2O) (Å)	2.889	2.551	2.679	2.437
Cu_1 (|e|)	1.174	1.251	1.175	1.211
O(OH1) (|e|)	-1.025	-1.029	-1.061	-1.026
O(OH2) (|e|)	-1.050	-0.908	-1.046	-1.080
-NO_2 (|e|)	-0.521	-0.490	-0.528	-0.489
Zr (|e|)	2.782	2.780	2.782	2.776
H_2O (|e|)	0.000	0.018	-0.004	0.013

Movie S1. Water arrangement on Cu_1/U6-NO_2.

Movie S2. Water arrangement on Cu_1/U6-CH_3.

Movie S3. Cu_1-CH_4 distance in Cu_1/U6-NO_2.

Movie S4. Cu_1-CH_4 distance in Cu_1/U6-CH_3.
